# Efficacy of Reduced-Intensity Chemotherapy With Oxaliplatin and Capecitabine on Quality of Life and Cancer Control Among Older and Frail Patients With Advanced Gastroesophageal Cancer: The GO2 Phase 3 Randomized Clinical Trial

**DOI:** 10.1001/jamaoncol.2021.0848

**Published:** 2021-05-13

**Authors:** Peter S. Hall, Daniel Swinson, David A. Cairns, Justin S. Waters, Russell Petty, Christine Allmark, Sharon Ruddock, Stephen Falk, Jonathan Wadsley, Rajarshi Roy, Tania Tillett, Jonathan Nicoll, Sebastian Cummins, Joseph Mano, Simon Grumett, Zuzana Stokes, Konstantinos-Velios Kamposioras, Anirban Chatterjee, Angel Garcia, Tom Waddell, Kamalnayan Guptal, Nick Maisey, Mohammed Khan, Jo Dent, Simon Lord, Ann Crossley, Eszter Katona, Helen Marshall, Heike I. Grabsch, Galina Velikova, Pei Loo Ow, Catherine Handforth, Helen Howard, Matthew T. Seymour

**Affiliations:** 1 University of Leeds, Leeds, United Kingdom; 2 University of Edinburgh, Edinburgh, United Kingdom; 3 Leeds Teaching Hospitals National Health Service Trust, United Kingdom; 4 Maidstone and Tunbridge Wells National Health Service Trust, Maidstone, United Kingdom; 5 University of Dundee, Dundee, United Kingdom; 6 Bristol Oncology Centre, Bristol, United Kingdom; 7 Weston Park Cancer Centre, Sheffield, United Kingdom; 8 Hull University Hospitals National Health Service Trust, Hull, United Kingdom; 9 Royal United Hospitals Bath, Bath, United Kingdom; 10 North Cumbria University Hospitals National Health Service Trust, Carlisle, United Kingdom; 11 Royal Surrey County Hospital National Health Service Foundation Trust, Guildford, United Kingdom; 12 The Royal Wolverhampton National Health Service Trust, Wolverhampton, United Kingdom; 13 The Dudley Group National Health Service Foundation Trust, Dudley, United Kingdom; 14 United Lincolnshire Hospitals National Health Service Trust, Lincoln, United Kingdom; 15 Mid Yorkshire Hospitals National Health Service Trust, Wakefield, United Kingdom; 16 The Shrewsbury and Telford Hospital National Health Service Trust, Shrewsbury, United Kingdom; 17 Betsi Cadwaladr University Local Health Board, Bangor, United Kingdom; 18 The Christie National Health Service Foundation Trust, Manchester, United Kingdom; 19 Worcestershire Acute Hospitals National Health Service Trust, Worcester, United Kingdom; 20 Guys and St Thomas’s National Health Service Foundation Trust, London, United Kingdom; 21 York Teaching Hospital National Health Service Foundation Trust, Scarborough, United Kingdom; 22 Calderdale and Huddersfield National Health Service Foundation Trust, Huddersfield, United Kingdom; 23 University of Oxford, Oxford, United Kingdom; 24 Maastricht University Medical Center, Maastricht, the Netherlands

## Abstract

**Importance:**

Older and/or frail patients are underrepresented in landmark cancer trials. Tailored research is needed to address this evidence gap.

**Objective:**

The GO2 randomized clinical trial sought to optimize chemotherapy dosing in older and/or frail patients with advanced gastroesophageal cancer, and explored baseline geriatric assessment (GA) as a tool for treatment decision-making.

**Design, Setting, and Participants:**

This multicenter, noninferiority, open-label randomized trial took place at oncology clinics in the United Kingdom with nurse-led geriatric health assessment. Patients were recruited for whom full-dose combination chemotherapy was considered unsuitable because of advanced age and/or frailty.

**Interventions:**

There were 2 randomizations that were performed: CHEMO-INTENSITY compared oxaliplatin/capecitabine at Level A (oxaliplatin 130 mg/m^2^ on day 1, capecitabine 625 mg/m^2^ twice daily on days 1-21, on a 21-day cycle), Level B (doses 0.8 times A), or Level C (doses 0.6 times A). Alternatively, if the patient and clinician agreed the indication for chemotherapy was uncertain, the patient could instead enter CHEMO-BSC, comparing Level C vs best supportive care.

**Main Outcomes and Measures:**

First, broad noninferiority of the lower doses vs reference (Level A) was assessed using a permissive boundary of 34 days reduction in progression-free survival (PFS) (hazard ratio, HR = 1.34), selected as acceptable by a forum of patients and clinicians. Then, the patient experience was compared using Overall Treatment Utility (OTU), which combines efficacy, toxic effects, quality of life, and patient value/acceptability. For CHEMO-BSC, the main outcome measure was overall survival.

**Results:**

A total of 514 patients entered CHEMO-INTENSITY, of whom 385 (75%) were men and 299 (58%) were severely frail, with median age 76 years. Noninferior PFS was confirmed for Levels B vs A (HR = 1.09 [95% CI, 0.89-1.32]) and C vs A (HR = 1.10 [95% CI, 0.90-1.33]). Level C produced less toxic effects and better OTU than A or B. No subgroup benefited from higher doses: Level C produced better OTU even in younger or less frail patients. A total of 45 patients entered the CHEMO-BSC randomization: overall survival was nonsignificantly longer with chemotherapy: median 6.1 vs 3.0 months (HR = 0.69 [95% CI, 0.32-1.48], *P* = .34). In multivariate analysis in 522 patients with all variables available, baseline frailty, quality of life, and neutrophil to lymphocyte ratio were independently associated with OTU, and can be combined in a model to estimate the probability of different outcomes.

**Conclusions and Relevance:**

This phase 3 randomized clinical trial found that reduced-intensity chemotherapy provided a better patient experience without significantly compromising cancer control and should be considered for older and/or frail patients. Baseline geriatric assessment can help predict the utility of chemotherapy but did not identify a group benefiting from higher-dose treatment.

**Trial Registration:**

isrctn.org Identifier: ISRCTN44687907

## Key Points

**Question:**

Do older and/or frail patients with advanced gastroesophageal cancer benefit from less intensive palliative chemotherapy, and can a formal geriatric assessment assist treatment decision-making?

**Findings:**

This phase 3 randomized clinical trial including 559 patients with advanced gastroesophageal cancer found that reducing the intensity of chemotherapy provided an improved patient experience with no significant detriment in cancer control. Baseline frailty, quality of life, and neutrophil/lymphocyte ratio (an inflammation marker) were predictive of outcome and may contribute to treatment decisions.

**Meaning:**

Decision-making for older and/or frail patients with advanced cancer can be enhanced using geriatric assessment; such patients generally benefit from reducing the intensity of chemotherapy.

## Teaser

This randomized clinical trial assesses the efficacy of reduced-intensity chemotherapy for patient quality of life and cancer control in the treatment of older and/or frail patients.

## Introduction

Cancer is most common in older people. In North America and Europe, gastroesophageal cancer is the third most common cause of cancer death, with more than half these deaths in people over 75 years,^1^ many of whom are frail and with comorbidities. But evidence guiding treatment for vulnerable older patients is poor: standard chemotherapy regimens were developed in trials involving predominantly nonfrail, noncomorbid patients of median age less than 65,^2,3,4,5^ and although selected older people participated they cannot be assumed to fully represent the older population.

In 2011, MRC FOCUS2,^6^ a national randomized trial designed for frail and older patients with colorectal cancer, was reported. It used reduced doses of chemotherapy and introduced a novel composite end point, Overall Treatment Utility (OTU), combining clinical efficacy, tolerability, and the patient’s own assessment of treatment value and acceptability. In the same year we surveyed 50 gastrointestinal oncologists in the UK^7^: 49 reported routinely treating older patients with gastroesophageal cancer with standard schedules at reduced doses, or omitting agents. There was wide variation in practice and no use of objective geriatric assessment (GA) to guide decisions. This led to 321GO, a randomized feasibility trial in which older and/or frail patients with gastroesophageal cancer received 80% doses of the standard 3-drug schedule epirubicin/oxaliplatin/capecitabine (EOCap),^2^ the same treatment omitting epirubicin (OCap), or the same treatment omitting both epirubicin and oxaliplatin (Cap). The best balance of benefits and tolerability was achieved with the OCap doublet.^7^ This trial allowed further development of the OTU end point.^7,8^

The GO2 randomized clinical trial takes the OCap schedule from 321GO^7^ and compares 3 dose levels, seeking the best balance of efficacy and patient experience. For patients with uncertainty regarding whether to use chemotherapy at all, an alternative randomization compares the lowest dose level vs supportive care alone. We ask whether a baseline GA may aid personalized dose selection and perhaps identify patients unlikely to benefit from chemotherapy. More broadly, GO2 aims to stimulate researchers across all cancer types to evaluate patient-centered assessment, dosing, and outcome measurement for vulnerable patients.

## Methods

### Study Design and Participants

The GO2 trial (ISRCTN44687907) was an academic, multicenter, open-label randomized trial, approved by the UK National Research Ethics Service, overseen by independent Trial Steering and Data Monitoring & Ethics Committees. All participants gave fully informed written consent. The study is closed and follow-up is complete. The authors assume responsibility for accuracy, completeness, and fidelity to the trial protocol (Supplement 1) and statistical analysis plan (Supplement 2).

Eligibility criteria are detailed in the Trial Protocol. Patients had locally advanced and/or metastatic gastroesophageal cancer that was not pretreated. In the absence of established objective frailty thresholds, and given the complex interrelations of frailty and advanced age, we used oncologists’ clinical judgment in selecting patients. The key eligibility criterion was that the oncologist considered full-dose standard combination chemotherapy (at that time epirubicin/oxaliplatin/capecitabine^2^ or cisplatin/fluorouracil/trastuzumab^9^) unsuitable because of the patient’s advanced age and/or frailty. It was made clear to patients and clinicians that GO2 was a trial for older patients, but there were no chronological age limits. Furthermore, since scoring of performance status (PS) by oncologists in older patients is inconsistent,^10^ a fixed PS threshold was not specified, but patients had to be considered fit for any of the treatments in their selected randomization. Patients with moderate renal/hepatic dysfunction could be entered with dose adjustment to compensate for reduced clearance. Medical comorbidity was allowed provided the oncologist did not consider this to preclude chemotherapy. Response Evaluation Criteria in Solid Tumors–assessable disease was not mandatory.^11^

### Randomization

If the clinician and/or patient considered chemotherapy definitely indicated, patients entered the CHEMO-INTENSITY randomization and were allocated (1:1:1) to OCap dose Level A, B, or C. Level A treatment was oxaliplatin 130 mg/m^2^ on day 1 and capecitabine 625 mg/m^2^ twice daily on days 1-21, on a 21-day cycle; Level B treatment was 80% of Level A doses; and Level C treatment was 60% of Level A doses. If the patient and clinician agreed that best supportive care alone (BSC) would be an acceptable alternative, patients could instead enter the CHEMO-BSC randomization, with allocation (1:1) to OCap Level C or BSC. Randomization used an automated telephone/web system and validated minimization algorithm, with age, PS, metastases, histology, renal function, planned trastuzumab use, and center as stratification factors. The treatment allocation was not masked from study investigators or patients.

### Procedures

The GA, aligned with the European Organization for Research and Treatment of Cancer (EORTC) Elderly Minimum Data set,^12^ was administered after consent but before randomization; results were not communicated to the clinician. It included G8,^13^ Instrumental Activities of Daily Living (IADL),^14^ Timed Up and Go test,^15^ EORTC QLQ-C30/OG25,^16,17^ and EQ-5D and visual analogue scale (EQ-VAS).^18^ Frailty was scored by assessing impairment (yes/no) in nine domains (weight loss, mobility, falls, neuropsychiatric, physical functioning, social functioning, mood, fatigue, and polypharmacy) and participants were categorized as not frail (0-1/9 domain impaired), mildly frail (2/9 domains), or severely frail (≥3/9 domains).^19^

Patients with estimated glomerular filtration rate (eGFR) of 30 to 50 mL/min or bilirubin 1.5 to 2 times the upper limit of normal received 75% of their allocated dose of capecitabine. Patients with ERBB2 (formerly HER2)-positive cancers could additionally receive trastuzumab. Imaging was repeated every 9 weeks, and chemotherapy stopped in the event of radiological or clinical progression, unacceptable toxic effects, or patient choice. Patients allocated to BSC had access to specialist palliative care, pain and psychosocial services, blood transfusions, nutritional support, radiotherapy, stenting, or surgical procedures as indicated; chemotherapy, although not planned, was allowable if it later became indicated.

Overall Treatment Utility was scored once, 9 weeks after starting chemotherapy. It comprised computed tomography (CT) and clinical assessment of cancer progression status; toxic effects (Common Terminology Criteria for Adverse Events, CTCAE) and serious adverse events (SAEs); quality of life (QL, as scored with QLQ-C30 Global Health Status subscale), and patient value/acceptability, scored from 2 questions posed in a questionnaire before patients received their scan results: “Since you started chemotherapy, how worthwhile do you think your treatment has been?” and “How much has your chemotherapy interfered with your normal daily activities?” both scored “not at all/a little/quite a bit/very much.” Overall Treatment Utility was not measurable in patients allocated BSC.

Treatment beyond 9 weeks continued until CT progression or clinical/patient decision. Longitudinal QL comprised weekly EQ-VAS and every 3 weeks EQ-5D and QLQ-C30 Fatigue Subscale during chemotherapy, then then once every 9 weeks until a year from randomization.

### Outcomes

End points, conforming to the joint EORTC/Alliance/International Society of Geriatric Oncology (SIOG) Statement,^20^ are defined in the trial protocol (Supplement 1) and statistical analysis plan (Supplement 2). In the CHEMO-INTENSITY randomization the primary end point was investigator-determined progression-free survival (PFS).^21^ The key secondary end point was OTU. A score of “Good OTU” requires no radiological or clinical evidence of cancer progression, no major toxic effects (a serious adverse reaction [SAR], or any grade ≥3 non-hematological toxicity), no significant deterioration in QL (≥16 percentage-points drop in EORTC Global QL subscale^22^) and no adverse responses to patient value/acceptability questions (“not at all” worthwhile or “very much” interference). Poor OTU denotes evidence of cancer progression and at least 1 other negative factor (toxic effects, SAE, QL deterioration, or poor value/acceptability), or the patient has died. Intermediate OTU means either cancer progression without any other negative factor or negative factors without cancer progression. Other secondary end points were toxic effects; symptoms (QLQ-C30/OG250); QL; RECIST response^11^; overall survival (OS); and quality-adjusted survival. Fatigue was scored using QLQ-C30 with time-to-deterioration from randomization to a deterioration of 16 percentage points or more. In the CHEMO-BSC randomization, the primary end point was OS; secondary end points were patient-reported fatigue and QL.

### Statistical Analysis

In the CHEMO-INTENSITY randomization, reducing the dose of chemotherapy was hypothesized to provide a better patient experience without major detriment in PFS. The trial therefore used a PFS noninferiority design but with a relatively nonstringent boundary, set following careful discussion at a forum of patients and clinicians, where acceptable absolute PFS/OS losses were considered as a trade-off against toxicity. Patients were prepared to sacrifice 6 weeks or more of PFS in return for reduced treatment toxic effects, but clinicians were more conservative and the trial was eventually powered to exclude 34 days or greater reduction in median PFS from a predicted 134 days, equivalent to hazard ratio (HR) at or over 1.34. With 1-sided 5% significance and 80% power, this required 284 events or more per pairwise comparison, requiring recruitment of 501 patients or more. In the CHEMO-BSC randomization, chemotherapy was hypothesized to improve OS; however, given that the uptake of this randomization was not predicable at the time of designing the trial, the sample size could not be predetermined and only exploratory analysis was planned.

Efficacy analyses were by intention to treat (ITT); safety and toxic effect analyses in patients who received 1 or more dose of protocol therapy. Kaplan-Meier methods were used to estimate survivor functions for time-to-event end points.^23^ Cox proportional hazards regression adjusted for minimization factors were used to estimate HRs and CIs, using 1-sided 95% CIs for the noninferiority comparison.^24^ Proportional hazards were assessed using a permutation test of martingale residuals. No violations were observed. Overall Treatment Utility comparisons used ordinal logistic regression adjusting for minimization factors to estimate odds ratios (ORs) and 95% CIs.^25^

For QL subscales, we found no evidence against the missing-at-random assumption using descriptive and logistic regression analysis, so we applied multiple imputation by chained equations (MICE).^26^ We compared allocated groups for QL and symptom subscales using multiple linear regression adjusted for the baseline subscale and minimization factors (excluding center). For fatigue we compared groups using multilevel repeated mixed-model analyses allowing for time effects, treatment-time interactions, baseline fatigue (fixed effects), and patient and patient-time interaction (random effects). These models were also used to estimate treatment effects and 95% CI. We performed sensitivity analyses for complete cases.

Subgroup analysis used the same model methods as primary and secondary end point comparisons, prespecified for potentially prognostic variables: patient characteristics/minimization factors, frailty, QL/symptoms, and laboratory tests. Tests for heterogeneity correspond to 1 degree of freedom for 2-category subgroups (or continuous scales), 2 degrees of freedom for 3-category subgroups, and so on. Following univariate analyses with ordinal logistic regression, we performed multivariable analysis using backward elimination including all variables, irrespective of univariate result. Finally, we constructed a nomogram using a transformation of the linear predictor to a scale estimating the probability of each OTU outcome.

*P* values for superiority comparisons are 2-sided and considered significant at an overall significance level of 5%. All other analyses are described in the statistical analysis plan (Supplement 2). For analysis, SAS version 9.4 (SAS Institute) and R version 3.2.3 (R Project) were used.

## Results

**Figure 1.**
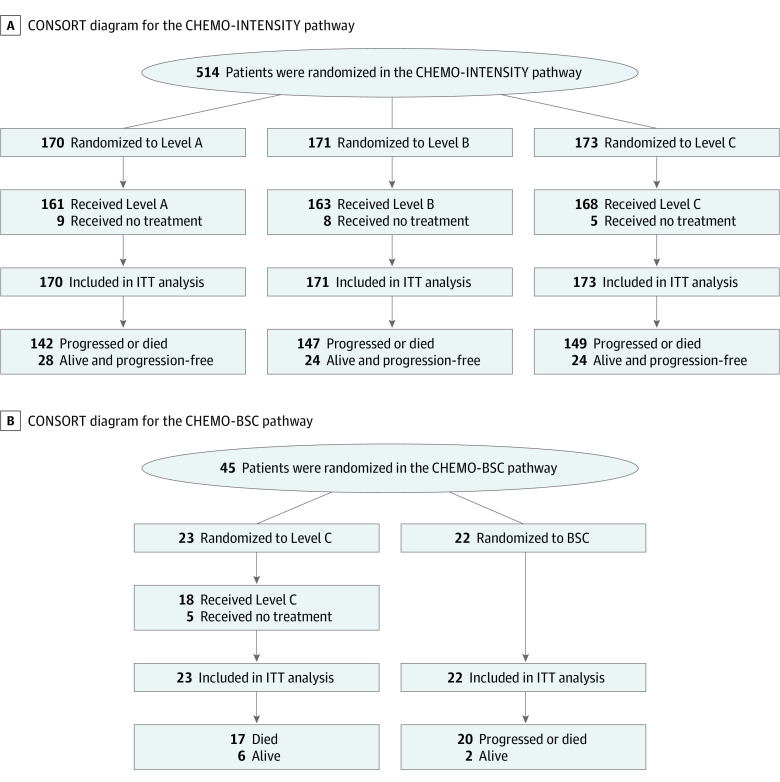
CONSORT Diagrams A, CONSORT diagram for the CHEMO-INTENSITY pathway. B, CONSORT diagram for the CHEMO-BSC pathway. Treatment pathways are detailed in the Randomization section of Methods. BSC indicates best supportive care alone; ITT, intention to treat.

A total of 514 patients entered the CHEMO-INTENSITY and 45 entered the CHEMO-BSC randomization, between January 2014 and November 2017, at 61 UK medical centers (Figure 1; eTable 1 in Supplement 3); cutoff date was February 2019. Slow recruitment in some centers was attributed to patients opting for the lower dose levels off-trial. Populations were well-balanced within each randomization but differed between the two, with CHEMO-BSC patients having higher rates of poor PS and severe frailty, driven especially by impaired ADL, low mood and social care requirements (Table 1; eTable 2 in Supplement 3). Of the total 559 patients, 44 (8%) were neither frail nor aged over 75 years, reflecting the flexibility of the selection criteria (eTable 3 in Supplement 3).

**Table 1.** Baseline Patient Characteristics

Treatment allocation	No. (%)				
	CHEMO-INTENSITY^a^ pathway			CHEMO-BSC^a^ pathway	
	Level A (n = 170)	Level B (n = 171)	Level C (n = 173)	Level C (n = 23)	BSC (n = 22)
Age, median (range), y	76 (57-96)	76 (51-91)	77 (56-88)	79 (66-89)	78.5 (58-88)
Male gender	131 (77)	129 (75)	125 (72)	14 (61)	13 (59)
WHO performance status					
0	27 (16)	23 (13)	22 (13)	0	0
1	90 (53)	94 (55)	95 (55)	9 (39)	6 (27)
2	49 (29)	47 (27)	52 (30)	11 (48)	14 (64)
>2	3 (1.8)	7 (4.1)	3 (1.7)	3 (13)	2 (9.1)
Frailty					
Not frail (0-1 domains)	23 (14)	30 (18)	41 (24)	2 (8.7)	1 (4.5)
Slightly frail (2 domains)	44 (26)	45 (26)	32 (18)	5 (22)	6 (27)
Severely frail (≥3 domains)	103 (61)	96 (56)	100 (58)	16 (70)	15 (69)
Frailty/age					
Age ≥75 y and frail	74 (44)	81 (47)	71 (41)	15 (65)	16 (73)
Age ≥75 y and nonfrail	16 (9)	15 (9)	20 (12)	1 (4)	1 (4)
Age <75 y and frail	73 (43)	60 (35)	61 (35)	6 (26)	5 (23)
Age <75 y and nonfrail	7 (4)	15 (9)	21 (12)	1 (4)	0
Squamous histology	20 (12)	18 (11)	20 (12)	4 (17)	5 (23)
Site of primary tumor					
Esophagus	55 (32)	73 (43)	69 (40)	13 (57)	9 (49)
GO junction	50 (29)	34 (20)	39 (23)	4 (17)	4 (18)
Gastric	64 (38)	64 (37)	64 (37)	6 (26)	9 (41)
Distant metastases	115 (68)	118 (69)	121 (70)	11 (48)	10 (46)
Trastuzumab use	7 (4.1)	10 (5.8)	10 (5.8)	0	0
Individual domains contributing to the Frailty Score^b^					
BMI<18.5	7 (4.1)	13 (7.6)	11 (6.4)	2 (8.7)	6 (27)
Weight loss	92 (54)	94 (55)	85 (49)	11 (48)	10 (45)
Mobility (TUGT)	103 (61)	91 (53)	95 (55)	19 (83)	14 (64)
Falls	8 (4.7)	9 (5.3)	7 (4.0)	2 (8.7)	0
Cognition	22 (13)	25 (15)	26 (15)	4 (17)	3 (14)
Function (ADL)	97 (57)	97 (57)	100 (58)	16 (70)	19 (86)
Social care	0	2 (1.2)	1 (0.6)	23 (100)	21 (95)
Mood	2 (1.2)	4 (2.3)	3 (1.7)	21 (91)	22 (100)
Fatigue	42 (25)	42 (25)	42 (24)	5 (22)	7 (32)
Polypharmacy	127 (75)	129 (75)	116 (67)	19 (83)	14 (64)

Abbreviations: ADL, activities of daily living; BMI, body mass index; WHO, World Health Organization; TUGT, Timed Up and Go Test. BMI is calculated as weight in kilograms divided by height in meters squared.

^a^ Treatment pathways are detailed in the Randomization section of Methods.

^b^ For frailty definitions, see the trial protocol (Supplement 2).

### CHEMO-INTENSITY Randomization

**Figure 2.**
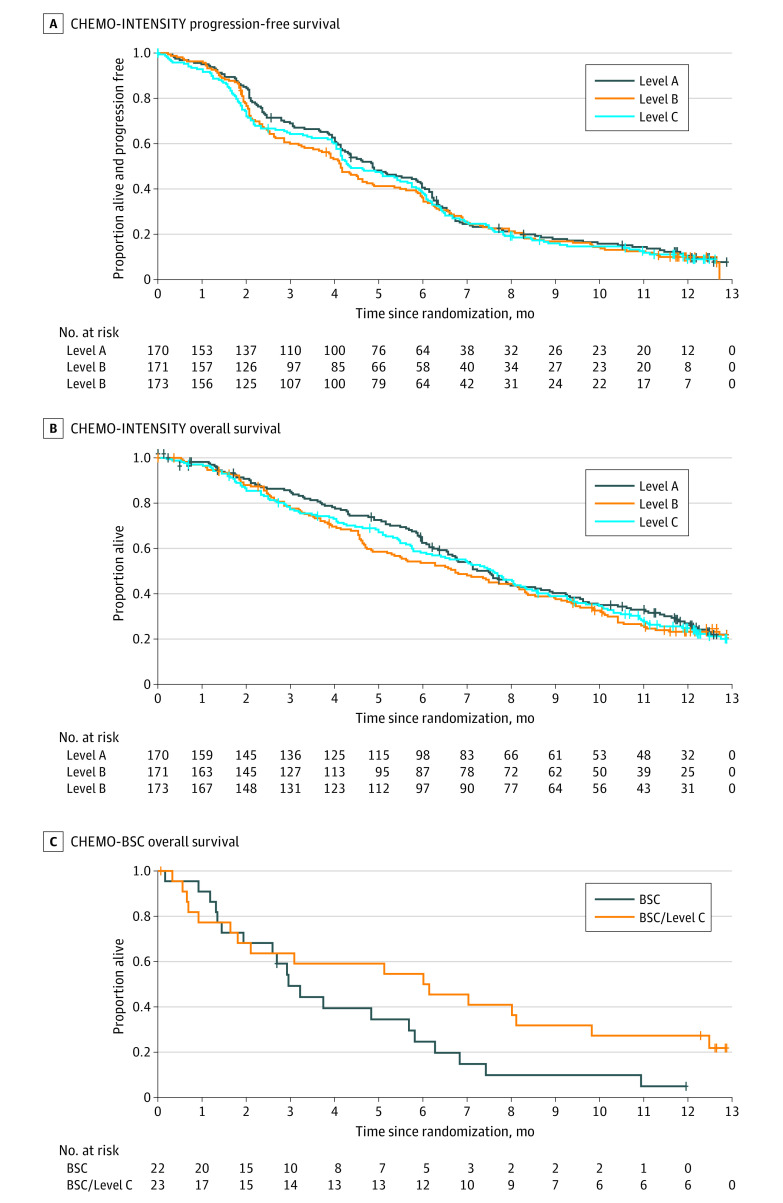
Survival Curves A, CHEMO-INTENSITY progression-free survival. B, CHEMO-INTENSITY overall survival. C, CHEMO-BSC overall survival. Treatment pathways are detailed in the Randomization section of Methods. BSC indicates best supportive care alone.

In the ITT analysis, both lower doses satisfied the prespecified trial definition of noninferior PFS compared with Level A; neither CI crosses the HR boundary of 1.34 (Figure 2). A total of 438 (85%) patients experienced PFS events, with HR = 1.09 (95% CI, 0.89-1.32) for B vs A and HR = 1.10 (95% CI, 0.90-1.33) for C vs A. After 373 (73%) deaths, there is no evidence that higher-dose treatment improved OS: B vs A HR = 1.09 (95% CI, 0.88-1.36); C vs A HR = 1.14 (95% CI, 0.92-1.41) (Figure 2). No subgroup was identified demonstrating clear benefit with Level A for either PFS or OS (eFigures 1-4 in Supplement 3). Among the 349 (68%) RECIST-assessable patients there was a lower response rate (CR/PR) in Level B, but not Level C (B vs A OR = 0.53 [90% CI, 0.33-0.85]; C vs A HR = 0.63 [90% CI, 0.36-1.11]), with little difference in disease control (CR/PR/SD) (eTable 11 in Supplement 3). Results for PFS were similar in the per-protocol analysis (n = 492), with HR = 1.09 (95% CI, 0.89-1.34) for B vs A and HR = 1.10 (95% CI, 0.90-1.34) for C vs A (eFigure 13 in Supplement 3).

Overall Treatment Utility, assessed in all 514 patients by ITT, was good in 196 patients (38%), intermediate in 149 (29%), and poor in 169 (33%). Level C produced more good (43%) and fewer poor (29%) OTU outcomes than A or B, but these differences were not statistically significant (eFigure 1 in Supplement 3). Other patient experience end points also trended toward better outcomes with lower doses (eTables 4-9 in Supplement 3). Time-to-deterioration of fatigue favored Level C (C vs A HR = 0.88 [95% CI, 0.65-1.19]). Global QL (EORTC Core Quality of Life questionnaire, QLQ-C30, and EuroQoL-5 Dimension, EQ-5D) improved between baseline and 9 weeks with Levels B and C, but not A (eFigures 4-7 in Supplement 3). Longitudinal fatigue and QL showed no major differences. Cancer symptoms improved between baseline and 9 weeks in all arms similarly (eFigures 8-12 in Supplement 3).

The rate of toxic effects fell markedly with reducing dose levels (Table 2). Consequently, treatment delivery was more reliable: during the first three 3-week cycles, comparing Levels A, B, and C, a dose reduction was required in 63 (39%), 39 (24%), and 21 (13%) patients, respectively; 53 (33%), 47 (29%), and 34 (20%) patients stopped treatment wholly or partly owing to toxicity, and 51 (32%), 72 (44%), and 97 (58%) patients respectively completed their first 3 cycles without reduction or stoppage (eTable 15 in Supplement 3). Mean (SD) treatment duration was 4.4 (3.3), 4.6 (4.0), and 5.4 (4.1) cycles, respectively, and 30 (18%), 36 (21%), and 47 (27%) went on to receive 6 or more cycles. Second-line therapy was recorded in 23 (14%), 18 (11%), and 24 (14%) patients.

**Table 2.** Toxic Effects Reported Within 9 Weeks of Starting Chemotherapy

Allocation	Randomization, No. (%)							
	CHEMO-INTENSITY^a^						CHEMO-BSC^a^	
	Level A (n = 162)		Level B (n = 162)		Level C (n = 168)		Level C (n = 18)	
Max CTCAE grade (week 1-9)^b^	≥2	≥3	≥2	≥3	≥2	≥3	≥2	≥3
Nausea or vomiting	47 (29)	14 (8.6)	33 (20)	8 (4.9)	29 (17)	12 (7.1)	2 (11)	0
Anorexia	45 (28)	11 (6.7)	46 (28)	14 (8.6)	32 (19)	3 (1.8)	13 (17)	0
Diarrhea	34 (21)	10 (6.2)	19 (12)	10 (6.2)	7 (4.2)	3 (1.8)	1 (5.6)	1 (5.6)
Peripheral neuropathy	24 (15)	4 (2.5)	11 (6.7)	1 (0.6)	6 (3.6)	1 (0.6)	2 (11)	0
Fatigue	86 (53)	24 (15)	72 (44)	20 (12)	67 (40)	18 (11)	6 (33)	4 (22)
Infection	7 (4.3)	4 (2.5)	15 (9.3)	9 (5.6)	5 (3.0)	1 (0.6)	0	0
Thrombosis	5 (3.1)	5 (3.1)	4 (2.5)	3 (1.9)	3 (1.8)	2 (1.2)	2 (11)	2 (11)
Any nonhematological^c^	125 (77)	62 (38)	116 (72)	58 (36)	101 (60)	38 (23)	10 (56)	7 (39)
WBC/neutrophils (×10^9^/l)	10 (6.2)	1 (0.6)	3 (1.9)	0	5 (3.0)	1 (0.6)	0	0
Anaemia	26 (16)	1 (0.6)	33 (20)	6 (3.7)	22 (13)	3 (1.8)	2 (11)	0
Any hematological^d^	33 (20)	3 (1.9)	36 (22)	6 (3.7)	27 (16)	4 (2.4)	2 (11)	0

Abbreviations: CTCAE, Common Terminology Criteria for Adverse Events; WBC, white blood cell count.

^a^ Treatment pathways are detailed in the Randomization section of Methods.

^b^ Maximum CTCAE grade experienced weeks 1-9 in patients receiving ≥1 cycle of their allocated chemotherapy. Individual listings are shown for more common toxic effects.

^c^ “Any nonhematological” is defined as any of the following: nausea, vomiting, anorexia, stomatitis, diarrhea, hand-foot syndrome, peripheral neuropathy, fatigue, infection, thrombosis, or dehydration.

^d^ “Any hematological” is defined as any of the following: low white blood cell count, low neutrophils/granulocytes, low platelets, or anemia.

### CHEMO-BSC Randomization

A total of 45 patients entered the CHEMO-BSC randomization. In those allocated chemotherapy, toxicity was higher than in patients allocated the same dose level in the CHEMO-INTENSITY randomization (Table 2). Longer OS was observed with chemotherapy than with BSC, but the difference was not statistically significant (HR = 0.69 [95% CI, 0.35-1.48]). Both QL and fatigue were nonsignificantly better with chemotherapy than BSC (eFigure 8 and eFigure 9 in Supplement 3).

### Baseline Predictors of OTU

Univariate analysis in all 537 patients allocated chemotherapy identified the following baseline factors associated with worse OTU (*P* ≤ .05): distant metastases, raised B-type natriuretic peptide (BNP) or N-terminal prohormone of brain natriuretic peptide (NT-proBNP), leukocytosis, raised neutrophil to lymphocyte ratio (NLR), hypoalbuminaemia, raised urea, severe frailty (dementia, activities of daily living [ADL], and polypharmacy domains), poor global QL, and impaired taste (eFigure 1 in Supplement 3).

In multivariable analysis, baseline frailty, EQ5D-VAS, and NLR were independently associated with OTU. These factors can be used to calculate a predictive score: (0.27 if not severely frail) + (0.39 if EQ-VAS ≥ 50) + (0.34 if NLR ≤ 4.0). This score (range 0-1) translates into the probability of good, intermediate, or poor OTU at 9 weeks (eTable 10 and eTable 11 in Supplement 3). Thus, a slightly frail patient with baseline EQ-VAS = 55 and NLR = 3.0 (predictive score = 1) has a 44% probability of good and 27% probability of poor OTU. Conversely, a severely frail patient with baseline EQ-VAS = 45 and NLR = 5.0 (score = 0) has only 18% probability of good OTU but 57% probability of poor OTU.

In the CHEMO-INTENSITY randomization (n = 514), interaction was seen between the multivariable predictive score and dose level (*P* = .01) with greater incremental benefit of lower-dose treatment in patients with better baseline scores: thus a patient with score = 1 allocated to Level C has 68% probability of good, 20% intermediate, and 12% poor OTU, but if the same patient is allocated Level A these probabilities are 41%, 30%, and 29%. No baseline score was identified as predicting better OTU with higher-dose treatment (eTables 12-14 in Supplement 3).

## Discussion

The GO2 randomized clinical trial is the first large trial testing the relationship between treatment intensity and patient-focused outcomes in frail and/or older patients with cancer. Previous reports have studied older patients who were fit enough to enter all-comer trials,^27^ or relied on traditional efficacy and safety end points.^28^ The GO2 trial uses modern methods and studies patients—the older frail, older nonfrail, and younger frail—who rarely participate in trials. In response to calls to address the deficit in evidence guiding treatment of vulnerable cancer patients,^29^ we offer GO2 as an exemplar of real-world, patient-centered research.

Lower-dose chemotherapy improved patients’ experience without compromising anticancer control. This balance is captured in OTU, an objective measure of a virtual conversation between physician and patient and reflecting their joint assessment of treatment value: “With the benefit of hindsight, am I glad I recommended this treatment?” and “Am I glad that I accepted it?” The GO2 trial also demonstrates that a baseline GA can contribute to the doctor-patient decision by estimating an individual’s probability of better or worse OTU.

In designing GO2, decisions were necessary for patient selection, treatment, and statistical design, all of which may be debated. For example, previous trials could not characterize patients who were not included, but it was precisely those patients who were to be selected for GO2. It was therefore necessary to use clinicians’ experience, rather than an objective tool, to offer trial entry to patients they assessed as unsuited to full-dose combination chemotherapy but able to receive reduced-intensity treatment.

Even the highest dose in GO2, Level A, was less-than-standard treatment, comprising just 2 drugs from the standard EOCap triplet.^2^ Although this includes full-dose oxaliplatin, it is combined with low-dose continuous capecitabine rather than the intensive intermittent schedule typically used in doublet therapy. Level C therefore represents just 60% of 2 out of 3 drugs, around 40% of full standard dose intensity. It is also important to recognize that Level A, although a reference schedule for this trial, is not standard therapy; indeed, the stimulus for the trial was a survey showing that there is no standard for this population.^7^ For this reason we did not apply the typical stringent noninferiority boundary demanded by regulators, but were instead able to work with patients and clinicians to carefully balance the competing needs for cancer control and good tolerability.

One mechanism for retaining cancer control despite lower doses is avoidance of toxicity-induced treatment reductions and stoppages. Toxic effects leading to treatment modification may be accepted by oncologists as part of standard oncology practice, but it represents a negative experience for patients and detracts from both quality of life and cancer control; and these impacts are particularly heightened in patients with poor baseline reserve. Only 32% patients starting Level A were able to receive 3 cycles without reduction or stoppage, compared with 58% with Level C.

The GO2 trial aimed to develop dose individualization guided by baseline geriatric assessment: we anticipated fitter patients would benefit from higher-dose treatment; however, we did not identify any group for whom the higher doses are preferable. Using the OTU outcome measure, reflecting the balance of benefits and harms, goes beyond conventional single-outcome models looking at survival or toxicity in isolation.^30^ In so doing, GO2 challenges a pervading assumption of oncology: that within the bounds of tolerability more is better. We hope it will stimulate research exploring lower-dose chemotherapy, perhaps extending to younger and less frail patients. We hope also that those designing trials of novel agents, including registration studies, will consider the option of lower-dose chemotherapy as the reference or platform to which novel agents are added, to widen access to these trials.

The 3-month survival benefit seen in the CHEMO-BSC randomization, though nonsignificant in isolation as a consequence of small numbers and an imbalance in patient characteristics, concurs with previous data^31^ and supports consideration of low-dose chemotherapy in vulnerable patients. This should, however, be interpreted alongside the baseline predictor, which helps identify patients at high risk of poor treatment utility, for whom BSC may be a preferable path.

### Limitations

A limitation of GO2 is that our GA was purely observational. Implementation of these findings—and future research—should embrace the newer concept of Comprehensive Geriatric Assessment (CGA): both identification of vulnerabilities and active remedial management to correct them. An outstanding research question is whether CGA-based pre-habilitation will convert a patient from low to high probability of achieving good OTU.

## Conclusion

The GO2 clinical trial shows that the goals of palliative chemotherapy in the older and/or frail population, including but not limited to cancer control, may be better achieved using treatment at doses well below those currently regarded as standard. Careful baseline geriatric health assessment in the oncology clinic can help predict the likelihood of achieving those goals, and so contribute to patients’ and clinicians’ treatment decisions. Assessing the outcome of cancer treatment should be multidimensional, including its value to patients and its adverse effects, and we recommend further development of OTU to capture this complexity. The GO2 trial offers a design paradigm for enhancing older patients’ access to research and ensuring that our evidence base embraces the whole population that we serve.

## Notes

Supplement 1. Trial Protocol

Supplement 2. Statistical Analysis Plan

Supplement 3. eTable 1: GO2 Study Sites and Principal Investigators eTable 2: Baseline characteristics eTable 3: Baseline Frailty domains eTable 4a: Summaries of missing data for quality of life questionnaires in CHEMO-INTENSITY pathway eTable 4b: Summaries of missing data for quality of life questionnaires in the CHEMO-BSC pathway eTable 5: EORTC-QLQ-C30 Global health status in the CHEMO-INTENSITY pathway eTable 6: EORTC-QLQ-C30 Functional scales in the CHEMO-INTENSITY pathway eTable 7: EORTC-QLQ-C30 Symptom scales in the CHEMO-INTENSITY pathway eTable 8: EORTC-QLQ-C30 Symptom scales - CHEMO-INTENSITY pathway eTable 9: EORTC-QLQ-C30 Fatigue scale in the CHEMO-INTENSITY pathway eTable 10: EORTC-OG-25 Symptom scales in the CHEMO-INTENSITY pathway eTable 11: Summaries of best response eTable 12: Factors Associated with OTU outcome eTable 13: Lookup table for multivariable prognostic model· eTable 14: Predictive ability of the multivariable prognostic model evaluated at each variable combination eTable 15: Chemotherapy delivery and tolerability (CHEMO-INTENSITY randomisation) eFigure 1: Influence of baseline factors and dose level on OTU eFigure 2: Univariate analysis: impact of baseline variables Overall Treatment Utility eFigure 3: Univariate analysis : Impact of baseline symptoms Overall Treatment Utility eFigure 4: Forest plot for CHEMO-INTENSITY PFS (Level A vs. Level B) eFigure 5: Forest plot for CHEMO-INTENSITY PFS (Level A vs. Level C) eFigure 6: Forest plot for CHEMO-INTENSITY OS (Level A vs. Level B) eFigure 7: Forest plot for CHEMO-INTENSITY OS (Level A vs. Level C) eFigure 8: EORTC-QLQ-C30 Fatigue scale in the CHEMO-INTENSITY pathway eFigure 9: EuroqoL EQ-5D-3L Score in the CHEMO-INTENSITY pathway eFigure 10: EORTC-QLQ-C30 Fatigue scale in the CHEMO-BSC pathway eFigure 11: EuroqoL EQ-5D-3L Score in the CHEMO-BSC pathway eFigure 12: Time to Deterioration of Fatigue in the CHEMO-INTENSITY Pathway eFigure 13: Per Protocol Population: PFS curves by randomised treatment arm

Supplement 4. Nonauthor Collaborators

Supplement 5. Data Sharing Statement
