# Supplementary material for: Efficacy of Reduced-Intensity Chemotherapy With Oxaliplatin and Capecitabine on Quality of Life and Cancer Control Among Older and Frail Patients With Advanced Gastroesophageal Cancer: The GO2 Phase 3 Randomized Clinical Trial
Source: JAMA Oncol. 2021 May 13;7(6):869–77. doi: 10.1001/jamaoncol.2021.0848 (PMC8120440; doi:10.1001/jamaoncol.2021.0848)
Supplement: Supplement 1. — Trial Protocol [file jamaoncol-e210848-s001.pdf]

GO2

Alternative chemotherapy for patients with advanced gastric or oesophageal cancer where standard 3-drug combinations are inappropriate

Protocol Version 7.0, 26th July 2018

Sponsor:	University of Leeds	
Supported by:	Cancer Research UK	
Chief Investigators:	Prof Matt Seymour	Dr Peter Hall
Address:	St James's Institute of Oncology<br>Level 4 Bexley Wing<br>St James's University Hospital<br>Leeds, LS9 7TF	Edinburgh Cancer Research Centre<br>University of Edinburgh<br>Western General Hospital<br>Crewe Road South<br>Edinburgh<br>EH4 2XU
Tel:	0113 206 7453	0131 777 3512
Email:	matt.seymour@nihr.ac.uk	p.s.hall@ed.ac.uk

EudraCT No: 2013-000009-21
ISRCTN44687907
REC No: 13/YH/0229
Sponsor's Protocol Code No: MO11/9969

Sponsor accepts no responsibility for the accuracy of content reproduced from this protocol and incorporated into additional documentation developed by collaborating or third party organisations

1 Key Contacts

General enquiries ctru-go2@leeds.ac.uk

Chief Investigators

Professor Matthew Seymour
Professor of Gastrointestinal Cancer Medicine, University of Leeds, St James's Institute of Oncology, Level 4, Bexley Wing, St James's University Hospital, Beckett Street, Leeds, LS9 7TF.
Tel: 0113 206 7453
matt.seymour@nihr.ac.uk

Dr Peter S Hall
Senior Lecturer / Honorary Consultant Medical Oncologist,
Edinburgh Cancer Research Centre, University of Edinburgh, Western General Hospital, Crewe Road South, Edinburgh, EH4 2XU
Tel: 0131 777 3512
p.s.hall@ed.ac.uk

Trial Management (for set-up, regulatory and process queries)

Sharon Ruddock, Senior Trial Coordinator
Clinical Trials Research Unit, Leeds Institute of Clinical Trials Research, University of Leeds, Leeds LS2 9JT.
Tel: 0113 343 7903
s.p.ruddock@leeds.ac.uk

Data Management (for eligibility and data queries)

EszterEszter Katona, Data Manager
Clinical Trials Research Unit, Leeds Institute of Clinical Trials Research, University of Leeds, Leeds. LS2 9JT.
Tel: 0113 343 1495
E.Katona@leeds.ac.uk

Statisticians

Helen Marshall, Principal Statistician
Clinical Trials Research Unit, Leeds Institute of Clinical Trials Research, University of Leeds, Leeds. LS2 9JT.
Tel: 0113 343 6959
h.c.marshall@leeds.ac.uk

Joanne Webster, Trial Statistician
Clinical Trials Research Unit, Leeds Institute of Clinical Trials Research, University of Leeds, Leeds. LS2 9JT.
Tel: 0113 343 9100
j.c.webster@leeds.ac.uk

CTRU Project Scientific Lead

Dr Fiona Collinson, Deputy Director (Cancer)
Clinical Trials Research Unit, Leeds Institute of Clinical Trials Research, University of Leeds, Leeds. LS2 9JT.
Tel: 0113 343 9126
f.j.collinson@leeds.ac.uk

Direct line for 24-hour randomisation
0113 343 8278

Fax number for consent forms and reporting SAEs and SUSARs

Project Delivery Lead

Helen Howard
Head of Trial Management - Solid Tumours
Clinical Trials Research Unit, Leeds Institute of Clinical Trials Research, University of Leeds, LS3 9JT

Tel: 0113 343 31485
H.C.Howard@leeds.ac.uk

Lead Pharmacist

Jo Askey
Advanced Clinical Pharmacist for Oncology & Onc/Haem Clinical Trials, St. James's Institute for Oncology, Bexley Wing, St. James's University Hospital, Beckett Street, Leeds, LS9 7TF.

Tel: 0113 206 8876
joanna.askey@nhs.net

University of Leeds Sponsor Contact

Clare Skinner
c/o University of Leeds / Leeds Teaching Hospitals NHS Trust, Research & Development, 34 Hyde Terrace, Leeds. LS9 6LN.

Tel: 0113 343 4897
c.e.skinner@leeds.ac.uk

Nurse Advisor

Anne Crossley, Senior Research Sister
St James's Institute of Oncology, Level 4, Bexley Wing, St James's University Hospital, Beckett Street, Leeds, LS9 7TF.

anne.crossley1@nhs.net

0113 343 6774

Clinical Co-Investigators

Dr Daniel Swinson (Oncology)
St James's Institute of Oncology, Level 4, Bexley Wing, St James's University Hospital, Beckett Street, Leeds, LS9 7TF.
daniel.swinson@nhs.net

Prof Russell Petty (Oncology and translational research)
Consultant Medical Oncologist
Division of Cancer Research
Medical Research Institute
University of Dundee
Mailbox 4,
Ninewells Hospital & Medical School
Dundee, DD1 9SY.
r.petty@dundee.ac.uk

Dr Heike Grabsch (Pathology and translational research)
Leeds Institute of Cancer Studies & Pathology, Wellcome Trust Brenner Building, St James's University Hospital, Beckett Street, Leeds, LS9 7TF.
h.i.grabsch@leeds.ac.uk

Prof Mike Bennett (Palliative Care)
Leeds Institute of Health Sciences, University of Leeds, Charles Thackrah Building Room G32, 101 Clarendon Road, Leeds, LS2 9LJ.
m.i.bennett@leeds.ac.uk

Prof Galina Velikova (Oncology and Psychosocial Oncology)
St James's Institute of Oncology, Level 4, Bexley Wing, St James's University Hospital, Beckett Street, Leeds, LS9 7TF.
g.velikova@leeds.ac.uk

Dr Justin Waters (Oncology)
Kent Oncology Centre, Maidstone Hospital, Hermitage Lane, Maidstone, ME16 9QQ.
justin.waters@nhs.net

Dr Simon Lord (Oncology)
Specialist Registrar in Medical Oncology and Clinical Research Fellow, University of Oxford and Oxford University Hospitals NHS Trust.
simon.lord@oncology.ox.ac.uk

Central laboratory contact details for translational research samples:

Grampian Biorepository
Central Laboratory: GO2 Trial
c/o Department of Pathology
Aberdeen Royal Infirmary
Link Building
Forresterhill
Aberdeen
AB25 2ZD
Scotland

Email: Grampian.Biorepository@nhs.net
Tel: 01224 550928

2 Contents

1 Key Contacts 2
2 Contents 5
3 Trial Summary 8
4 Glossary 10
5 Background 12
5.1 Current standard chemotherapy for GO cancer 12
5.2 Chemotherapy in elderly and/or frail co-morbid patients with GO cancer 13
5.3 Research in the frail/elderly in other gastrointestinal cancers – the FOCUS2 trial 14
5.4 321GO: a feasibility study in preparation for GO2 14
5.5 Aims of GO2 15
5.6 Defining the patient population 15
6 Aims and Objectives 16
6.1 Research questions for the chemotherapy intensity comparison 16
6.2 Research questions for the chemotherapy vs best supportive care comparison 16
6.3 Translational research 16
7 Design 17
7.1 Outcome measures for chemotherapy intensity comparison 17
7.2 Outcome measures for the chemotherapy vs BSC comparison 17
8 Eligibility 18
8.1 Inclusion criteria 18
8.2 Exclusion criteria 18
9 Recruitment Process 19
9.1 Recruitment setting 19
9.2 Eligibility screening 19
9.3 Informed consent and eligibility 19
9.4 Randomisation 20
9.4.1 Timing of randomisation 20
9.4.2 Treatment allocation 20
10 Trial Medicinal Product Management 22
10.1 Investigational Medicinal Products (IMPs) 22
11 Treatment & Management 22
11.1 Prescribing and compliance 22
11.2 Starting chemotherapy 23
11.3 Routine tests before each chemotherapy cycle 23
11.4 Treatment regimens 23
11.5 Management of toxicity - guidance on dose reductions/delays 24
11.6 Chemotherapy duration and breaks 25
11.7 Other anticancer treatment modalities 25
11.8 Best supportive care 25
11.9 Follow-up 26
11.10 Withdrawal of treatment 26
11.11 Concomitant medications 26
12 Assessments and Data Collection 27
12.1 Schedule of assessments 27
12.2 Imaging assessment schedule 28
12.3 Baseline assessments 28
12.4 Prior to every chemotherapy cycle (3-weekly) [OxCap arms only] 31
12.5 Nine weeks after randomisation [all trial patients] 31
12.6 Weeks 18, 27, 36 and 52 after randomisation [all trial patients] 32
12.7 Pregnancies 32
12.8 Deaths 32
12.9 Duration of follow-up 32
12.10 Definition of date of clinical disease progression 32

12.11 Definition of end of trial ... 33
12.12 Submission of trial data ... 33
12.13 Case report forms (CRFs) ... 33
12.14 Protocol deviations ... 33
13 Administration of participant questionnaires ... 33
13.1 Baseline questionnaire (all trial patients) ... 34
13.2 Limited Health Assessment (OxCap arm patients) ... 34
13.3 Short Follow-up Questionnaire (all trial patients) ... 35
14 Pharmacovigilance ... 35
14.1 General definitions ... 35
14.1.1 Adverse Event (AE) ... 35
14.1.2 Adverse Reaction (AR) ... 35
14.1.3 Serious Adverse Event (SAE) ... 35
14.1.4 Serious Adverse Reaction (SAR) ... 36
14.1.5 Suspected Unexpected Serious Adverse Reaction (SUSAR) ... 36
14.2 Reporting requirements for AEs and ARs ... 36
14.3 Recording and reporting SAEs and SUSARs ... 36
14.3.1 Events not to be classed as SAEs ... 36
14.3.2 Events classed as expected SAEs ... 37
14.3.3 Reporting and recording requirements for SAEs and SUSARs (BSC arm) ... 37
14.3.4 Reporting and recording requirements for SAEs and SUSARs (OxCap arms) ... 38
14.4 Responsibilities ... 38
15 Endpoints ... 40
15.1 Chemotherapy intensity comparison ... 40
15.2 Chemotherapy vs BSC comparison (exploratory) ... 40
15.3 Endpoint definitions ... 40
16 Statistical Considerations ... 41
16.1 Sample size ... 41
16.2 Quality of Life ... 44
16.3 Recruitment ... 44
16.4 Primary outcome based on chemotherapy vs BSC comparison (comparison B; exploratory): Overall survival ... 44
17 Statistical Analysis ... 46
17.1 General considerations ... 46
17.2 Comparisons ... 47
17.3 Chemotherapy intensity comparison ... 47
17.3.1 Primary endpoint: Progression-free survival (non-inferiority) ... 47
17.3.2 Secondary endpoints ... 48
17.3.3 Further analyses ... 50
17.4 Chemotherapy vs BSC comparison (exploratory) ... 51
17.4.1 Primary endpoint: Overall survival (superiority) ... 51
17.4.2 Secondary endpoints ... 51
18 Trial Monitoring ... 51
18.1 Trial steering committee and data monitoring and ethics committee ... 51
18.2 Data monitoring ... 51
18.3 Clinical governance issue ... 52
19 Quality Assurance and Ethical Considerations ... 52
19.1 Quality assurance ... 52
19.2 Serious breaches ... 52
19.3 Ethical considerations ... 52
20 Confidentiality ... 53
21 Archiving ... 53
22 Statement of Indemnity ... 53

23 Study Organisational Structure **54**
23.1 Individuals and individual organisations 54
23.2 Oversight and trial monitoring groups 54
24 Publication Policy **55**
25 References **56**
Appendix A – WHO Performance Status 58
Appendix B – Timed get up and go test 59
Appendix C – Capecitabine dose banding 60
Appendix D - Dose modifications 61
Appendix E – Response evaluation criteria in solid tumours (RECIST) 65
Appendix F – National Cancer Institute Common Terminology Criteria for Adverse Events (NCI-CTCAE) 65
Appendix G – Summaries of Product Characteristics 65
Appendix H – Overall Treatment Utility (OTU) Definition 66
Appendix I - Safety monitoring plan 67
Appendix J - Definition of frailty 69

3 Trial Summary

GO2 Trial Summary

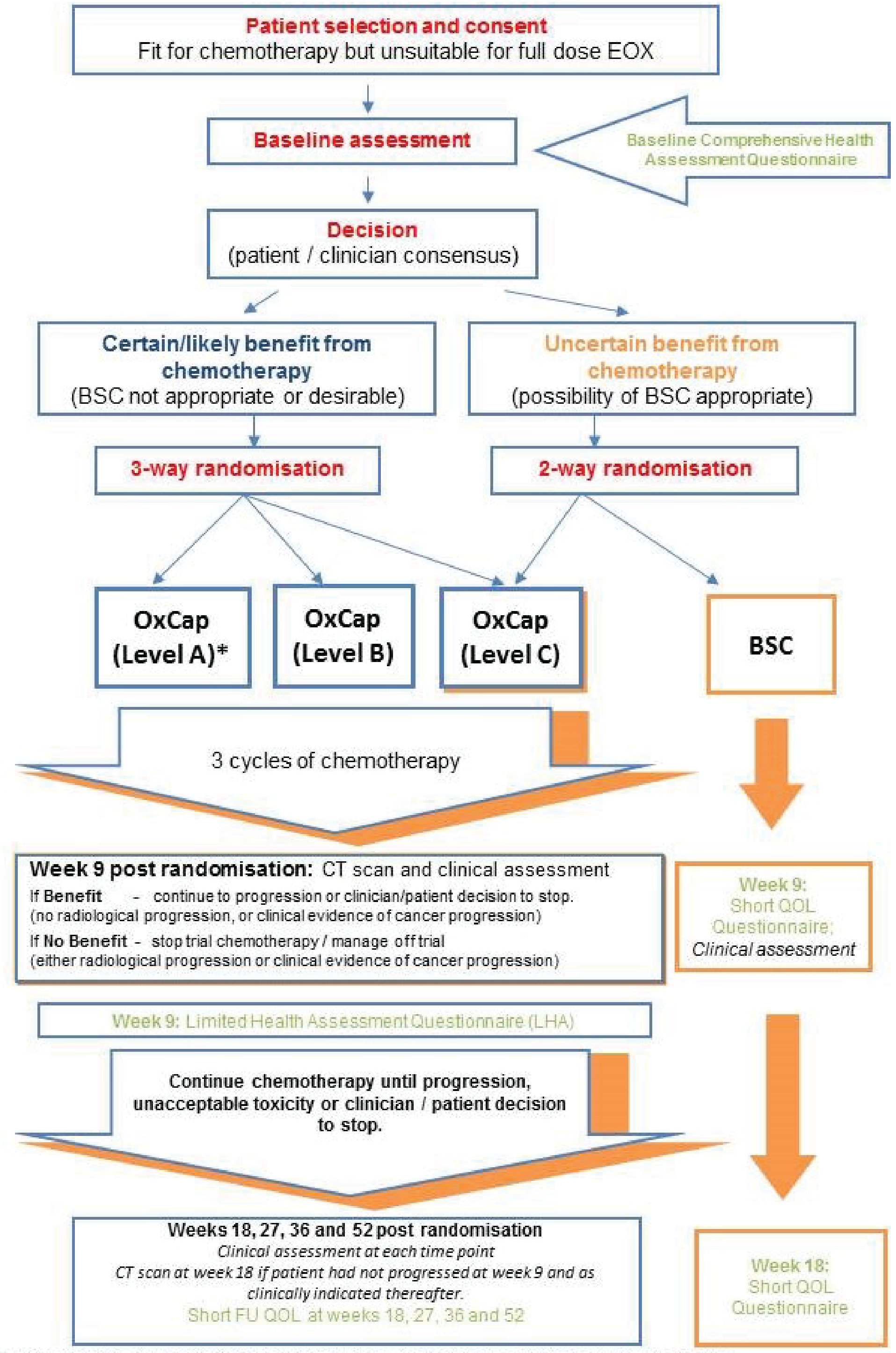

* Level A OxCap = Oxaliplatin 130mg/m² day 1; Capecitabine 625mg/m² bd x 21d as a 3 week cycle.
(This is the standard regimen used in the REAL2 trial, minus epirubicin.)

GO2 Trial Summary v2.0_20150129

**Sponsor**<br>University of Leeds	**Sponsor Protocol Number**<br>MO11/9969	**REC Number**<br>13/YH/0229
**EudraCT Number**<br>2013-000009-21	**CTA Number**<br>16767/0267/001-0001	**ISRCTN Number**<br>44687907

**Short Title**	**GO2**	
**Full Title**	Alternative chemotherapy for patients with advanced gastric or oesophageal cancer where standard 3-drug combinations are inappropriate	
**Chief Investigators**	Professor Matt Seymour and Dr Peter Hall	
**Population**	Patients with advanced gastric or oesophageal (GO) cancer, who are not fit for full-dose 3-drug chemotherapy, but suitable for reduced intensity chemotherapy	
**Trial Design**	Phase III, randomised, multi-centre, prospective, controlled, open label, non-inferiority trial	
**Aims**	• To establish the optimum dose-intensity of 2-drug palliative chemotherapy for advanced GO cancer to achieve the best balance of cancer control, toxicity, patient acceptability and quality of life for patients who are not considered suitable for 3-drug chemotherapy.<br>• To help establish pre-treatment patient characteristics in individual patients which predict for better or worse outcomes with chemotherapy at different dose intensities.	
**Research Questions**	1. Can lower-dose OxCap be used without significant compromise to disease control?<br>2. Does lower-dose OxCap provide advantages in terms of patient-assessed outcomes?<br>3. Does lower-dose OxCap offer advantages in terms of "Overall Treatment Utility" (OTU)?<br>4. Which aspects of the baseline health assessment can be used to predict OTU or other treatment outcomes?<br>5. Does chemotherapy improve overall survival, QoL or fatigue in patients for whom there is substantial uncertainty about the role of chemotherapy?	
**Endpoints**	**Chemotherapy intensity comparison**<br><br>**Primary endpoint**<br>• Progression-free survival<br>**Secondary endpoints**<br>• Participant reported fatigue<br>• Time to deterioration of participant reported fatigue<br>• Overall Treatment Utility<br>• QoL & symptoms<br>• Toxicity<br>• Overall survival<br>• Quality adjusted survival<br>• Best response	**Chemotherapy vs Best Supportive Care (BSC) comparison**<br><br>**Primary endpoint**<br>• Overall survival<br><br>**Secondary endpoints**<br>• Participant reported fatigue<br>• QoL
**Sample Size**	4-year recruitment period with no fixed sample size. Aim to recruit a minimum of 500 participants to the certain pathway, and an additional 60 participants to the uncertain pathway during this recruitment period.	
**Treatment duration**	Until cancer progression, unacceptable toxicity, or participant/clinician decision to stop.	
**Follow-up**	Follow-up visits for clinical and QoL assessment take place at 9, 18, 27, 36 and 52 weeks post randomisation.	
**Trial Management**	Clinical Trials Research Unit, University of Leeds<br>Contact: Sharon Ruddock, Senior Trial Coordinator, CTRU<br>Email: s.p.ruddock@leeds.ac.uk; Tel: 0113 343 7903	

4 Glossary

AE	Adverse Event
ALT	Alanine transaminase
AR	Adverse Reaction
AST	Aspartate aminotransferase
bd	bis in die (twice daily)
BNP	Brain natriuretic peptide
BSA	Body surface area
BSC	Best supportive care
Ca 19-9	Cancer Antigen 19-9 or Carbohydrate antigen 19-9
Cap	Capecitabine
CEA	Carcinoembryonic antigen
CHA	Comprehensive Health Assessment Questionnaire
CI	Chief Investigator or confidence interval
CONSORT	Consolidated Standards of Reporting Trials
CRF	Case Report Form
CRUK	Cancer Research UK
CT	Computerised tomography
CTA	Clinical Trial Authorisation
CTCAE	Common Terminology Criteria for Adverse Events
CTRU	Clinical Trials Research Unit
DMEC	Data Monitoring & Ethics Committee
DSUR	Development Safety Update Report
ECF	Epirubicin, cisplatin, fluorouracil
ECG	Electrocardiogram
ECX	Epirubicin, cisplatin, capecitabine
EDTA	Ethylenediaminetetraacetic acid
EOCap	Epirubicin, oxaliplatin, capecitabine
EOF	Epirubicin, oxaliplatin, fluorouracil
EOX	Epirubicin, oxaliplatin, capecitabine
EOxCap	Epirubicin, oxaliplatin, capecitabine
FBC	Full blood count
FFS	Failure-free survival
FQ	Follow-up Questionnaire
FSC	Feasibility Studies Committee
FU	Fluorouracil
GCP	Good Clinical Practice
GFR	Glomerular filtration rate
GO	Gastric /and oesophageal
HR	Hazard ratio
ICMJE	International Committee of Medical Journal Editors
ID	Identification
IMP	Investigational Medicinal Product
INR	International Normalised Ratio
IQR	Inter quartile range
ISRCTN	International Standard Randomised Controlled Trial Number
ITT	Intention to Treat

IV	Intravenous
LFTs	Liver function tests
LHA	Limited Health Assessment Questionnaire
MAR	Missing data at random
MCF	Mitomycin, cisplatin, fluorouracil
MDT	Multidisciplinary team
MedDRA	Medical Dictionary for Regulatory Activities
mg	Milligrams
MHRA	Medicines and Healthcare products Regulatory Authority
MNAR	Missing data not at random
MRC	Medical Research Council
NCRN	National Cancer Research Network
NT-proBNP	N-terminal pro-brain natriuretic peptide
O	Oxaliplatin
od	omne in die (once daily)
OS	Overall survival
OTU	Overall Treatment Utility
Ox	Oxaliplatin
OxCap	Oxaliplatin, capecitabine
OxFU	Oxaliplatin, fluorouracil
PFS	Progression-free survival
PI	Principal Investigator
PPI	Patient and public involvement
prn	pro re nata (as needed)
PS	Performance status
QAS	Quality adjusted survival
QoL	Quality of life
RCT	Randomised controlled trial
REC	Research Ethics Committee
RECIST	Response Evaluation Criteria in Solid Tumour
RGF	Research governance framework
SAE	Serious adverse event
SAR	Serious Adverse Reaction
SOP	Standard Operating Procedure
SPC	Summary of Product Characteristics
SUSAR	Suspected Unexpected Serious Adverse Reaction
tds	ter die sumendum (3 times a day)
TMG	Trial Management Group
TSC	Trial Steering Committee
U&Es	Urea and electrolytes
ULN	Upper limit of normal
WHO	World Health Organisation
X	Capecitabine

5 Background

Gastric and oesophageal (GO) cancer causes 13,000 deaths/year in the UK, at a median age of 77 years.[1] The peak age of diagnosis is becoming older,[2] and the diagnosis commonly follows a period of nutritional dysfunction. As a consequence, many GO cancer patients are frail, with co-morbidities and reduced performance status (PS).

[a] [b]

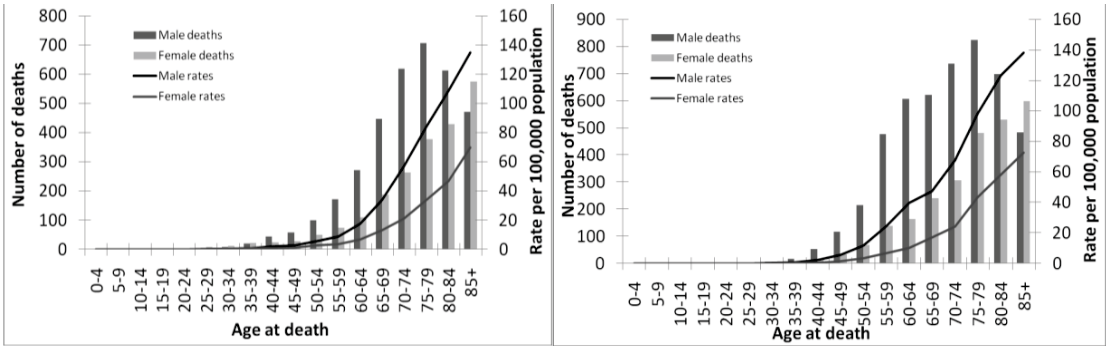

Fig 1. Age specific mortality: [a] stomach cancer, [b] oesophageal cancer

Recent years have seen a welcome shift in UK cancer management: all patients with malignancy, including the frail and elderly, are now managed by multidisciplinary teams (MDTs) with site-specialised oncology expertise. Consequently, most patients with advanced GO cancer are considered for, and many receive, chemotherapy as part of their palliative management. A report published by the Department of Health in conjunction with MacMillan and Age UK highlighted the lack of standardised care for older patients with cancer. It pointed to a need to identify appropriate methods for assessing patients for prognosis and their potential to benefit from evidence-based treatment.[3]

5.1 Current standard chemotherapy for GO cancer

Early trials of chemotherapy versus supportive care alone in GO cancer suggested around 6 months prolongation of median survival (from 3-5 months to 9-12 months), with quality of life benefits.[4] Over the past 15 years, a series of clinical trials in the UK and elsewhere established 3-drug regimens, typically fluoropyrimidine, platinum and anthracycline, such as “ECF” (epirubicin, cisplatin, fluorouracil).

Most recently, the NCRI trial, REAL2, has defined a new option for standard treatment.[5] REAL2 involved 1002 patients, median age 63 years, with 89% of good performance status (PS 0-1). ECF was the control and, in a 2x2 factorial design, FU was substituted with capecitabine (“X” or “Cap”), and cisplatin with oxaliplatin (“O” or “Ox”). In both cases, efficacy was maintained, and with the double-substitution “EOX” regimen, overall survival was significantly better than with ECF. Oxaliplatin and capecitabine also has some practical advantages, avoiding prolonged pre/post-cisplatin hydration and with no requirement for central venous access. Based on these results, the subsequent NCRI trial for fit patients with advanced GO cancer (REAL3) used EOX as its control arm, with patients randomised to receive this regimen +/- panitumumab.

The response rate to EOX in REAL2 was 48%. The individual contribution of each drug in the EOX regimen is difficult to evaluate. Phase II trials of single-agent capecitabine report response

rates of 24-34%,[6] and randomised phase II trials show that adding a platinum agent to fluorouracil improves efficacy.[7, 8] Of the three drugs in EOX, epirubicin is the least evidence-based. There is no unconfounded "+/- epirubicin" trial in GO cancer, but meta-analysis suggests that the contribution of epirubicin is modest,[9] and it contributes to toxicities such as alopecia, neutropenia and stomatitis.

5.2 Chemotherapy in elderly and/or frail co-morbid patients with GO cancer

Despite efforts to make the eligibility criteria for trials inclusive, there is a conspicuous mis-match between the age of patients with advanced GO cancer in the population (median over 75 years) and the populations recruited to randomised controlled trials (RCTs) such as REAL2 (median 63 years). There is a similar but less measurable mismatch in frailty, performance status and co-morbidity. This leaves uncertainty in both patient selection and choice of dose/regimen.

The only large randomised trial to have studied chemotherapy in an older patient population with GO cancer was the UK COMBAT trial conducted in the 1990s.[10] This tested the addition of mitomycin C to infusional 5FU, demonstrating no statistically significant advantage in the primary endpoint, failure-free survival (FFS). The median age was 73, 32% were performance status 2 and the median overall survival was 6.3 months. Six phase I/II studies have been conducted to look at chemotherapy in elderly patients with gastric cancer. Overall response rates ranged from 29% to 45%, with median progression-free survival (PFS) 4.2 – 5 months.[10-15] In addition, a pooled analysis of patients over 70 years of age in 3 phase III trials showed that this subgroup of patients had similar symptomatic response rates, objective response rates and survival, without increased toxicity compared to younger patients[16] This is consistent with the experience in colorectal cancer.[17] Generalisability from this study may be limited as patients included are likely to be more highly selected in comparison with many older patients treated in routine practice. A recent German trial also suggested improved tolerability of oxaliplatin over cisplatin in combination with infusional 5FU.[18] A follow-on study conducted specifically in elderly patients has demonstrated no advantage of a triple-drug regimen over an oxaliplatin-based doublet.[19]

It is now well recognized that age alone is no bar to benefit from chemotherapy. But age-related changes in pharmacokinetics and pharmacodynamics can lead to higher toxicity when elderly patients are treated with doses established in younger or fitter patients.[17, 20] Furthermore, the acceptability of complex treatments can be lower in this population.[21] A randomised controlled trial is now required for patients who are unfit for full-dose 3-drug chemotherapy, providing evidence to guide treatment. However, there was no consensus or guideline dictating best practice for less fit patients. There was therefore a need to define current real-world UK practice prior to conducting a phase III trial. Two studies were conducted to address this:

1. An audit of practice in a single large cancer centre. Patients over the age of 65 who were treated with palliative chemotherapy in Leeds for GO cancer between 2002 and 2006 were identified retrospectively using an electronic prescription database. 108 patients were identified. 32 (30%) were treated with full-dose 3-drug chemotherapy, 18 (17%) received reduced dose 3-drug chemotherapy, 12 (11%) received 2-drug regimens and 46 (43%) received a single agent fluoropyrimidine. 74 (69%) were over the age of 75 and of these only 2 received full dose 3-drug regimens.[22]

2. A national survey of oncologists treating GO cancer. Responses were obtained from 50 oncologists treating GO cancer across the UK in 2011. All but one respondent thought that

patients existed who were fit for chemotherapy but not full-dose EOX, and indicated that they would support a trial in this setting. 81% of oncologists used less-than-standard regimens in their practice. The choice of regimens was very diverse. The most common step was to drop the anthracycline. There was little enthusiasm for single agent therapy or carboplatin. Capecitabine was used much more frequently than 5FU. A full report is available from the GO2 trial management team.

5.3 Research in the frail/elderly in other gastrointestinal cancers – the FOCUS2 trial

Under-representation of elderly and frail patients in clinical trials has also been a problem in colorectal cancer. The Medical Research Council (MRC) FOCUS2 trial addressed this by specifically recruiting the elderly and/or frail. Treatments, which included single-agent capecitabine and oxaliplatin/capecitabine, were started at 80% of standard doses, with the option to escalate if no major toxicity had occurred.[23]

In FOCUS2, a rigorous 117 point “comprehensive health assessment” tool (CHA) was applied at baseline, with a “limited health assessment” (LHA) at 12 weeks and 24 weeks. It included the following domains: frailty, symptoms, nutritional status, medical comorbidity, cognitive function, mental health, activities of daily living and overall quality of life. Compliance was 100% at baseline, and 70% of available patients completed the 12 week assessment. Multivariable analysis including these factors alongside conventional prognostic factors has identified a number of baseline factors with potential predictive value for the benefit of palliative chemotherapy. A novel endpoint called Overall Treatment Utility (OTU) was developed to better reflect the balance of harm and benefit to patients. OTU is a composite of radiological & clinical response, toxicity & adverse events, and patient acceptability & valuation. OTU has been well received as a meaningful and pragmatic endpoint [24] and is being re-evaluated in GO2.

5.4 321GO: a feasibility study in preparation for GO2

Building on the success of FOCUS2, 321GO was undertaken with the support of the CRUK Feasibility Studies Committee (FSC).[25] Its objectives were to test the feasibility of randomising frail and elderly GO cancer patients into a RCT, and to assess the tolerability of 3, 2, and 1-drug reduced-dose chemotherapy regimens. 321GO took place across 2 NCRN research networks (2 tertiary centres and 4 district general hospitals). Eligible patients were those assessed to be unfit for full-dose triple agent chemotherapy (e.g. EOX, ECF) or ineligible for REAL3 on grounds of poor performance status, co-morbidity and frailty. Patients were randomised between EOCap, OxCap or Cap with all regimens starting at 80% of standard dose with the option to escalate to 100% after 2 cycles. The primary endpoint was recruitment rate and the acceptability of this randomisation to clinicians and patients.

Eligible patients made up a third of all referrals for consideration of palliative chemotherapy at participating MDTs. Recruitment (55 patients over an average of 18 months per centre over 2 NCRN networks) exceeded the pre-specified requirements for feasibility of a national study. EOCap was associated with greater toxicity compared with OxCap; Cap offered no improvement in tolerability over OxCap. Treatment benefit at 3 months (defined as no radiological progression or clinical deterioration) was seen in 47%, 58% and 16% of patients in the EOxCap, OxCap and Cap arms respectively. 321GO also provides an estimate of progression-free-survival (median 4.4 months) to facilitate power calculations for GO2.

5.5 Aims of GO2

GO2 aims to establish the optimum dose-intensity of 2-drug palliative chemotherapy for advanced GO cancer in patients who are not considered suitable for 3-drug chemotherapy, to achieve the best balance of cancer control, toxicity, patient acceptability and quality of life. It will also help establish pre-treatment patient characteristics in individual patients which predict for better or worse outcomes with chemotherapy at different dose intensities. Based on the results from 321GO, and the national survey of oncologists treating GO cancer, the 2 drug OxCap regimen has been taken forward in this phase III trial. In an exploratory analysis, GO2 also aims to determine whether chemotherapy improves overall survival in patients for whom there is substantial uncertainty about the role of chemotherapy.

5.6 Defining the patient population

There is no doubt that patients with GO cancer who are treated with less-than-standard chemotherapy consist of a heterogeneous group. It is possible to define the population to some extent using traditional prognostic parameters like age, performance status, comorbidity, extent of disease and biochemical measurements; but relying on these alone will ignore the subjective clinical assessment of the treating clinician. Allowing clinical judgement to define eligibility for GO2 in addition to objective measurements is critical for the accurate characterisation of the patient population and consequent translation of the results into clinical practice.

This presents two challenges in the design of GO2. Firstly, clinical judgement and thresholds for treating with either full-dose chemotherapy, less-than-standard chemotherapy or offering best supportive care vary between centres across the UK. This makes it very difficult to set objective eligibility criteria that accurately define the target population. Secondly, the potential acceptability of allocation to best supportive care will not be uniform across the population. This means that for some patients, less-than-standard chemotherapy is the strongly preferred option and a chance of allocation to best supportive care would make trial entry unacceptable or inappropriate. For other patients, who may be less fit or are treated in a centre which does not routinely use less-than-standard regimens, randomisation to best supportive care may be an equally acceptable option.

The GO2 trial therefore handles this problem by splitting the population into two groups, with each offered an alternative randomisation option. Patients who are certain or likely to benefit from chemotherapy are randomised to one of three chemotherapy dose intensities. Patients who are uncertain to benefit from chemotherapy are randomised to either the lowest of these three dose intensities or to best supportive care. The two groups are defined identically by objective eligibility criteria. The decision over which randomisation is appropriate for an individual patient is made by the treating clinician after discussion with the patient. The differences in the groups will therefore be defined by a combination of subjective clinical judgement, patient preferences and local routine practice. If further guidance is needed about how to arrive at a decision regarding the most suitable randomisation, please discuss with a member of the trial management team.

6 Aims and Objectives

6.1 Research questions for the chemotherapy intensity comparison

Comparison will be made between three dose-levels of OxCap chemotherapy in patients with advanced GO cancer who are not considered suitable for 3-drug chemotherapy, asking:

1. Can lower-dose OxCap be used without significant compromise to disease control?
   (***non-inferiority of progression-free survival*** **[PFS])**

2. Does lower-dose OxCap provide advantages in terms of patient-assessed outcomes?
   (***superiority of patient-reported EORTC fatigue, QoL (EQ-5D/QLQ-C30) & symptom scores***)

3. Does lower-dose OxCap offer advantages in terms of "Overall Treatment Utility" (OTU)?
   (***superiority of OTU)***
   [incorporating clinical & radiological control; patient-reported outcomes; toxicity & SAEs]

4. Which aspects of the baseline health assessment can be used to predict OTU or other treatment outcomes? (***regression analysis***)

6.2 Research questions for the chemotherapy vs best supportive care comparison

The lowest dose level of OxCap chemotherapy will be compared with best supportive care (BSC), asking:

1. Does chemotherapy improve overall survival in patients for whom there is substantial uncertainty about the role of chemotherapy?
   (***superiority for overall survival*** **[OS])**

2. Does chemotherapy improve quality of life and patient-reported fatigue in patients for whom there is substantial uncertainty about the role of chemotherapy?
   (***superiority for patient-reported EORTC fatigue & QoL (EQ-5D/QLQ-C30)***)

6.3 Translational research

The collection of blood and tumour biopsies from consenting trial participants will allow translational research to be undertaken. Translational research aims fall into 2 categories:

1. Biomarker research

Complementary to the aims of the therapeutic trial, biomarker research will aim to discover and qualify clinically useful predictive, therapy-independent prognostic, and treatment toxicity biomarkers. The comprehensive patient clinical assessments that will be undertaken in GO2 provides a unique opportunity to investigate the interaction between molecular biomarkers from biospecimens and the clinical tools, including an evaluation of their relative efficacy in outcome prediction. In addition the randomised BSC arm in the trial provides a rare opportunity to address and determine the predictive versus the therapy-independent prognostic impact of biomarkers.

2. Pathogenesis research
The linkage of the biospecimen collections to a detailed clinical and outcome database in GO2 will provide a very valuable resource for investigation of the molecular pathogenesis of gastroesophageal cancer in its own right, but will also form part of a larger collection in Leeds and Aberdeen of gastroesophageal biospecimen collections from clinical trials, which together provide a strongly powered, field leading resource in gastroesophageal cancer pathogenesis research.

7 Design

GO2 is a phase III, randomised, multi-centre, prospective, controlled, open label, non-inferiority trial comparing three dose levels of combination chemotherapy - oxaliplatin and capecitabine (OxCap). Level A is equivalent to the standard EOxCap regimen with the Epirubicin omitted; Levels B and C are equivalent to 80% and 60% of the doses used in Level A. Eligible patients are those not fit for full dose 3-drug chemotherapy, but suitable for reduced intensity chemotherapy. A second randomisation compares the lowest dose of OxCap with best supportive care in patients for whom there is substantial uncertainty about the suitability of chemotherapy. The trial is in the setting of the UK National Health Service.

7.1 Outcome measures for chemotherapy intensity comparison

Comparison:

OxCap Level A vs. OxCap Level B and OxCap Level A vs. OxCap Level C

Primary:

1.	Progression-free survival (PFS)	[Intention to Treat (ITT) population] and per protocol (PP) population]

Secondary:

1.	Participant-reported fatigue	[ITT population, PP population[1]]
2.	Time to deterioration of participant-reported fatigue	[ITT population, PP population[1]]
3.	Overall Treatment Utility (OTU)	[ITT population, PP population[1]]
4.	QoL and symptoms	[ITT population, PP population[1]]
5.	Toxicity	[Safety population]
6.	Overall survival (OS)	[ITT population, PP population[1]]
7.	Quality adjusted survival (QAS)	[ITT population, PP population[1]]
8.	Best response	[RECIST evaluable population]

7.2 Outcome measures for the chemotherapy vs BSC comparison

Comparison:

[1] Analysis of the primary endpoint (chemotherapy intensity comparison) will be performed on both the intention-to-treat (ITT) population and the per-protocol (PP) population. If a difference is seen between these analyses, the remaining endpoints (excluding best response and toxicity) will also be performed on both populations. If no difference is seen between the ITT and PP populations, the remaining endpoints will be performed on the ITT population only.

OxCap Level C vs. Best Supportive Care

Primary:

1. Overall survival [ITT population, PP population[1]]

Secondary

1. Participant-reported fatigue [ITT population, PP population[1]]
2. QoL [ITT population, PP population[1]]

8 Eligibility

Please check eligibility criteria carefully before approaching patients: waivers to inclusion and exclusion criteria are not permitted.

8.1 Inclusion criteria

Participants must meet all of the following inclusion criteria:

- Histologically or cytologically confirmed carcinoma of the oesophagus, GO-junction or stomach.[2]
- With or without distant metastases, but if M_0, being treated with palliative intent.
- Considered by the treating physician to be fit/suitable[3] for any of the GO2 regimens (or for the GO2 Level C regimen if entering the uncertain randomisation).
- Renal function: GFR ≥30 ml/min (estimated or measured).[4]
- Hepatic function: bilirubin <2 times upper limit of normal (xULN) and AST or ALT <5 times upper limit of normal (xULN).
- Bone marrow function: absolute neutrophil count ≥1.5 $x10^9$/l; white blood cell count ≥3 $x10^9$/l; platelets ≥100 $x10^9$/l.
- Written informed consent.
- Female participants of childbearing potential, or male participants who are sexually active with a female of childbearing potential, must be prepared to use contraception during chemotherapy and for 6 months after completion.

8.2 Exclusion criteria

Participants meeting any of the following exclusion criteria are not eligible:

- Fit, suitable (as judged by the treating clinician) and willing for standard full-dose combination chemotherapy with EOX or equivalent.
- Previous palliative chemotherapy for GO cancer.

---

[2] patients with endoscopic biopsy showing high grade dysplasia without definite invasion may be included if there is clear radiological evidence of extramural spread and the MDT diagnosis is invasive carcinoma

[3] Patients unable to swallow capecitabine tablets must be able to drink at least 200ml of fluid or have a feeding tube (see Section 11.4)

[4] For GFR in the range 30-50ml/min reduced doses of capecitabine are necessary as detailed in section 11.3 and Table D2 in Appendix D

- Medical or psychiatric condition impairing ability to consent or comply with oral chemotherapy or trial assessments (including patient-reported outcome measures).
- Other malignancy if, in the opinion of the treating physician, this would significantly impede interpretation of the outcomes of the trial treatment.
- Age <18 years.
- Pregnant or lactating.

9 Recruitment Process

9.1 Recruitment setting

Participants are recruited from NHS hospitals throughout the UK, through the NIHR CRN and devolved nations cancer research networks. Research centres must have obtained local management approval and been initiated by the CTRU before starting recruitment. Potential participants are identified principally though upper GI or gastroesophageal MDTs.

The trial aims to recruit a minimum of 500 participants to the 3-arm randomisation; there is no formal target for the 2-arm randomisation.

9.2 Eligibility screening

In order to determine the generalisability of the trial results, and for Consolidated Standards of Reporting Trials (CONSORT) requirements, sites are required to maintain a Screening Form logging all patients with advanced GO cancer who are considered unfit for full-dose 3-drug chemotherapy. This records the age, gender and whether or not the patient enters the GO2 trial.

For screened patients who are not recruited to GO2, anonymised data will be recorded for whether or not the patient was eligible for GO2. For patients who were not eligible, the reason for ineligibility is recorded; for patients who were eligible, the reason for the patient not entering the study is recorded. However, the right of the patient to refuse consent without giving reasons is respected. Screening forms should be returned to the CTRU on a regular basis.

9.3 Informed consent and eligibility

- Patients who are being invited to participate in GO2 first have a verbal explanation of the trial from the attending medical staff. In most cases this discussion focuses on **EITHER** the 3-way randomisation **OR** the 2-way randomisation, depending on which is felt by the clinician to be the more appropriate for the individual patient. After this verbal explanation, the patient is provided with the appropriate Patient Information Leaflet (PIL).
- For some patients, the clinician will feel it appropriate to discuss **BOTH** the 3-way **AND** the 2-way randomisation options. These patients may then require both the 3-way PIL and the 2-way PIL; an optional Introductory PIL is also provided to aid the choice of randomisation pathway. Together with the PIL(s), the patient is given a local contact point to obtain further information about the trial.
- Following the initial approach patients must be given as long as they need – a minimum of 24 hours – to consider whether to participate. They should be encouraged to discuss their decision with their family, carers and healthcare professionals during this time if they wish.
- Assenting patients are then formally assessed for eligibility and invited to provide informed, written consent. Confirmation of eligibility and written consent are countersigned by a clinician

who has signed the staff authorisation/delegation log. The right of the patient to decline without giving reasons is of course respected, as is the right to withdraw at any subsequent time without prejudicing their future care.

- A record of the consent process including the date and those present is made in the patient notes. The original consent form is retained in the investigator site file; a copy is given to the patient, a second copy filed in the hospital notes and a third copy returned to the University of Leeds CTRU.
- Informed consent must be obtained prior to investigations or other procedures specifically for the purposes of the study if they are not part of routine care at the participating site.
- The Principal Investigator (PI) has overall responsibility for the informed consent of participants at their site and for ensuring that any person delegated responsibility to participate in the informed consent process is authorised, trained and competent in accordance with the principles of Good Clinical Practice (GCP).
- In the event that a participant is required to re-consent, or new information is required to be provided to a participant, it is the responsibility of the PI to ensure this is done in a timely manner and according to any timelines requested by the CTRU.
- Where valid informed consent is obtained from the participant and the participant subsequently becomes unable to provide ongoing informed consent by virtue of physical or mental incapacity, the consent previously given when capable remains legally valid. Their protocol treatment and assessments may continue if the PI and participant's carer/family are agreed that this is in the participant's best interests.
- The responsibility for treatment with chemotherapy and the prescription of chemotherapy ultimately remains with the PI.
- After the patient has entered the trial, the clinician is free to give alternative treatment to that specified in the protocol if he/she feels it to be in the best interest of the patient. However, the reason for doing so should be recorded. The patient will remain within the trial for the purpose of follow-up and data analysis by Intent to Treat.

9.4 Randomisation

9.4.1 Timing of randomisation

Randomisation is performed by an authorised member of staff at the site, and can only occur after (a) eligibility has been confirmed, (b) written informed consent has been obtained, and (c) the baseline Quality of Life questionnaire has been completed,

Chemotherapy treatment should start treatment within 1 week after randomisation. Please ensure that this is feasible before randomising; if it is not, delay randomisation until within 1 week of the planned treatment start date and recheck the eligibility criteria at that time.

9.4.2 Treatment allocation

Randomisation is performed centrally using the CTRU automated 24-hour telephone system. Authorisation codes and PINs, provided by the CTRU, will be required to access the randomisation system. The following information will be required at randomisation:

- Research site name and site code
- Name of person making the randomisation
- Participant details, including initials, gender and date of birth
- Stratification factor details (see below)
- Confirmation of eligibility

- Confirmation of written informed consent and date
- Confirmation that the baseline questionnaire has been completed
- Decision pathway (certain/likely benefit or uncertain benefit from chemotherapy) – (see Section 5.6).

A computer generated minimisation program that incorporates a random element is used to ensure treatment groups are well-balanced for the following characteristics (details of these stratification factors are required for randomisation):

- Centre
- Age (≥75 or <75 years)
- Distant metastases (yes or no)
- Histology (squamous or other)
- Dose reduction required due to renal or hepatic function (yes or no) – (see section 11.3 and Table D2 in Appendix D)
- Planned use of trastuzumab (yes or no/not yet decided)
- WHO Performance status [Appendix A] (0-1 or 2 or >2).

Direct line for 24-hour randomisation

0113 343 8278

Please ensure that you have completed the Eligibility Checklist (F01), Nurse completed CHA (F02) and Randomisation (F03) CRFs, and the patient has completed the baseline QOL CHA questionnaire before telephoning

Participants entering the certain/likely benefit randomisation are randomised on a 1:1:1 basis to receive either:

1. Level A OxCap, or
2. Level B OxCap, or
3. Level C OxCap.

Participants entering the uncertain benefit randomisation are randomised on a 1:1 basis to receive either:

1. Level C OxCap, or
2. Best supportive care (BSC).

All randomised participants will be allocated a trial number.

After randomisation, the local hospital will provide each participant with a Trial ID card which they should carry with them at all times and present to medical staff should they be admitted to hospital during their time on trial.

10 Trial Medicinal Product Management

10.1 Investigational Medicinal Products (IMPs)

The IMPs used in this trial are:

- **Oxaliplatin**
  Composition: 5mg/ml concentrate for solution for infusion, or 5mg/ml powder for solution for infusion
  Supply: Generic off-the-shelf supply as determined by individual research sites. Please refer to the trial Summary of Product Characteristics (SPC) [Appendix G] and the SPC [Appendix G] for the product being used (if different), and ensure that all relevant SPCs are filed in the Investigator and Pharmacy Site Files. Pre-made bags are permitted.

- **Capecitabine**
  Composition: 150mg and 500mg film-coated tablets
  Supply: Generic off-the-shelf supply as determined by individual research sites. Please refer to the trial Summary of Product Characteristics (SPC) [Appendix G] and the SPC [Appendix G] for the product being used (if different), and ensure that all relevant SPCs are filed in the Investigator and Pharmacy Site Files.

All IMPs and other products used in this trial are commercially available; no special trial stocks are provided. Pharmacy is responsible for labelling IMPs in accordance with the requirements of Directive 2001/20/EC and the Medicines for Human Use (Clinical Trials) Regulations 2004 (and subsequent amendments).

The drug batch number should be added to a trial-specific prescription and/or recorded on the accountability logs, as per local practice. In the event of a safety issue or IMP quality issue, mechanisms to enable tracing back to a particular trial participant will be in place. Please refer to the GO2 Pharmacy and IMP Management Study Site Operating Procedure for full details of the trial IMP management requirements.

The guidelines in this protocol are in line with manufacturers' recommendations at the time of writing, but SPCs are updated from time to time. Up-to-date SPCs are posted on the Electronic Medicines Compendium website (http://emc.medicines.org.uk/) [Appendix G].

11 Treatment & Management

11.1 Prescribing and compliance

- Chemotherapy prescriptions should conform to local best practice including computerised prescribing systems.
- Capecitabine prescriptions to take home should include exactly the correct number of tablets for the current cycle.
  - Capecitabine prescriptions may be accompanied by a diary sheet, as per local practice, with baseline information completed by the pharmacist or research nurse, with instructions of how many tablets to take for each dose.
  - Special care is needed when dispensing doses of capecitabine that require the patient to take a combination of 500mg and 150mg tablets. It is important in this circumstance

that each box of tablets provided cross-references the need to take the other tablet size, and that nursing and/or pharmacy staff ensure that patients and their carers are fully aware of the intended dose to be taken.
    - Patients must be asked to return any unused tablets. Capecitabine compliance is reported on the case report form (CRF) after each cycle.
- The body surface area (BSA) should be determined using the preferred local method based on baseline weight and height. Recalculation of BSA for subsequent cycles is not mandated, but if there is a change in weight of more than 10% compared to baseline then recalculation should be considered.
- Dose capping is not used in this trial, regardless of the patient's BSA.[26]
- Female Patients of child-bearing potential, and male Patients who are sexually active with a female of child-bearing potential, should agree to use contraception for the duration of chemotherapy and for 6 months after stopping.

11.2 Starting chemotherapy

Chemotherapy treatment should start within 1 week after randomisation. Please ensure that this is feasible before randomising; if it is not, delay randomisation until within 1 week of the planned treatment start date and recheck the eligibility criteria at that time.

11.3 Routine tests before each chemotherapy cycle

FBC, biochemistry (including creatinine, bilirubin and either AST or ALT) and clinical assessment (CTCAE v4.0 toxicity scores – [Appendix F]) should be performed if possible the day before, and no more than 5 days prior, to each new chemotherapy cycle.

- Dose reductions in response to renal or hepatic impairment (GFR <50ml/min, bilirubin ≥1.5 xULN, AST or ALT more than doubled since baseline and > 2.5 xULN) as detailed in Table D2 are mandated for cycle 1 but local practice may be followed for cycle 2 and subsequent cycles.
- Delay 1 week if neutrophils $<1.0 \times 10^9$/l or platelets $<75 \times 10^9$/l. Only treat when neutrophils and platelets are above these limits.

11.4 Treatment regimens

In view of a potential in vitro chemical reaction between oxaliplatin and chloride ions, oxaliplatin mixing with saline must be avoided. Glucose 5% should be used both as the diluent for oxaliplatin infusion and for line-flushing before and after the oxaliplatin infusion.

Chemotherapy doses:

Trial arm (intensity)	Oxaliplatin<br>(on day one of a 21 day cycle)	Capecitabine<br>(twice daily on every day of a 21 day cycle)
Level A	130 mg/m^2	625 mg/m^2
Level B	104 mg/m^2	500 mg/m^2
Level C	78 mg/m^2	375 mg/m^2

Treatment schedule (21 day cycle)

Day 1	IV bolus dexamethasone 8mg + granisetron 1-3mg (or equivalent/local practice),<br>then oxaliplatin IV infusion in 500 ml 5% glucose (or local practice) over 2 hours
Days 1-22	capecitabine twice daily, oral.

Notes:

- The cycle is repeated every 21 days (+/- 3 days)
- Oxaliplatin may cause vein pain, which is helped by applying an electric heat pad over the vein throughout the 2-hour infusion.
- Oxaliplatin dose may be banded according to local practice. The system used should ensure that the delivered dose is within 10% of the mg/m2 calculated dose.
- The treatment cycle includes 42 capecitabine doses taken 12-hourly, starting on the evening of day 1.
- Capecitabine is prescribed at the same dose morning and evening, using 500 mg and/or 150mg tablets as per the table in Appendix C, or as per local standard practice. If a local system is to be used instead of Appendix C, please supply details to CTRU; the dose given must be within 100mg or 10% (whichever is larger) of the mg/m^2 calculated dose.
- Patients are instructed to take capecitabine within 30 minutes after food, approximately 12 hourly (e.g. 8 am and 8 pm).For patients who cannot swallowing capecitabine tablets, they may be dissolved by placing in approximately 200ml water and stirring for up to 15 mins. The solution may then be taken by mouth or via an enteral feeding tube. It has a bitter taste, and fruit juice can be added to make it more palatable, but grapefruit juice should not be used. It should be used immediately as the stability of the solution is not known.
- If a patient vomits after taking a dose of capecitabine, the dose should not be taken again.

Oral antiemetics, etc:

- From day 2: dexamethasone 4 mg tds x1 day; 4 mg bd x1 day; 4 mg od x1 day (or local practice).
- Domperidone or metoclopramide prn (or local practice).

Note on the use of dexamethasone

- For patients at high risk of steroid side effects (e.g. diabetics) or for those who develop toxicity attributable to steroids (e.g. dyspepsia; dysphoria; etc.), the oral steroid should be omitted or local practice should be followed.

11.5 Management of toxicity - guidance on dose reductions/delays

Appendix D contains guidelines for dose modification in response to organ function and toxicity. These should be adhered to wherever possible, however, it is acknowledged that trial investigators and treating consultants are likely to have significant experience with the trial drugs. Deviations from these guidelines and any dose reductions considered in the patients' best interest are therefore permitted and should be recorded in the Case Report Forms (CRFs) and will not

constitute a protocol violation. We recommend that if a dose-reduction is required for toxicity, the dose should not subsequently be re-escalated

11.6 Chemotherapy duration and breaks

- The initial treatment period is 9 weeks (3 cycles).
- The participant should be scheduled for a repeat CT scan and clinic visit at 9 weeks post randomisation (+/- 2 weeks).
- It is recommended this scan is booked in as soon as possible following randomisation. The 9 week participant questionnaire is also due at this time point and should be completed prior to discussion of the outcome of clinical investigations with the participant.
- On the basis of the radiological and clinical review, the clinician should decide whether the outcome of treatment has been:
    - **"benefit":** (= No radiological progression, and no clinical evidence of cancer progression), or
    - **"no benefit":** (= Radiological progression, or clinical evidence of cancer progression).
- Participants with **"benefit"** should continue treatment until cancer progression, unacceptable toxicity or clinician/participant agreement to stop.
- Participants with **"no benefit"** at this 9-week assessment should stop treatment or be considered for second-line therapy off trial.
- Treatment may be stopped at any time, at the discretion of the responsible consultant, if clear evidence of "no benefit" emerges between scheduled assessment points, or at the participant's request.
- Ideally theret should be no longer than 6 weeks between Day 1 of consecutive cycles. If a patient's treatment is delayed for toxicity but without evidence of disease progression, please resume treatment (with any dose reductions as required – see Appendix D) as soon as is clinically safe and reasonable, and preferably within 6 weeks of the preceding cycle Day 1. Radiological assessments should continue at the same times as if the chemotherapy had not been delayed.

11.7 Other anticancer treatment modalities

- If, in the opinion of the treating consultant, an alternative treatment modality becomes indicated at any stage, it may be offered (e.g., palliative radiotherapy, surgery or oesophageal stent). GO2 trial treatment may be continued after any such treatment.
- If trastuzumab is being considered, HER-2 testing should be arranged prior to randomisation, wherever possible.
- Participants for whom trastuzumab is indicated in accordance with local and national guidelines may receive trastuzumab concurrently with their allocated trial chemotherapy. This is considered to be a concomitant medication and method of administration is not specified by this protocol.

11.8 Best supportive care

In the 2-arm randomisation, participants randomised to receive best supportive care (BSC) are treated according to local policy. This should include availability, if indicated, of:

- Palliative radiotherapy
- Endoscopic stenting
- Palliative surgical procedures

- Specialist palliative care service
- Specialist pain service and analgesia
- Psychosocial support (e.g. counselling)
- Blood transfusions
- Nutritional support (e.g. dietary advice, artificial feeding).

At all times, management should be in the best interests of the participant. This may include systemic anti-cancer treatments should they become clearly indicated (these should be reported on the Case Report Forms).

11.9 Follow-up

- Once randomised, participants remain evaluable for all endpoints until 1 year from randomisation, regardless of their subsequent course and treatment.
- Follow-up data on all participants, including details of other treatments given, is important and will be collected until 1 year post randomisation. See Sections 12 and 13 for follow-up schedule and assessments.
- Survival data, including date of death, or last date known to be alive, will be collected for each participant approximately 1 year after randomisation of the final participant.

11.10 Withdrawal of treatment

In line with usual clinical care, cessation or alteration of regimens at any time is at the discretion of attending clinicians or the participants themselves. Participants who withdraw from the protocol treatment should still undergo follow-up assessments if they are willing to do so, and CRFs will continue to be completed.

If treatment is withdrawn due to disease progression or toxicity then this is not classed as a patient withdrawal and will be captured via the standard trial CRFs rather than the withdrawal form.

The PI or delegate should make every effort to ensure that the specific wishes of any participant who wishes to withdraw consent for further involvement in the trial are defined and documented using the Withdrawal CRF, in order that the correct processes are followed by the CTRU and site following the withdrawal of consent. **It should be made clear to any participant specifically withdrawing consent for further data collection that data pertaining to safety will continue to be collected for regulatory reporting purposes and will be included in any safety analysis. We suggest that the participant is also made aware that if significant new information becomes available about the treatment they have received in the trial it may be necessary to contact them in the future.**

11.11 Concomitant medications

- As for any patient embarking on palliative chemotherapy, it is good practice to review the need for non-cancer medications at the time of starting GO2 treatment.[27]
- The following medications may interact with GO2 medications. These medications are not contraindicated but should be avoided unless there is no reasonable alternative:
    - Warfarin: no interaction with oxaliplatin, but INR control may be affected by capecitabine. Consider increased frequency of INR monitoring, or change to low molecular weight heparin.
    - Phenytoin: blood phenytoin levels may increase with capecitabine. Consider checking therapeutic levels after 3-6 weeks of chemotherapy therapy.

- o Folic acid: multivitamin supplements containing folic acid should be avoided as this may increase capecitabine toxicity.
- o Allopurinol: may potentially reduce the effectiveness of capecitabine.

- Concomitant medications for side effects of chemotherapy, e.g. anti-diarrhoeals, anti-emetics, mouthcare or skin care may be given as per local practice.
-

12 Assessments and Data Collection

12.1 Schedule of assessments

The timing of assessments required for the GO2 trial are summarised in Table 1. Refer to Section 13 for timing of quality of life questionnaire administration.

Table 1. Schedule of assessments

	Baseline, pre-treatment		Post-randomisation			
	Pre-randomisation (OxCap and BSC arms)	Pre-Cycle 1 or BSC	Pre each cycle (OxCap arms only)	Week 9	Week 18	Weeks 27, 36 & 52
Clinical evaluation	X[1]		X[8]	X	X	X
Clinical history	X[1]					
WHO Performance Status	X[1]		X[8]	X	X	X
FBC, U&Es, LFTs	X[1]		X[8]			
GFR[2]	X[1]					
Brain Natriuretic Peptide (BNP) or NT-proBNP	X[3]					
ECG	X[3]					
Tumour markers (CEA and Ca 19-9)	X[3]			X (if raised at baseline)		
Pregnancy test (if woman of childbearing potential)	X as per local practice					
CT Scan (or equivalent)[11] – RECIST measurement [Appendix E]	X[10]			X OxCap arms only	X OxCap arms only	Whilst on chemotherapy and as clinically indicated
Timed get up and go test [Appendix B]	X					
Nurse-completed CHA assessments	X[4]					
Toxicity review CTCAE v4.0 [Appendix F]		X[5]	X[9]			
Reporting of serious adverse events (SAEs/SUSARs)		Monitor from randomisation until 30 days post cessation of IMP OxCap arms only				

**20 ml EDTA Blood Sample (only if participant has consented to this)**		**X[6]**				
**Send Diagnostic Pathology specimen to central laboratory (only if participant has consented to this)**		**X[7]**				
**RECIST response [** Appendix E**] + clinical benefit status**				**X** OxCap arms only	**X** OxCap arms only	**X** If scan performed
**Participant completed QoL questionnaires**	**See section 13**					

[1] within 3 weeks prior to randomisation

[2] Measured (e.g. isotopic) GFR not required for randomisation if the formula-derived GFR (using Wright formula or equivalent) is >30ml/min. If a formula-derived GFR is <50 ml/min, it is recommended that a GFR is measured by radioisotopic clearance or equivalent but local practice can be followed. If measured GFR is not available prior to cycle 1 then it is recommended to be arranged prior to cycle 2. The measured GFR should take precedence over the formula-derived GFR.

[3] within 3 weeks prior to, or 1 week after randomisation, prior to starting protocol treatment

[4] G8 screening tool, Instrumental activities of daily living (IADL), Social situation assessment

[5] within 3 weeks prior to starting protocol treatment

[6] chemotherapy arms: within 2 weeks prior to starting chemotherapy; BSC arm: within 2 weeks of randomisation

[7] pathology specimen is from diagnosis, but to be sent to the central laboratory at the same time as the EDTA blood sample (if consented to both)

[8] within 3 days prior to start of each cycle of chemotherapy

[9] within 3 days prior to start of each cycle of chemotherapy (cycle 2 onwards)

[10] within 4 weeks prior to starting protocol treatment

[11] imaging technique as per local standard practice

12.2 Imaging assessment schedule

All participants (including BSC arm) must have a baseline imaging assessment (usually CT scan) within 4 weeks prior to the start of protocol treatment). Imaging technique will be as per local standard practice. Imaging assessments will then be performed every 9 weeks (equivalent to 3 cycles of OxCap treatment) up to and including 18 weeks post randomisation for participants randomised to receive chemotherapy. Imaging should be performed as close as possible to 9 and 18 weeks post randomisation (no earlier than 7 and 16 weeks, and no later than 11 and 20 weeks after the start of chemotherapy, respectively). Thereafter, imaging is as clinically indicated, but whilst participants remain on chemotherapy, repeat imaging assessments at weeks 27, 36 and 52 are recommended.

12.3 Baseline assessments

Within 3 weeks prior to randomisation (existing assessments from before trial consent may be used if within the time specifications):

- History and examination.
- Assessment of WHO performance status [Appendix A].

- Full blood count and biochemistry. Calculate GFR using Wright formula or equivalent. If the formula-derived estimate is <50 ml/min, a measured GFR is recommended (e.g. by isotopic clearance). (The measured GFR is not required prior to randomisation or the commencement of chemotherapy but should be arranged prior to the second cycle).
- Ensure that a baseline CT scan (or equivalent) has been (or will be) performed **within 4 weeks prior to starting protocol treatment.**
- Tumour markers (CEA and Ca 19-9), Brain Natriuretic Peptide (BNP) or NT-proBNP and ECG, **within 3 weeks prior to, or 1 week after randomisation, prior to starting protocol treatment.**
- Pregnancy test (if woman of child bearing potential) as per local practice.
- Check all other inclusion and exclusion criteria in protocol section 8.

After written informed consent:

- **Prior to Randomisation:**
  - Perform a ‘Timed get up and go’ test [Appendix B].
  - Perform **Comprehensive Health Assessment (CHA).** NB this will take 30-60 mins so may require a separate visit.
  - Administer baseline quality of life questionnaire to the participant (see section 13).

- **Prior to Cycle 1 or BSC:**
  - Assess baseline toxicity scores (CTCAE v4.0 [Appendix F]).
  - If the participant has consented for the **blood sample**, please:
    - Collect the 20ml EDTA sample within 2 weeks prior to starting chemotherapy (OxCap arms), or within 2 weeks of randomisation (BSC arm). The 20ml blood sample is to be collected using EDTA tubes available at the site. There is no preference to the size of tubes used as long as the volume of the blood sample is at least 20 ml.
    - Invert each tube at least 5 times to mix blood and anticoagulant.
    - Anonymise it with the centre number, participant’s trial number and participant initials.
    - Complete the appropriate section of the **Blood sample CRF** and send the CRF to the Grampian Biorepository central laboratory **with** the sample using the packaging supplied to your centre at the time of centre set up.
    - A copy of the Blood Sample CRF should be retained in the patient file as confirmation that the blood sample has been sent.
    - Wherever possible blood samples should be despatched within 24 hours to the central laboratory. If this is not possible, samples should be refrigerated until the earliest next available postage slot.

  - If the participant has consented to provision of GO **tumour biopsy material**, please:
    - Retrieve at least 1 Paraffin-embedded tissue block, ideally from a tumour resection, although any block containing tumour is acceptable.
    - Anonymise it with the centre number, participant’s trial number and participant initials.
    - Send it to the central laboratory at the address below.
    - Complete the appropriate section of the **Tumour Sample CRF** and return the CRF with the sample to the Grampian Biorepository central laboratory using the using the packaging supplied to your centre at the time of centre set up. A copy of the form should be retained in the patient file as confirmation that the tumour sample has been sent.
    - The samples should be placed in the tube holder (sellotaped closed if necessary), within the sealable bag then into the mailing envelope.
    - There is no postage paid on the envelopes. Sites will be re-imbursed on receipt of invoice addressed to the Biorepository Manager sent to the address below. Invoices should be submitted on a 6-monthly basis or sooner if the total postage costs accumulated have reached £30.

- If the participant has consented to the provision of both blood sample and tumour biopsy material, wherever possible, send the samples together in the packaging provided.
- **Do not sent any samples to the Clinical Trials Research Unit (CTRU).**

Central Laboratory address:
Grampian Biorepository
Central Laboratory: GO2 Trial
c/o Department of Pathology
Aberdeen Royal Infirmary
Link Building
Foresterhill
Aberdeen
AB25 2ZN

It is the responsibility of the trial site to ensure that samples are appropriately labelled in accordance with the trial procedures to conform with the Data Protection Act 2018. Biological samples collected from participants as part of this study will be transported, stored, accessed and processed in accordance with national legislation relating to the use and storage of human tissue for research purposes and such activities shall at least meet the requirements as set out in the 2004 Human Tissue Act.

Samples will be accessible to members of the Trial Management Group, and also other researchers outside that group. Sample and data requests will be made via the Chief Investigator who will discuss proposals with the Trial Management Group and Trial Steering Committee. A data transfer agreement will be set up for each group requesting access to the data who have been approved by the TSC/TMG.

12.4 Prior to every chemotherapy cycle (3-weekly) [OxCap arms only]

Assessment prior to the start of a chemotherapy cycle should, if possible, be the day before, at most within 5 days prior to chemotherapy administration (local practice may be followed).

- Clinical evaluation, to include assessment for evidence of disease progression, toxicity scores (CTCAE v4.0 [Appendix F]) from previous cycle (cycle 2 onwards), and current WHO PS.
- Check FBC, U&Es, and LFTs.
- Record capecitabine compliance from previous cycle using tablet returns.
- This data should be collated on the **Treatment Form** which is returned to the CTRU after the administration of every cycle (i.e., every 3 weeks) and after the last cycle.

12.5 Nine weeks after randomisation [all trial patients]

- Clinical assessment for disease progression.
- Assessment of WHO performance status [Appendix A].
- Reassessment of tumour markers, if raised at baseline.

In addition,

For OxCap arms:

- Reassessment imaging (as baseline modality, usually CT) should take place as close as possible to 9 weeks from randomisation, but in time to allow the results to be available prior to the clinical assessment and decision to proceed with cycle 4 (see section 12.2).
- Administer the Limited Health assessment (LHA) participant questionnaire at the 9 weeks assessment visit (no earlier than 7 weeks after the start of chemotherapy). (see section 13).

For BSC arm:

- Administer the Short follow-up questionnaire to the participant (see section 13).

12.6 Weeks 18, 27, 36 and 52 after randomisation [all trial patients]

- Clinical assessment for disease progression (if not previously documented as progressed).
- Assessment of WHO performance status [Appendix A].
- Administer the Short follow-up questionnaire to the participant (see section 13).

In addition,

For OxCap arms:

- If the patient has not progressed at week 9 then imaging (as baseline modality, usually CT) should take place at 18 weeks (as close as possible, but no earlier than 16 and no later than 20 weeks) from randomisation, and thereafter as clinically indicated. Whilst the participant remains on chemotherapy, repeat imaging every 9 weeks (i.e. at 27 and 36 weeks), and 52 weeks is recommended. See section 12.2.

12.7 Pregnancies

All pregnancies and suspected pregnancies must be recorded on the Pregnancy CRF and sent to CTRU within 7 days of the site research team becoming aware of any pregnancies.

12.8 Deaths

All deaths must be recorded on the Notification of Death CRF and sent to the CTRU within 7 days of the site research team becoming aware of the death.

12.9 Duration of follow-up

All participants will be followed up via trial visits and CRF completion until 1 year post randomisation. CTRU will also obtain extended survival data from sites for all randomised participants approximately 1 year after randomisation of the final participant. Extended survival data will include date of death, or last date known to be alive. The only exceptions to this are those participants who withdraw consent for the collection of follow-up data.

12.10 Definition of date of clinical disease progression

The date of clinical progression is defined as the **date of the clinical assessment** at which progression is identified.

12.11 Definition of end of trial

The end of trial is defined as the date of the last participant's last data item, or the date when the last survivalsurvival data item is received.

12.12 Submission of trial data

Participating sites will be expected to maintain a file of essential trial documentation (Investigator Site File), which will be provided by the CTRU, and keep copies of all completed CRFs for the trial.

12.13 Case report forms (CRFs)

Data will be recorded by trial site research staff on CRFs and submitted by post to the GO2 trial team at the CTRU, University of Leeds. Consent forms will be faxed to CTRU. Only the participant's trial number plus date of birth and initials will be added to the CRFs (with the exception of the consent form). **Trial sites are responsible for obliterating all other personal identifiable data prior to sending CRFs and any other reports to the CTRU.** Following receipt, the CTRU will contact trial sites to resolve any missing or discrepant data.

A participant may withdraw consent for further follow-up information to be collected from their medical records (the only exception being any applicable adverse events experienced within the 30 days after protocol treatment has stopped which are needed to comply with regulatory requirements). However, any outstanding data applicable to time points prior to withdrawal will continue to be requested from the trial site until it is received by CTRU, and all information collected prior to the date of withdrawal will be included in the trial analyses.

It is the responsibility of each trial site to retain copies of all completed CRFs and to maintain their file of essential trial documentation (Investigator Site File), which will be provided by the CTRU, on site during the trial and then at their designated archive facility.

12.14 Protocol deviations

The CTRU undertake to adopt all reasonable measures to record data in accordance with the protocol. Under practical working conditions, however, some minor variations may occur due to circumstances beyond the control of the CTRU. All such deviations will be documented on the study records, together with the reason for their occurrence; where appropriate, deviations will be detailed in the published report.

13 Administration of participant questionnaires

Table 2. Schedule of questionnaire administration for participants randomised to the chemotherapy arms (Level A, Level B or Level C OxCap) and Best Supportive Care (BSC) arm

For all participants, the administration of the CHA, LHA, and Short Follow Up questionnaires is timed relative to randomisation, as follows:

Questionnaire	Administration method	Pre randomisation	Week (post randomisation)				
			9	18	27	36	52
Baseline CHA	Clinic	x[1, 2]					
Week 9 LHA	Clinic or post		x[1]				
Short Follow-up	Clinic, phone or post		x[2]	x[1, 2]	x[1]	x[1]	x[1]

[1]OxCap arm.
[2]Best Supportive Care (BSC) arm.

13.1 Baseline questionnaire (all trial patients)

- The baseline CHA questionnaire is administered in the outpatient clinic by the research nurse after written informed consent has been obtained.
- **The baseline questionnaire must be completed by the participant before randomisation (it is a requirement in order to proceed with randomisation).** The baseline questionnaire may be completed at the same visit as consent and randomisation, but must be within 4 weeks prior to starting protocol treatment.
- If possible, all questionnaires should be completed prior to the participant's clinical consultation as this aids objectivity and compliance.
- The research nurse should be available to assist (e.g. with reading questions or marking responses) if the participant requires – this is essential given the frail or elderly nature of the participants in GO2.
- The research nurse should also check through the questionnaire after completion and ensure that all questions have been answered, and that the participant ID and date are completed.
- Please note that, once randomised, all participants remain in the trial and the follow-up questionnaires are required even if participants do not complete protocol treatment.

13.2 Limited Health Assessment (OxCap arm patients)

- The Week 9 questionnaire is administered in the outpatient clinic by the research nurse. If the participant is not attending clinic at the required timepoint, the questionnaire should be posted to the participant at home in time to be completed on the due date.
- If possible, all questionnaires should be completed prior to the participant's clinical consultation (if possible), as this aids objectivity and compliance.
- The research nurse should be available to assist (e.g. with reading questions or marking responses) if the participant requires – this is essential given the frail or elderly nature of the participants in GO2.

- The research nurse should also check through the questionnaire after completion and ensure that all questions have been answered, and that the participant ID and date are completed.
- The Week 9 questionnaire should be completed as close as possible to 9 weeks from randomisation and no earlier than 7 weeks after the start of chemotherapy. If completion at 9 weeks is not possible then please complete it as soon as possible after this time-point (for example, if there has been a delay to a chemotherapy cycle then it may be completed at the pre-cycle 4 clinical assessment).
- Please note that, once randomised, all participants remain in the trial and the follow-up questionnaires are required even if participants do not complete protocol treatment.

13.3 Short Follow-up Questionnaire (all trial patients)

- For patients receiving chemotherapy, the Short Follow Up questionnaire is administered at the patients follow up visits at weeks 18, 27, 36 and 52
- Participants randomised to Best Supportive Care complete the short follow-up questionnaire at weeks 9 and 18 post randomisation.
- The short follow-up questionnaire captures health status and contains eight questions about quality of life and fatigue and a visual-analogue scale.
- The short follow-up questionnaire is preferably completed in clinic. If the participant is not attending clinic at the required timepoint, the questionnaire should either be administered to the participant by the research nurse over the telephone or posted to the participant at home in time to be completed on the due date.
- Please note that, once randomised, all participants remain in the trial and the short follow-up questionnaires are required even if participants do not complete protocol treatment.

14 Pharmacovigilance

14.1 General definitions

14.1.1 Adverse Event (AE)

Any untoward medical occurrence in a patient or clinical trial subject administered a medicinal product and which does not necessarily have a causal relationship with this treatment.

14.1.2 Adverse Reaction (AR)

All untoward and unintended responses to an investigational medicinal product related to any dose administered. This definition implies a reasonable possibility of a causal relationship which is supported by facts, evidence or arguments to suggest a causal relationship. This definition includes medication errors and uses outside what is foreseen in the protocol (i.e. if an AR occurs as a result of a medication error).

14.1.3 Serious Adverse Event (SAE)

Any untoward medical occurrence or effect that:

- results in death.

  Death may occur as a result of the basic disease process. Nevertheless, all deaths occurring within 30 days of the last administration of the study agent, whether deemed to be related to treatment or not, must be treated as an SAE

and reported as such. All deaths which may be considered as related to the trial agent, regardless of the interval, must be treated as a SAE and reported as such.

- is life-threatening.
  The term life-threatening refers to an event in which the participant was at risk of death at the time of the event; it does not refer to an event which hypothetically might have caused death if it was more severe.
- requires inpatient hospitalisation or prolongation of existing hospitalisation.
- results in persistent or significant disability or incapacity.
- consists of a congenital anomaly or birth defect.
- jeopardised the participant or required intervention to prevent one of the above.
- is otherwise considered medically significant by the Investigator.

Medical and scientific judgement must be exercised in deciding whether an event is serious in other situations. These characteristics / consequences must be considered at the time of the event and do not refer to an event which hypothetically may have caused one of the above.

14.1.4 Serious Adverse Reaction (SAR)
An SAE deemed to have been related to an IMP used within the trial. Any suspected transmission via a medicinal product of an infectious agent is also considered a serious adverse reaction.

14.1.5 Suspected Unexpected Serious Adverse Reaction (SUSAR)
A Suspected Unexpected Serious Adverse Reaction (SUSAR) is a serious adverse drug reaction that is deemed to have been related to one of the trial IMPs, but, the nature or severity of which, is not consistent with the known toxicity profile. When determining whether a SAR is expected or not, please refer to the Reference Safety Information contained in Section 4.8 of the version of the Summary of Product Characteristics supplied in the Investigator Site File, or the latest updated version as instructed by CTRU.

14.2 Reporting requirements for AEs and ARs
Non-serious Adverse Events (AEs) which have no causal relationship with an IMP will not be collected in this trial, but must still be recorded in the participant's medical notes.

Adverse reactions (ARs) will be recorded in the appropriate CRFs from the commencement of oxaliplatin or capecitabine until 30 days after OxCap is **permanently** ceased. ARs will **<u>not</u>** be collected in this trial for participants receiving Best Supportive Care, but must still be recorded in the participant's medical notes.

Information about adverse reactions, whether volunteered by the participant, discovered by investigator by questioning or detected through physical examination, laboratory test or other investigation will be collected and recorded on the CRF and will be evaluated for duration and intensity according to the National Cancer Terminology Criteria for Adverse Events (NCI CTCAE) v4.0 [Appendix F].

14.3 Recording and reporting SAEs and SUSARs

14.3.1 Events not to be classed as SAEs
The following events will not be recorded as SAEs within the trial:

Hospitalisation for:

- Routine treatment or monitoring of GO cancer not associated with any deterioration in condition.
- Treatment which was elective or pre-planned, for a pre-existing condition not associated with any deterioration in condition.
- Admission to hospital or other institution for general care, not associated with any deterioration in condition.
- Treatment on an emergency outpatient basis for an event not fulfilling any of the definitions for serious as given above, and not resulting in hospital admission.

Death due to progression of disease (unless within 30 days of the last administration of study treatment) and diagnosis of disease progression **do not** require reporting as an SAE, as these are the results of the disease under study and are incorporated into the endpoints of the trial.

14.3.2 Events classed as expected SAEs

Examples of events which will be classed as expected SAEs within this trial and therefore will **not** be reportable as SUSARs are given below. This is not intended to be an exhaustive list, therefore when determining whether an SAE is expected or not, please always refer to the Reference Safety Information (section 4.8) of the relevant SPC supplied in the Investigator Site File (or the latest version as instructed by the CTRU).

Examples of expected SAEs related to GO cancer:

- Anaemia
- Fatigue
- Abdominal pain
- Shortness of breath
- Weight loss

Examples of expected SAEs related to oxaliplatin or capecitabine

- Anaemia
- Neutropenia
- Thrombocytopenia
- Leucopenia
- Febrile neutropenia
- Peripheral sensory neuropathy
- Dizziness
- Dyspnoea
- Cough
- Epistaxis
- Increased liver markers
- Stomatitis
- Lethargy
- Pulmonary embolism
- Nausea / vomiting
- Anorexia
- Diarrhoea
- Dyspepsia
- GO reflux
- Gastrointestinal haemorrhage
- Rectal haemorrhage
- Injection site reaction / pain
- Acute kidney injury
- Hand-foot syndrome

14.3.3 Reporting and recording requirements for SAEs and SUSARs (BSC arm)

For participants randomised to BSC, SAEs and SUSARs will **not** be collected in this trial, but must still be recorded in the participant's medical notes.

14.3.4 Reporting and recording requirements for SAEs and SUSARs (OxCap arms)

The active monitoring period for participants randomised to OxCap, occurs from the time of randomisation until 30 days after OxCap is **permanently** ceased. During this period, all SAEs and SUSARs occurring must be recorded on the SAE or SUSAR CRF and faxed to the CTRU **within 24 hours** of the research staff becoming aware of the event.

All SARs or SUSARs that the investigator becomes aware of after the end of the active monitoring period, must also be recorded on the SAE or SUSAR CRF and faxed to CTRU within 24 hours of the research staff becoming aware of the event.

For each **SAE / SUSAR** the following information will be collected:

- full details in medical terms and case description. The event should be broken down into the primary event e.g. the initial cause of hospitalisation, and concurrent events i.e. events that whilst not the direct cause of hospitalisation are present or develop at this time and therefore are considered part of the overall event. Each component should be graded according to NCI CTCAE v4.0.
- event duration (start and end dates, if applicable).
- action taken.
- outcome.
- seriousness criteria.
- causality (i.e. relatedness to trial drug / investigation), in the opinion of the investigator.
- whether the event would be considered expected or unexpected.

Any change of condition or other follow-up information should be faxed to the CTRU as soon as it is available or at least within 24 hours of the information becoming available. Events will be followed up until the event has resolved or a final outcome has been reached.

In the event that fax machines are no longer in use at a site then please contact CTRU to discuss.

Once all resulting queries have been resolved, the CTRU will request the original form to also be posted to the CTRU and a copy to be retained on site.

All SAEs assigned by the PI or delegate (or following central review) as both suspected to be related to IMP-treatment and unexpected will be classified as SUSARs and will be subject to expedited reporting to the Medicines and Healthcare products Regulatory Authority (MHRA). The CTRU will inform the MHRA, the main Research Ethics Committee (REC) and the Sponsor of SUSARs within the required expedited reporting timescales.

14.4 Responsibilities

Principal Investigator (PI):

1. Checking for AEs and ARs when participants attend for treatment / follow-up.
2. Using medical judgement in assigning seriousness, causality and expectedness using the Reference Safety Information approved for the trial.
3. Ensuring that all SAEs and SARs (including SUSARs) are recorded and reported to the CTRU within 24 hours of becoming aware of the event and provide further follow-up information as soon as available. Ensuring that SAEs and SARs (including SUSARs) are chased with CTRU if a record of receipt is not received within 2 working days of initial reporting.

4. Ensuring that AEs and ARs are recorded and reported to the CTRU in line with the requirements of the protocol.

Chief Investigator (CI) / delegate or independent clinical reviewer:

1. Clinical oversight of the safety of trial participants, including an ongoing review of the risk / benefit.
2. Using medical judgement in assigning seriousness, causality and expectedness of SAEs where it has not been possible to obtain local medical assessment.
3. Immediate review of all SUSARs.
4. Review of specific SAEs and SARs in accordance with the trial risk assessment and protocol as detailed in the Trial Monitoring Plan.
5. Assigning Medical Dictionary for Regulatory Activities (MedDRA) or Body System coding to all SAEs and SARs.
6. Preparing the clinical sections and final sign off of the Development Safety Update Report (DSUR).

CTRU:

1. Central data collection and verification of ARs, SAEs, SARs and SUSARs according to the trial protocol onto a MACRO™ database.
2. Reporting safety information to the CI, delegate or independent clinical reviewer for the ongoing assessment of the risk / benefit according to the Trial Monitoring Plan.
3. Reporting safety information to the independent oversight committees identified for the trial (Data Monitoring & Ethics Committee (DMEC) and Trial Steering Committee (TSC)) according to the Trial Monitoring Plan.
4. Expedited reporting of SUSARs to the Competent Authority (MHRA in UK), main REC and Sponsor within required timelines. The Sponsor will have the opportunity to review and comment if applicable.
5. Notifying Investigators of SUSARs that occur within the trial.
6. Checking for (annually) and notifying PIs of updates to the Reference Safety Information for the trial.
7. Preparing standard tables and other relevant information for the DSUR in collaboration with the CI and ensuring timely submission to the MHRA and main REC.

Trial Steering Committee (TSC):

In accordance with the Trial Terms of Reference for the TSC, periodically reviewing safety data and liaising with the DMEC regarding safety issues.

Data Monitoring & Ethics Committee (DMEC):

In accordance with the Trial Terms of Reference for the DMEC, periodically reviewing unblinded overall safety data to determine patterns and trends of events, or to identify safety issues, which would not be apparent on an individual case basis.

15 Endpoints

15.1 Chemotherapy intensity comparison

Primary endpoint

- Progression-free survival

Secondary endpoints

- Participant reported fatigue
- Time to deterioration of participant reported fatigue
- Overall Treatment Utility
- QoL & symptoms
- Toxicity
- Overall survival
- Quality adjusted survival
- Best response

15.2 Chemotherapy vs BSC comparison (exploratory)

Primary endpoint

- Overall survival

Secondary endpoints

- Participant reported fatigue
- QoL

15.3 Endpoint definitions

- Progression-free survival is defined as the time from randomisation to first documented evidence of disease progression or death from any cause within the 1 year follow up period for each participant. This can be clinical progression as defined in section 12.11 or, for RECIST evaluable disease, radiological progression by RECIST principles. Participants who do not progress will be censored at the last date they were known to be alive and progression free. Details on progression will be reported by sites up to 1 year follow-up, from the date of randomisation.
- Time to deterioration of participant reported fatigue is defined as the time from randomisation to a large deterioration of QLQ-C30 fatigue as compared from participant's baseline fatigue score[28]. Participants who have died within 1 year of randomisation without experiencing a large deterioration of fatigue will be considered as having a competing-risk event at their date of death. Participants who do not experience a large deterioration of fatigue but are not known to have died within 1 year of randomisation will be censored at their last questionnaire completion date.
- Overall Treatment Utility is a novel composite clinical outcome incorporating objective and participant-reported outcome measures of anticancer efficacy, tolerability and acceptability of treatment. It provides a simple "good, intermediate or poor" categorisation of outcome, suitable for analysis against baseline health status and treatment allocation. OTU will be further developed in GO2 to incorporate participant reported fatigue. Full details of OTU scoring are included in Appendix H.

- Participant reported outcomes, including QLQ-C30 fatigue and QoL and symptoms, are based on a Comprehensive Health Assessment (CHA) completed at baseline and a Limited Health Assessment (LHA) completed at 9 weeks post-randomisation (for participants randomised to OxCap),short follow up questionnaires at 18, 27, 36 and 52 weeks. N.B entered under protocol V4.0 and previous versions were also required to complete more frequent short follow up QoL questionnaires.
- Toxicity will be recorded based on adverse events, as graded by CTCAEv4.0, at each chemotherapy cycle and upon cessation of treatment.
- Overall survival is defined as the time from randomisation to death from any cause. Participants who are not known to have died will be censored at the last date they were known to be alive. Deaths will be reported by sites up to approximately 1 year after the last participant is randomised.
- Quality adjusted survival will use the EQ-VAS to weight QoL based on participant preferences.
- For the best response endpoint, the population of participants with disease evaluable by RECIST criteria will be defined and for these participants, a CT scan at 9 and 18 weeks, and as clinically indicated thereafter whilst on chemotherapy, is requested.
- The impact of baseline frailty on outcomes and treatment effect will be assessed for the progression-free survival, overall survival, overall treatment utility, QoL & symptoms and toxicity endpoints. Frailty is defined using the CHA completed at baseline, as given in Appendix J.

16 Statistical Considerations

16.1 Sample size

The length of the recruitment period for GO2 is 4 years, with no fixed sample size. We aim to recruit a minimum of 500 participants to the certain pathway, and an additional 60 participants to the uncertain pathway during this recruitment period. Primary outcome based on chemotherapy intensity comparison (comparison A): Progression-free survival

Initial analysis of data from 321GO, based on a median follow-up of 5.9 months (IQR 2.7-9.5 months), suggests that the overall median PFS for patients with advanced GO cancer who are not fit for full dose EOxCap but suitable for reduced dose chemotherapy is 132 days (95% CI 84 to 169 days).

Although the 95% confidence intervals around the median PFS estimate are wide, and the estimate could change with further follow-up, we are confident that the true rate will be nearer to 132 days (4.4 months) than the limits of the confidence intervals. This is based on a comparison of the FOCUS2, FOCUS, REAL1 and REAL2 trials and the PFS ratio of ‘non-fit’ to ‘fit’ patients in the different populations as follows:

Table 4. Median PFS/FFS estimates in FOCUS2, FOCUS, REAL1 and REAL2 trials

Trial	Patient population	Median PFS/FFS estimate
FOCUS2[23]	Colorectal cancer 'non-fit' patients	Median PFS was 3.5, 5.8, 5.2 and 5.8 months in the FU, OxFU, Cap and OxCap groups respectively; a reasonable estimate for all FOCUS2 patients therefore is 5 months
FOCUS[29]	Colorectal cancer 'fit' patients	Median PFS was 6.3, 8.5 and 8.7 months in the fluorouracil, irinotecan + fluorouracil and oxaliplatin + fluorouracil first line therapy groups respectively; a reasonable estimate for all first line FOCUS patients therefore is 8 months
The ratio of non-fit:fit patients with colorectal cancer is therefore 5:8 i.e. 63%		
REAL1[21]	Oesophagogastric cancer 'fit' patients	Median FFS was 7 months in both the ECF and MCF groups
REAL2[30]	Oesophagogastric cancer 'fit' patients	Median PFS was 6.2, 6.7, 6.5 and 7.0 months in the ECF, ECX, EOF and EOX groups respectively; a reasonable estimate for all REAL2 patients therefore is 6.5 months

Therefore applying the same ratio of 'non-fit' to 'fit' patients seen in colorectal cancer to GO cancer, we can estimate median PFS for GO2 participants to be around 4-4.4 months using the REAL1 and REAL2 trials as the point of reference.

To determine an acceptable non-inferiority margin for the lower-dose treatments in GO2 (Level B OxCap and Level C OxCap), extensive consultation has been undertaken with clinical groups, including the Upper GI Clinical Studies Group, 321GO investigators and user groups:

- Clinical feedback has suggested a median PFS detriment of no more than 1 month in absolute terms (i.e. from 132 to 102 days), or a hazard ratio of around 1.25 in relative terms.
- The over-riding opinion from our patient and public involvement (PPI) representatives is that the optimal balance between survival and QoL will vary widely between individual patients, making it difficult to reach a consensus; however they have indicated that they would accept a larger loss in efficacy than clinicians in return for gains in QoL. Most considered a reduction of up to 6 weeks in median PFS to be acceptable. They commented that *"some information is better than none when patients are faced with treatment decisions"* and suggested that we might concentrate on what non-inferiority margin was feasible to observe rather than setting an arbitrary or unachievable target.

Given these differences, and the anticipated recruitment rate in this population (see section 16.3), rather than specifying an absolute target sample size, it is more appropriate to specify a minimum

together with a target length of recruitment, with the aim to recruit as many participants as possible in this time. Recruiting more than the minimum number of participants will accommodate the uncertainty in the underlying sample size assumptions and will reduce the variability of treatment effect estimates in the analysis.

The table below provides a range of estimates showing what non-inferiority margins can be achieved for differing sample sizes. The sample sizes considered relate to the chemotherapy arms within the certain pathway in the trial; the BSC vs. Level C OxCap comparison (uncertain pathway) is considered in section 16.4.

- GO2 aims to recruit a minimum of 500 participants to the certain pathway (167 per chemotherapy dose intensity – Level A, Level B, Level C); this will give a non-inferiority margin of 34 days median PFS in absolute terms, or HR non-inferiority boundary = 1.34 (80% power; 1-sided 5% significance level, based upon a 1-sided log rank test assuming all participants are followed up for 1 year and that the hazard ratio is constant).
- If recruitment into GO2 reaches our upper estimate of 750 participants in the certain pathway (250 per dose intensity), this will give a non-inferiority margin of 28 days median PFS in absolute terms, or HR non-inferiority boundary = 1.27, with the same power.
- If recruitment proves more challenging than expected, a total of 300 participants in the certain pathway (100 per dose intensity) would still allow exclusion of a PFS detriment of 42 days, in line with the consumer view.

These estimates have not accounted for any losses to follow-up as drop-out is assumed to be minimal given the short survival expectancy of these patients and their high dependency on medical services (zero drop-out was noted in 321GO). Losses will have a small impact on the non-inferiority margin; for example, with 500 participants, a 5% drop-out rate would result in an increase in the non-inferiority margin from 34 to 35 days and the HR boundary from 1.34 to 1.35.

Table 5. Anticipated non-inferiority margins based on differing sample sizes

**Recruitment length**	**Number of participants/dose intensity; total denotes certain pathway only, assuming no dropout**	**Number of PFS events for each comparison**	**HR non-inferiority boundary**	**Reduction in median PFS (days)<br>(=non-inferiority margin)**
4 years	167 (500 in total)	284	1.34	34
	184 (550 in total)	314	1.32	32
	200 (600 in total)	341	1.31	31
	217 (650 in total)	370	1.30	30
	235 (700 in total)	401	1.28	29
	250 (750 in total)	427	1.27	28

16.2 Quality of Life

The primary endpoint in GO2 of the chemotherapy intensity comparison is progression-free survival therefore no formal power calculation has been performed for the quality of life outcomes. However, using the operational definitions by Cohen[31], where a small effect size is defined to be between 0.2 and 0.5, a moderate effect size is defined to be between 0.5 and 0.8 and a large effect size is defined to be >0.8, a sample size of 500 participants in the certain pathway (167 per dose intensity) would give an effect size of 0.307 with 80% power and a 2-sided 5% significance level, whilst a sample size of 750 participants in the certain pathway (250 per dose intensity) would give an effect size of 0.251. However it is acknowledged that this does not take into consideration questionnaire non-compliance. In 321GO, where questionnaires were administered by research nurses in clinic, follow-up compliance was approximately 70%. Assuming this compliance for GO2 gives effect sizes of 0.368 and 0.300 when recruiting 500 and 750 participants to chemotherapy respectively. Therefore given Cohen's definitions, it is expected that we will be able to detect small effect sizes i.e. small improvements in quality of life between the different dose intensities.

16.3 Recruitment

We aim to recruit a minimum of 500 participants to the certain pathway over 3 years.

The primary endpoint of the 321GO feasibility study was recruitment. It took place across two NCRN local networks (one large, one small; total population 3.6M), including 2 tertiary centres and 4 district general hospitals. 321GO recruited 55 participants over a per-site average of 18 months.

- Mean rate per NCRN network = 18.3 participants / year
- Mean rate per participating centre = 6 participants / year (12.6 per tertiary centre; 3.6 per district hospital)

The 2 networks in 321GO cover 5.7% of the UK population. A national study recruiting across all networks at the same rate would recruit 1895 participants over 3 years, however we cannot hope to recruit in every part of the country and a national study would certainly be expected to achieve no more than half of this rate, i.e. **950 participants over 3 years**.

Best-case scenario: If recruitment into GO2 is at the same per-centre rate as 321GO (6 participants per centre per year) then a minimum of 28 centres is needed to recruit 500 participants over 3 years, assuming all centres open at the beginning of the recruitment period.

Worst-case scenario: If recruitment is at half the rate of 321GO (3 participants per centre per year) then a minimum of 56 centres is required.

As recruitment has proved to be slower than anticipated, the recruitment period has been extended to 4 years, which has been discussed and agreed with the TSC, in order to meet the target of 500.

16.4 Primary outcome based on chemotherapy vs BSC comparison (comparison B; exploratory): Overall survival

The inclusion of a BSC arm is exploratory; in order to estimate the outcome in the BSC arm and compare it against chemotherapy we would need at least 30 participants in this arm.[32]

Historical trials that compared chemotherapy with best supportive care (BSC) were summarised by Wagner et al. in their systematic review and meta-analysis[9] (see table below).

Table 6. Overall survival results of Wagner et al's meta-analysis of chemotherapy vs BSC

Study	Sample size		Hazard ratio	95% CI
	Chemo	BSC		
Murad 1993[33]	30	10	0.33	0.17 - 0.64
Pyrhonen 1995[34]	21	20	0.25	0.13 - 0.47
Scheithauer 1996[35]	52	51	0.49	0.33 - 0.74
**Total (fixed effects)**	**103**	**81**	**0.39**	**0.28 - 0.52**

These trials demonstrated an increase in median survival of around 6 months with the addition of chemotherapy to BSC, from around 3 months in the BSC arms to around 9 months in the treatment arms.

Therefore, although the emphasis of the chemotherapy vs. BSC comparison in GO2 is exploratory, with 30 participants in each of the BSC and Level C OxCap arms via the uncertain pathway, we calculate there to be sufficient power (80%) to detect a clinically relevant and justifiable hazard ratio of 0.43 (using a 5% 2-sided significance level) for overall survival based on a median overall survival of 3 months in the BSC arm.

Recruitment into the uncertain benefit decision pathway will end after the inclusion of 60 participants or the completion of four years of recruitment. An additional 60 participants will therefore be required overall in the trial – this adds 12% to the required sample size.

As the chemotherapy vs. BSC comparison is exploratory, the uncertain benefit decision pathway may be stopped early or otherwise adapted on the advice of the DMEC and TSC. Decisions will be based on recruitment feasibility, revised power calculations or emerging evidence of harm.

17 Statistical Analysis

17.1 General considerations

Statistical analysis is the responsibility of the CTRU Statistician. A full statistical analysis plan will be written before any analyses are undertaken. The analysis plan will be written in accordance with the current CTRU standard operating procedures and will be finalised and agreed by the following people: the Trial Statistician, Supervising Statistician, the Chief Investigators, the CTRU Principal Investigator and the Trial Manager. Any changes to the finalised analysis plan, and reasons for changes, will be documented.

Analysis of the primary endpoint (chemotherapy intensity comparison) will be performed on both the intention-to-treat (ITT) population and the per-protocol (PP) population. If a difference is seen between these analyses, the remaining endpoints (excluding best response and toxicity) will also be performed on both populations. If no difference is seen between the ITT and PP populations, the remaining endpoints will be performed on the ITT population only. For the superiority endpoints, the ITT analysis will be given primacy, however for the non-inferiority endpoints, equal weighting will be given to both the ITT and per-protocol analyses, as the ITT is likely to be the least conservative approach when testing for non-inferiority.

The toxicity endpoint will be analysed using the safety population and the best response endpoint will be analysed using the RECIST evaluable population.

The intention-to-treat population will consist of all patients randomised into the trial regardless of whether they were eligible and/or remained in the trial. In this population, patients will be grouped according to the treatment they were randomised to receive.

The per-protocol population will consist of participants who are not classed as major protocol violators, as defined in the statistical analysis plan. Participants will be summarised according to the treatment received in the first treatment cycle.

The safety population will include all participants who receive at least one dose of any trial treatment. Analyses based on the safety population will first summarise participants according to their starting dose (i.e. treatment received in the first treatment cycle), but may also be summarised taking into account dose reductions, as deemed appropriate.

The RECIST evaluable population will include all participants who had disease which was evaluable by RECIST criteria at baseline.

Hypothesis testing will be two-sided for superiority endpoints and one-sided for non-inferiority endpoints and performed at a 5% significance level.

Sensitivity analyses may be performed for each endpoint, for example to take into account differing assumptions about missing data if there is a significant number of missing data, and will be detailed in the full statistical analysis plan.

A DMEC will be set up to meet at least annually to independently review interim efficacy, safety and recruitment data. No formal interim analyses are planned so no statistical testing will take place until final analysis. Final analysis will take place once the minimum required number of PFS events have occurred (as specified in the sample size calculation): 284 in each comparison in the certain benefit pathway (level B vs level A and Level C vs Level A); or when the most

recently randomised, surviving participant has been followed up for 1 year post randomisation, whichever is reached sooner.

17.2 Comparisons

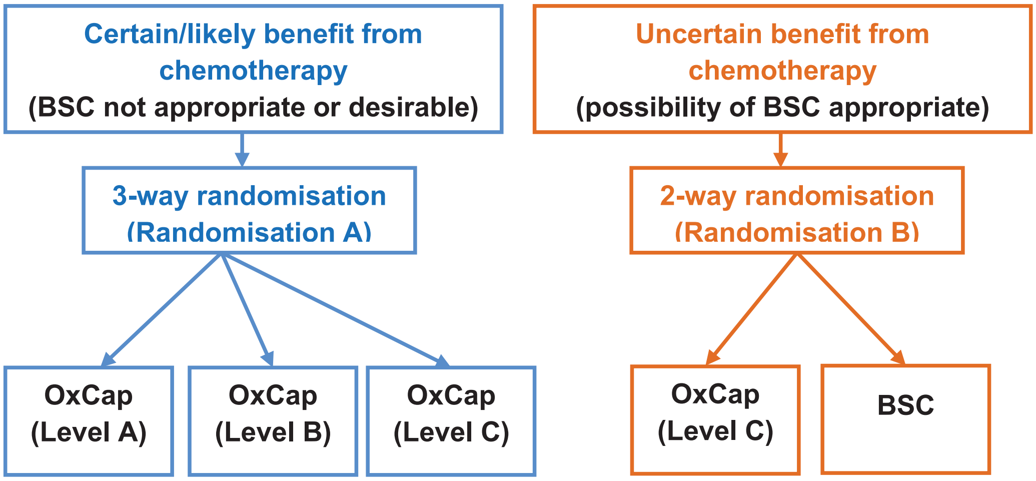

N.B. Participants entered into the uncertain pathway under protocol version 3.0 are randomised to Level A OxCap, Level B OxCap, Level C OxCap or BSC.

Chemotherapy intensity comparison

In order to determine the optimal chemotherapy dose intensity in GO2, the different intensity chemotherapy arms in the certain/likely benefit pathway will be compared. Level B and level C will be compared to level A (i.e. two different comparisons).

Participants entered into the uncertain pathway under protocol version 3.0 are randomised to Level A OxCap, Level B OxCap, Level C OxCap or BSC. Those who were randomised to one of the chemotherapy arms will be included in the chemotherapy intensity question (small number of participants, expected to be less than 5). Participants entered into the uncertain pathway under protocol version 4.0 onwards will not be included in the chemotherapy intensity comparison.

Chemotherapy vs BSC comparison

The best supportive care arm will be used to consider if chemotherapy is worthwhile for participants in the uncertain benefit pathway and will compare BSC with Level C OxCap. Analysis of this comparison will be exploratory in nature.

17.3 Chemotherapy intensity comparison

17.3.1 Primary endpoint: Progression-free survival (non-inferiority)

Analysis of PFS up to 1 year follow up of each participant will be based on the 90% confidence interval (CI) (one-sided type I error rate of 5.0%) of the hazard ratio (HR); the 90% CI of the difference in median PFS will also be presented to aid interpretation. We will look at non-inferiority (with the same margin of non-inferiority) of both Level B OxCap and Level C OxCap compared with Level A OxCap i.e. two separate comparisons.

The upper limit of the 90% CI, for each comparison, will be compared with the non-inferiority margin of HR = 1.34, which is equivalent to a non-inferiority margin of 34 days when presenting differences in median PFS. If it is below this margin for either comparison, then the result will be taken as evidence that Level B OxCap or Level C OxCap (depending upon the comparison) is not inferior to Level A OxCap. If the upper limit is above the non-inferiority margin, then non-inferiority will not have been demonstrated. Given the lack of consensus however in what loss in efficacy would be deemed acceptable to be able to claim non-inferiority, sensitivity analyses for the choice of non-inferiority margin will be carried out.

Progression-free survival curves will be calculated using the Kaplan-Meier method. Participants without a PFS event at the time of analysis will be censored at the time they were last known to be alive and progression-free. Median progression-free survival estimates with corresponding 90% confidence intervals will be presented by treatment group. A log-rank test, stratifying for the minimisation factors will be used to compare progression-free survival between the treatment groups.

Cox's Proportional Hazards model, if appropriate, adjusting for the minimisation factors, will also be used to compare PFS between the treatment groups. Treatment HRs and corresponding 90% CIs will be obtained, and the upper limit of the CI for PFS compared with the non-inferiority margin. Treatment and covariate estimates, standard errors, hazard ratios, 90% confidence intervals and p-values will be presented for all variables incorporated in the model.

17.3.2 Secondary endpoints

17.3.2.1 Overall treatment utility (superiority)

Overall treatment utility (OTU) will be calculated as per Appendix H at 9 weeks post randomisation and summarised by calculating the differences in rates between the treatment groups with corresponding 95% CIs. Treatment groups will be compared using ordered logistic regression to adjust for the minimisation factors. Treatment and covariate estimates, standard errors, odds ratios, 95% confidence intervals and p-values will be presented for all variables incorporated in the model. We will compare both Level B OxCap and Level C OxCap with Level A OxCap i.e. two separate comparisons.

17.3.2.2 Time to deterioration of participant reported fatigue (superiority)

Time to deterioration of participant reported fatigue will be investigated using cumulative incidence function curves and the median time to deterioration and 95% confidence intervals will be presented by treatment group. Participants without deterioration of fatigue and who are not known to have died within 1 year of randomisation will be censored at their last questionnaire completion date. Participants who have died within 1 year of randomisation without evidence of deterioration of fatigue will be censored at their date of death in the analysis estimating the treatment effect (i.e. the log-rank test / Cox's Proportional Hazards model) and classed as having a competing-risk event (i.e. not censored) in the analysis estimating the incidence of deterioration of fatigue (i.e. the cumulative incidence function curves). We will compare both Level B OxCap and Level C OxCap with Level A OxCap i.e. two separate comparisons.

A log-rank test, stratifying for the minimisation factors, will be used to compare time to deterioration of fatigue between the treatment groups. Cox's Proportional Hazards model, if appropriate, adjusting for the minimisation factors, will also be used to compare time to

deterioration of fatigue between the treatment groups. Treatment and covariate estimates, standard errors, hazard ratios, 95% confidence intervals and p-values will be presented for all variables incorporated in the model.

17.3.2.3 Participant reported fatigue, QoL & symptoms (superiority)

Quality of life, including fatigue, global QoL and symptoms, will be summarised for each treatment arm at each post-randomisation time-point, using adjusted for baseline mean scores and 95% CIs. These summaries and differences between treatment arms will be obtained and compared using a multi-level repeated measures model accounting for data at all post-baseline time points, regardless of time of completion for the time-point not of interest, assuming missing data at random [MAR] and allowing for time, treatment, treatment-time interaction, and adjusting for baseline QoL and the minimisation factors[fixed effects] and for participant and participant-time interaction [random effects] where appropriate. Data will also be summarised descriptively using bar charts, box plots and summary tables. Missing data patterns will be examined carefully and alternative analyses using different missing data assumptions will be performed if appropriate. We will compare both Level B OxCap and Level C OxCap with Level A OxCap i.e. two separate comparisons.

If a significant number of data is missing and missing data patterns suggest data are missing not at random, to allow for differing assumptions about missing data, analyses will also be carried out using: pattern-mixture multi-level models categorising participants into strata based on clinical information which is believed to represent the reasons for missing data (assuming MAR data conditional upon participants' clinical data); and pattern mixture models for bivariate (baseline and 9 week) data fitted using a variety of restrictions reflecting the missing data pattern ranging from complete case missing variable restriction (MAR) to Brown's protective restriction (assuming data are missing not at random (MNAR)).

17.3.2.4 Toxicity

To assess toxicity, the rate of CTCAEv4.0 grade ≥ 2 haematological and non-haematological toxicities, the maximum grade per participant for each toxicity and rates of toxicities overall and per cycle will be summarised descriptively for each treatment group. Treatment delays, modifications and withdrawals will also be summarised together with additional safety data e.g. SAEs, SARs, SUSARs and deaths within 30 days of last treatment administration or which are considered to be related to treatment.

17.3.2.5 Overall survival (non-inferiority)

Overall survival (OS) curves will be calculated using the Kaplan-Meier method and the median overall survival estimates and 90% confidence intervals will be presented by treatment group. Participants without an OS event at the time of analysis will be censored at the time they were last known to be alive. We will compare both 80% OxCap and 60% OxCap with 100% OxCap i.e. two separate comparisons.

A log-rank test, stratifying for the minimisation factors, will be used to compare overall survival between the treatment groups. Cox's Proportional Hazards model, if appropriate, adjusting for the minimisation factors, will also be used to compare OS between the treatment groups. Treatment and covariate estimates, standard errors, hazard ratios, 90% confidence intervals and p-values will be presented for all variables incorporated in the model.

17.3.2.6 Quality adjusted survival (superiority)

Quality adjusted survival (QAS) will be calculated up to 1 year follow up for each participant using the methods described by Billingham and Abrams.[36] This will rely on the longitudinally measured EQ-VAS to weight QoL based on participant preferences. Initial analysis will rely on the integrated quality-survival product. The analysis will be repeated for QoL weight measured by the EQ-5D tariff. The role of missing data will be tested by sensitivity analysis and, where appropriate, by imputation. If missing data are thought to be causing bias in QAS or if there is a need to extrapolate survival and QoL outcomes beyond the available data then a second approach will use a multistate transition model with dropout-specific and health-specific states. We will compare both Level B OxCap and Level C OxCap with Level A OxCap i.e. two separate comparisons.

17.3.2.7 Best response (non-inferiority)

Best response within 1 year of randomisation will be summarised by the proportion of participants achieving either a complete response, a partial response or stable disease.[37] The differences in rates between the treatment groups will be presented with corresponding 90% CIs and compared using logistic regression to adjust for the minimisation factors. Treatment and covariate estimates, standard errors, odds ratios, 90% confidence intervals and p-values will be presented for all variables incorporated in the model. We will compare both Level B OxCap and Level C OxCap with Level A OxCap i.e. two separate comparisons.

17.3.2.8 Frailty analyses

The impact of baseline frailty on outcomes and treatment effect will be assessed for the progression-free survival, overall survival, overall treatment utility, QoL & symptoms and toxicity endpoints, using the methods summarised above for each endpoint.

The primary analysis of frailty will use impairment in two or more domains (as given in Appendix J) as the cut-off for frailty to define participants as frail or not frail. Both the prognostic and predictive effect of baseline frailty will be assessed, incorporating a frailty-treatment interaction term in multivariate models where appropriate, and performing a subgroup analysis by frailty where this is not possible (e.g. for the toxicity endpoint). Analyses of ordinal CGA scores will also be performed to determine whether increasing score is associated with worse outcomes and to assess heterogeneity of the treatment effect on outcomes.

17.3.3 Further analyses

17.3.3.1 Subgroup analyses

Subgroup analyses for the clinical randomisation factors and other baseline participant characteristics will be performed to investigate whether there is heterogeneity of treatment effect on outcomes.

17.3.3.2 Exploratory prognostic factor analyses

Baseline participant characteristics and items in the CHA will be investigated to determine whether they are prognostic of outcomes.

17.4 Chemotherapy vs BSC comparison (exploratory)

17.4.1 Primary endpoint: Overall survival (superiority)

Overall survival (OS) curveswill be calculated using the Kaplan-Meier method and the median overall survival estimates and 95% confidence intervals will be presented by treatment group. Analysis of this endpoint concerns the superiority of Level C OxCap over best supportive care in terms of overall survival. A log-rank test, stratifying for the minimisation factors, will be used to compare overall survival between the treatment groups. Participants without an OS eventat the time of analysis will be censored at the time they were last known to be alive.

Cox's Proportional Hazards model, if appropriate, adjusting for the minimisation factors, will also be used to compare OS between the treatment groups. Treatment and covariate estimates, standard errors, hazard ratios, 95% confidence intervals and p-values will be presented for all variables incorporated in the model.

17.4.2 Secondary endpoints

17.4.2.1 Participant reported fatigue and QoL (superiority)

Quality of life, including fatigue, will be summarised for each treatment arm at each post-randomisation time-point, using adjusted for baseline mean scores and 95% CIs. These summaries and differences between treatment arms will be obtained and compared using a multi-level repeated measures model accounting for data at all post-baseline time points, regardless of time of completion for the time-point not of interest, assuming missing data at random [MAR] and allowing for time, treatment, treatment-time interaction, and adjusting for baseline QoL and the minimisation factors [all fixed effects] and for participant and participant-time interaction [random effects] where appropriate. Data will also be summarised descriptively using bar charts, box plots and summary tables. Missing data patterns will be examined carefully and alternative analyses using different missing data assumptions will be performed if appropriate.

18 Trial Monitoring

18.1 Trial steering committee and data monitoring and ethics committee

A Trial Monitoring Plan will be developed and agreed by the Trial Management Group (TMG) and TSC based on the trial risk assessment.

An independent DMEC will review the safety and ethics of the study. Detailed un-blinded reports will be prepared by the CTRU for the DMEC at approximately 12-monthly intervals.

18.2 Data monitoring

Data will be monitored for quality and completeness by the CTRU. Missing data will be chased until it is received, confirmed as not available or the trial is at analysis. However missing data items will not be chased from participants (although research nurses will perform a check of questionnaires completed in clinic). The CTRU/Sponsor will reserve the right to intermittently conduct source data verification exercises on a sample of participants, which will be carried out by staff from the CTRU/Sponsor. Source data verification will involve direct access to patient notes at the participating hospital sites and the ongoing central collection of copies of consent forms and other relevant investigation reports.

18.3 Clinical governance issue

To ensure responsibility and accountability for the overall quality of care received by participants during the study period, clinical governance issues pertaining to all aspects of routine management will be brought to the attention of the TSC, Sponsor and, where applicable, to individual NHS Trusts.

19 Quality Assurance and Ethical Considerations

19.1 Quality assurance

The trial will be conducted in accordance with the principles of Good Clinical Practice (GCP) in clinical trials, as applicable under UK regulations, the NHS Research Governance Framework (RGF) and Scottish Executive Health Department Research Governance Framework for Health and Social Care 2006, and through adherence to CTRU Standard Operating Procedures (SOPs).

19.2 Serious breaches

CTRU and Sponsor have systems in place to ensure that serious breaches of GCP or the trial protocol are picked up and reported. Investigators are required to promptly notify the CTRU of a serious breach (as defined in Regulation 29A of the Medicines for Human Use (Clinical Trials) Regulations 2004 and amendments) that they become aware of. A 'serious breach' is a breach which is likely to effect to a significant degree –

(a) the safety or physical or mental integrity of the subjects of the trial; or

(b) the scientific value of the trial.

In the event of doubt or for further information, the Investigator should contact the Senior Trial Co-ordinator at the CTRU.

19.3 Ethical considerations

The trial will be performed in accordance with the recommendations guiding physicians in biomedical research involving human subjects adopted by the 18th World Medical Assembly, Helsinki, Finland, 1964, amended at the 52nd World Medical Association General Assembly, Edinburgh, Scotland, 1996. Informed written consent will be obtained from the participants prior to randomisation into the study. The right of a participant to refuse participation without giving reasons must be respected. The participant must remain free to withdraw at any time from the study without giving reasons and without prejudicing his/her further treatment. The study will be submitted to and approved by a main REC and the appropriate Site Specific Assessor for each participating centre prior to entering participants into the study. The CTRU will provide the main REC with a copy of the final protocol, patient information sheets, consent forms and all other relevant study documentation.

20 Confidentiality

All information collected during the course of the trial will be kept strictly confidential. Information will be held securely on paper and electronically at the CTRU. The CTRU will comply with all aspects of the Data Protection Act 2018 and operationally this will include:

- consent from participants to record personal details including date of birth, NHS number, hospital number.
- appropriate storage, restricted access and disposal arrangements for participant personal and clinical details.
- consent from participants for access to their medical records by responsible individuals from the research staff or from regulatory authorities, where it is relevant to trial participation.
- consent from participants for the data collected for the trial to be used to evaluate safety and develop new research.
- participant name and NHS number will be collected at baseline, but all other data collection forms that are transferred to or from the CTRU will be coded with a trial number and will include two participant identifiers, usually the participant's initials and date of birth.
- where central monitoring of source documents by CTRU (or copies of source documents) is required (such as scans or local blood results), the participant's name must be obliterated by site before sending.
- where anonymisation of documentation is required, sites are responsible for ensuring only the instructed identifiers are present before sending to CTRU.

If a participant withdraws consent from further trial treatment and / or further collection of data their samples will remain on file and will be included in the final study analysis.

21 Archiving

At the end of the trial, data and the Trial Master File will be securely archived by CTRU in line with the Sponsor's procedures for a minimum of 15 years. Site data and documents will be archived at the participating centres. Following authorisation from the Sponsor, arrangements for confidential destruction will then be made.

22 Statement of Indemnity

The University of Leeds is able to provide insurance to cover for liabilities and prospective liabilities arising from negligent harm. We are also able to provide insurance cover, in certain circumstances, for claims arising from non-negligent harm including the design of the protocol. Clinical negligence indemnification will rest with the participating NHS Trust or Trusts under standard NHS arrangements.

23 Study Organisational Structure

23.1 Individuals and individual organisations

Chief Investigator (CI) – The CI is involved in the design, conduct, co-ordination and management of the trial. The CI will have overall responsibility for the design and set-up of the trial, the investigational drug supply and pharmacovigilance within the trial.

Trial Sponsor – The Sponsor is responsible for trial initiation management and financing of the trial as defined by Directive 2001/20/EC. These responsibilities are delegated to the CTRU as detailed in the trial contract.

Clinical Trials Research Unit – The CTRU will have responsibility for conduct of the trial as delegated by the Sponsor in accordance with relevant GCP standards and CTRU SOPs. The CTRU will provide set-up and monitoring of trial conduct to CTRU SOPs, and the GCP Conditions and Principles as detailed in the UK Medicines for Human Use (Clinical Trials) Regulations 2006 including, randomisation design and service, database development and provision, protocol development, CRF design, trial design, source data verification, monitoring schedule and statistical analysis for the trial. In addition the CTRU will support main REC, Site Specific Assessment and NHS Permissions submissions and clinical set-up, ongoing management including training, monitoring reports and promotion of the trial. The CTRU will be responsible for the day-to-day running of the trial including trial administration, database administrative functions, data management, safety reporting and all statistical analyses.

23.2 Oversight and trial monitoring groups

Trial Management Group (TMG) – The TMG, comprising the CI, CTRU team, other key external members of staff involved in the trial and a nursing representative will be assigned responsibility for the clinical set-up, on-going management, promotion of the trial, and for the interpretation and publishing of the results. Specifically the TMG will be responsible for (i) protocol completion, (ii) CRF development, (iii) obtaining approval from the main REC and supporting applications for Site Specific Assessments, (iv) submitting a CTA application and obtaining approval from the MHRA, (v) completing cost estimates and project initiation, (vi) nominating members and facilitating the TSC and DMEC, (vii) reporting of serious adverse events, (viii) monitoring of screening, recruitment, treatment and follow-up procedures, (ix) auditing consent procedures, data collection, trial end-point validation and database development.

Trial Steering Committee (TSC) – The TSC, with an independent Chair, will provide overall supervision of the trial, in particular trial progress, adherence to protocol, participant safety and consideration of new information. It will include an Independent Chair, not less than two other independent members and a PPI representative. The CI and other members of the TMG may attend the TSC meetings and present and report progress. The Sponsor will be invited to TSC meetings. The Committee will meet annually as a minimum.

Data Monitoring and Ethics Committee (DMEC) – The DMEC will include independent membership and will review the safety and ethics of the trial by reviewing interim data during recruitment and the follow-up period. The Committee will meet annually as a minimum.

24 Publication Policy

The trial will be registered with an authorised registry, according to the International Committee of Medical Journal Editors (ICMJE) Guidelines, prior the start of recruitment.

The success of the trial depends upon the collaboration of all participants. For this reason, credit for the main results will be given to all those who have collaborated in the trial, through authorship and contributorship. Uniform requirements for authorship for manuscripts submitted to medical journals will guide authorship decisions. These state that authorship credit should be based only on substantial contribution to:

- conception and design, or acquisition of data, or analysis and interpretation of data,
- drafting the article or revising it critically for important intellectual content,
- and final approval of the version to be published,
- and that all these conditions must be met (www.icmje.org).

In light of this, the Chief Investigators, and relevant senior CTRU staff will be named as authors in any publication. In addition, all collaborators will be listed as contributors for the main trial publication, giving details of roles in planning, conducting and reporting the trial.

To maintain the scientific integrity of the trial, data will not be released prior to the first publication of the analysis of the primary endpoint, either for trial publication or oral presentation purposes, without the permission of the Trial Steering Committee. In addition, individual collaborators must not publish data concerning their participants which is directly relevant to the questions posed in the trial until the first publication of the analysis of the primary endpoint.

25 References

1. Cancer_Research_UK, *CancerStats.* http://info.cancerresearchuk.org/cancerstats, 2007.
2. Kocher, H.M., et al., *Epidemiological study of oesophageal and gastric cancer in south-east England.* Br J Surg, 2001. **88**(9): p. 1249-57.
3. MacMillan Cancer Support, D.a.A.U., *Cancer Services Coming of Age: Learning from the Improving Cancer Treatment, Assessment and Support for Older People Project.* . http://www.macmillan.org.uk/Documents/AboutUs/Health_professionals/OlderPeoplesProject/CancerServicesComingofAge.pdf, 2012.
4. Janunger, K.G., et al., *A systematic overview of chemotherapy effects in gastric cancer.* Acta Oncologica, 2001. **40**(2-3): p. 309-26.
5. Cunningham, D., et al., *Capecitabine and Oxaliplatin for Advanced Esophagogastric Cancer.* New England Journal of Medicine, 2008. **358**(1): p. 36-46.
6. Hong, Y.S., et al., *A phase II trial of capecitabine in previously untreated patients with advanced and/or metastatic gastric cancer.* Annals of Oncology, 2004. **15**(9): p. 1344-7.
7. Barone, C., et al., *Treatment of patients with advanced gastric carcinoma with a 5-fluorouracil-based or a cisplatin-based regimen: two parallel randomized phase II studies.* Cancer, 1998. **82**(8): p. 1460-7.
8. Ohtsu, A., et al., *Randomized phase III trial of fluorouracil alone versus fluorouracil plus cisplatin versus uracil and tegafur plus mitomycin in patients with unresectable, advanced gastric cancer: The Japan Clinical Oncology Group Study (JCOG9205).* J Clin Oncol, 2003. **21**(1): p. 54-9.
9. Wagner, A.D., et al., *Chemotherapy in Advanced Gastric Cancer: A Systematic Review and Meta-Analysis Based on Aggregate Data.* Journal of Clinical Oncology, 2006. **24**(18): p. 2903-2909.
10. Tebbutt, N.C., et al., *A multicentre, randomised phase III trial comparing protracted venous infusion (PVI) 5-fluorouracil (5-FU) with PVI 5-FU plus mitomycin C in patients with inoperable oesophago-gastric cancer.* Annals of Oncology, 2002. **13**(10): p. 1568-75.
11. Cascinu, S., A. Fedeli, and G. Catalano, *Etoposide, leucovorin, 5-fluorouracil and interferon alpha-2b in elderly gastric cancer patients: a pilot study.* Cancer Chemotherapy & Pharmacology, 1994. **34**(1): p. 72-4.
12. di Bartolomeo, M., et al., *Phase II study of the etoposide, leucovorin and fluorouracil combination for patients with advanced gastric cancer unsuitable for aggressive chemotherapy.* Oncology, 1995. **52**(1): p. 41-4.
13. Lee, J.L., et al., *A randomised multicentre phase II trial of capecitabine vs S-1 as first-line treatment in elderly patients with metastatic or recurrent unresectable gastric cancer.* British Journal of Cancer, 2008. **99**(4): p. 584-90.
14. Santini, D., et al., *Weekly oxaliplatin, 5-fluorouracil and folinic acid (OXALF) as first-line chemotherapy for elderly patients with advanced gastric cancer: results of a phase II trial.* BMC Cancer, 2006. **6**: p. 125.
15. Zang, D.Y., et al., *Phase I/II trial with docetaxel and S-1 for patients with advanced or recurrent gastric cancer with consideration to age.* Cancer Chemotherapy & Pharmacology, 2009. **63**(3): p. 509-16.
16. Trumper, M., et al., *Efficacy and tolerability of chemotherapy in elderly patients with advanced oesophago-gastric cancer: A pooled analysis of three clinical trials.* Eur J Cancer, 2006. **42**(7): p. 827-34.
17. Popescu, R.A., et al., *Adjuvant or palliative chemotherapy for colorectal cancer in patients 70 years or older.* Journal of Clinical Oncology, 1999. **17**(8): p. 2412-8.
18. Al-Batran, S.-E., et al., *Phase III trial in metastatic gastroesophageal adenocarcinoma with fluorouracil, leucovorin plus either oxaliplatin or cisplatin: a study of the Arbeitsgemeinschaft Internistische Onkologie.* Journal of Clinical Oncology, 2008. **26**(9): p. 1435-42.
19. Al-Batran, S.-E., et al., *The feasibility of triple-drug chemotherapy combination in older adult patients with oesophagogastric cancer: A randomised trial of the Arbeitsgemeinschaft Internistische Onkologie (FLOT65+).* European Journal of Cancer, 2013. **49**(4): p. 835-842.
20. Stein, B.N., et al., *Age and sex are independent predictors of 5-fluorouracil toxicity. Analysis of a large scale phase III trial.* Cancer, 1995. **75**(1): p. 11-7.
21. Ross, P., et al., *Prospective randomized trial comparing mitomycin, cisplatin, and protracted venous-infusion fluorouracil (PVI 5-FU) With epirubicin, cisplatin, and PVI 5-FU in advanced esophagogastric cancer.* Journal of Clinical Oncology, 2002. **20**(8): p. 1996-2004.
22. Lord, S.R., et al., *Factors predicting outcome for advanced gastroesophageal cancer in elderly patients receiving palliative chemotherapy.* Clinical Oncology (Royal College of Radiologists), 2010. **22**(2): p. 107-13.
23. Seymour, M.T., et al., *Chemotherapy options in elderly and frail patients with metastatic colorectal cancer (MRC FOCUS2): an open-label, randomised factorial trial.* Lancet, 2011. **377**(9779): p. 1749-59.

24. Schmoll, H.-J., *Do we need oncology trials tailored for the elderly or frail?* The Lancet, 2011. **377**(9779): p. 1725-1727.
25. Hall, P.S., et al., *Three, two, or one drug chemotherapy for frail or elderly patients with advanced gastroesophageal cancer (321GO): A feasibility study.* ASCO Meeting Abstracts, 2012. **30**(4_suppl): p. 97.
26. Griggs, J.J., et al., *Appropriate chemotherapy dosing for obese adult patients with cancer: American Society of Clinical Oncology clinical practice guideline.* Journal of Clinical Oncology, 2012. **30**(13): p. 1553-61.
27. Hall PS, L.S., El-Laboudi A & Seymour MT, *Non-cancer medications for patients with incurable cancer: time to stop and think?* British Journal of General Practice, 2010. **60(573)**(April 2010): p. 243-244.
28. Cocks, K., et al., *Evidence-based guidelines for interpreting change scores for the European Organisation for the Research and Treatment of Cancer Quality of Life Questionnaire Core 30.* European Journal of Cancer, 2012. **48**(11): p. 1713-21.
29. Seymour, M.T., et al., *Different strategies of sequential and combination chemotherapy for patients with poor prognosis advanced colorectal cancer (MRC FOCUS): a randomised controlled trial.[Erratum appears in Lancet. 2007 Aug 18;370(9587):566].* Lancet, 2007. **370**(9582): p. 143-52.
30. Cunningham, D., et al., *Capecitabine and oxaliplatin for advanced esophagogastric cancer.* N Engl J Med, 2008. **358**(1): p. 36-46.
31. Cohen, J., *Statistical power analysis for the behavioural sciences*. 2nd ed. 1988, Hillsdale, N.J: L. Erlbaum Associates.
32. Lancaster, G.A., S. Dodd, and P.R. Williamson, *Design and analysis of pilot studies: recommendations for good practice.* Journal of Evaluation in Clinical Practice, 2004. **10**(2): p. 307-12.
33. Murad, A.M., et al., *Modified therapy with 5-fluorouracil, doxorubicin, and methotrexate in advanced gastric cancer.* Cancer, 1993. **72**(1): p. 37-41.
34. Pyrhonen, S., et al., *Randomised comparison of fluorouracil, epidoxorubicin and methotrexate (FEMTX) plus supportive care with supportive care alone in patients with non-resectable gastric cancer.* British Journal of Cancer, 1995. **71**(3): p. 587-91.
35. Scheithauer W, K.G., Hejna M, et al, *Palliative chemotherapy versus best supportive care in patients with metastatic gastric cancer: A randomized trial.* 1994.
36. Billingham, L.J. and K.R. Abrams, *Simultaneous analysis of quality of life and survival data.* Statistical Methods in Medical Research, 2002. **11**(1): p. 25-48.
37. Therasse, P., et al., *New guidelines to evaluate the response to treatment in solid tumors.* Journal of the National Cancer Institute, 2000. **92**(3): p. 205-216.
38. Eisenhauer, E.A., et al., *New response evaluation criteria in solid tumours: Revised RECIST guideline (version 1.1).* European Journal of Cancer, 2009. **45**(2): p. 228-247.

Appendix A – WHO Performance Status

Clinical Performance Status

0	Able to carry out all normal activity without restriction.
1	Restricted in physically strenuous activity but ambulatory and able to carry out light work.
2	Ambulatory and capable of all self-care but unable to carry out any work; up and about more than 50% of waking hours.
3	Capable only of limited self-care; confined to bed or chair more than 50% of waking hours.
4	Completely disabled; cannot carry out any self-care; totally confined to bed or chair.

Appendix B – Timed get up and go test

WORKING PRACTICE GUIDELINES FOR THE TIMED GET UP AND GO TEST

1. INTRODUCTION

The Timed get up and go test is a tool used for measuring clinically significant changes in mobility in frail patients. It includes a number of tasks such as standing from a seated position, walking, turning, stopping and sitting down, which are all important tasks needed for a person to be independently mobile.

The time taken to complete the task reflects degree of impairment.

2. PREPARATION

- A distance of 3 metres is measured and marked from the front legs of the chair in a straight line
- A straight backed armchair, measuring 46cms from the seat to the ground, is used. The same chair must be used for all assessments within a site.

3. PROCEDURE

- The participant uses their usual footwear and walking aids. If a stick is to be used have this ready in the participant's hand or by the side of the chair if both hands are needed to push themselves up.
- The participant is instructed "Sit with your back against the chair and your arms on the arm rest. On the word GO stand upright, then walk at your normal pace to the line on the floor, turn around, return to the chair and sit down".
- The stop watch is started on the word GO and stopped when the participant returns to the sitting position.
- The time taken to complete the task is written in the CRF.

Appendix C – Capecitabine dose banding

Centres may either follow the dose banding below (Table C1) or adopt local practice, provided this gives doses within10% or 100mg (whichever is larger) of the per protocol calculated dose.

If local dose banding is used, the dose banding schedule should be supplied to CTRU.

Table C1. Suggested capecitabine dose banding

Exact dose (mg)	Banded dose (mg)	Number of tablets	
		500mg	150mg
400 - 474	450	0	3
475 – 549	500	1	0
550 – 624	600	0	4
625 – 699	650	1	1
700 – 774	750	0	5
775 – 849	800	1	2
850 – 924	900	0	6
925 – 974	950	1	3
975 – 1049	1000	2	0
1050 – 1124	1100	1	4
1125 – 1224	1150	2	1
1225 – 1374	1300	2	2
1375 – 1474	1450	2	3
1475 – 1574	1500	3	0
1575 – 1724	1650	3	1
1725 – 1899	1800	3	2
1900 – 2074	2000	4	0
2075 – 2224	2150	4	1
2225 – 2399	2300	4	2
2400 – 2574	2500	5	0
2575 – 2724	2650	5	1
2725 – 2899	2800	5	2
2900 – 3074	3000	6	0

Appendix D - Dose modifications

Note – this guidance should be followed wherever possible; deviation is permitted but should be in line with local practice.

Haematological toxicity

- Check FBC if possible, the day before chemotherapy or at most within 3 days prior to day 1 of each cycle (local practice may be followed). Delay 1 week if neutrophils <1.0 x 10^9/l or platelets <75 x 10^9/l. Only treat when neutrophils and platelets are above these limits.
- If more than 1 delay, or 1 delay of ≥2 weeks occurs, reduce the capecitabine and oxaliplatin doses by 20% and continue at the lower dose for subsequent cycles unless further toxicity occurs.
- If a further delay(s) for myelotoxicity occurs despite a 20% reduction, a further dose reduction may be made, at the discretion of the treating clinician.
- GCSF may be used as per local guidance but should be recorded in the CRF.

Non-haematological toxicities (excluding neurotoxicity – see below)

- Grade 1 toxicity is managed symptomatically and does not usually require dose reduction or interruption
- For any toxicity of grade 2 or higher (e.g. diarrhoea, mucositis, vomiting) **stop capecitabine** and treat symptomatically as per local practice until the toxicity has resolved to grade 0 or 1.
    - Note that when capecitabine is stopped for capecitabine-specific toxicity the **doses are omitted, not delayed**. If resolution to grade 0-1 occurs capecitabine is resumed for the remainder of the planned cycle at the same dose or with a reduction as specified below.
- When resuming OxCap or Cap after a pause for toxicity, use the following dose reduction scheme:
    - Grade 2 toxicity: resume at the same dose after first pause, but reduce both drugs by 25% of the Cycle 1 doses if a second pause is required.
    - Grade 3 toxicity: resume with a reduction of 25% of Cycle 1 doses (both drugs).
    - Grade 4 toxicity: discontinue permanently.
- If further toxicity of grade ≥2 occurs after a dose-reduction, the doses should either be reduced to 50% of the Cycle 1 dose, or stopped permanently, at the consultant's discretion.

Neurotoxicity

- Oxaliplatin commonly causes peripheral sensory symptoms.
- Many patients experience transient paraesthesia of hands and feet, or mild throat dysaesthesia, precipitated by cold and lasting several days after each oxaliplatin administration. This does not require treatment or dose reduction.
- See table below for suggested dose modifications if symptoms persist. If symptoms persist until the next cycle is due, and are associated with significant discomfort or loss of function (e.g. dropping objects), omit oxaliplatin and continue with capecitabine alone until fully recovered, then consider restarting oxaliplatin.

Table D1. Suggested oxaliplatin dose modifications for neurotoxicity

Toxicity	Duration of toxicity 1-7 days	Duration of toxicity >7 days	Persistent between cycles
Cold-related dysaesthesia	No reduction	No reduction.	Withhold oxaliplatin until recovery then restart with 25% reduction of cycle 1 dose. Omit oxaliplatin if recurs.
Paraesthesia without pain	No reduction	No reduction	Withhold oxaliplatin until recovery then restart with 25% reduction of cycle 1 dose. Omit oxaliplatin if recurs.
Paraesthesia with pain	No reduction	Reduce by 25% of cycle 1 dose on subsequent cycles. Omit oxaliplatin if recurs.	Omit Oxaliplatin
Paraesthesia with functional impairment	No reduction	Reduce by 25% of cycle 1 dose on subsequent cycles. Omit oxaliplatin if recurs.	Omit Oxaliplatin

Renal function

- Before starting, ensure patient fulfils eligibility for renal function. A patient with a GFR <30 ml/min is not eligible for enrolment in the trial.
- If a formula-derived GFR is <50 ml/min (e.g. using Wright formula or equivalent), it is recommended that a GFR is measured by radioisotopic clearance or equivalent but local practice can be followed. If this is not available prior to cycle 1 then it is recommended to be arranged prior to cycle 2. The measured GFR should take precedence over the formula-derived GFR.
- If GFR is in the range 30-49 ml/min, a 25% reduction in the dose of capecitabine, relative to cycle 1, is suggested, as per Table D2.
- If renal function changes at any point after randomisation, use Table D2 below.

Hepatobiliary function

- Capecitabine undergoes hepatic metabolism. In addition, participants on capecitabine may have temporary treatment-related elevation of transaminases which require interruption of treatment.
- Bilirubin <2 x ULN and AST or ALT < 5 x ULN is required for study entry. If bilirubin rises above this limit or AST or ALT more than doubles following the start of treatment and is above 2.5 x ULN during treatment, discuss with consultant as this may indicate disease progression. If treatment is to continue, refer to Table D2 for dose modifications.
- An isolated rise in transaminase above 2.5 x ULN or a doubling from the baseline level during treatment is likely to be treatment-related and capecitabine should be interrupted until recovery or local practice should be followed.

Table D2. Suggested oxaliplatin and capecitabine dose modifications for renal and hepatobiliary function

For cycle 1 of treatment:

<table>
<tr><th></th><th></th><th>Oxaliplatin dose</th><th>Capecitabine dose</th></tr>
<tr><td rowspan="3">Renal function</td><td>GFR ≥50 ml/min</td><td>full</td><td>full</td></tr>
<tr><td>GFR 30–49 ml/min</td><td>full</td><td>reduce by 25% of calculated dose</td></tr>
<tr><td>GFR <30 ml/min</td><td colspan="2">not eligible</td></tr>
<tr><td rowspan="2">Hepatic function</td><td>Bili < 2 x ULN <u>and</u> AST or ALT < 5 x ULN</td><td>full</td><td>full</td></tr>
<tr><td>Bili ≥2 x ULN <u>or</u>
AST or ALT≥5 x ULN</td><td colspan="2">not eligible</td></tr>
</table>

For subsequent cycles of treatment:

		Oxaliplatin dose	Capecitabine dose
**Renal function**	GFR ≥50 ml/min	full	full
	GFR 30–49 ml/min	full	reduce by 25% of dose given in previous cycle
	GFR <30 ml/min	withhold until recovery	
**Hepatic function**	Bili ≤2 x ULN and AST or ALT ≤2.5 x ULN	full	full
	AST or ALT >2x the baseline vaule and >2.5x ULN	Withhold until recovery to below double baseline value	
	Bili >2 x ULN	reduce by 50%* of previous dose	reduce by 50%* of previous dose

*the decision to treat with a bilirubin > 2 x ULN should be made by the treating clinician only after a full clinical assessment of the participant.

Respiratory

- As with other platinum drugs, rare cases of acute interstitial lung disease or lung fibrosis have been reported with oxaliplatin. In the case of unexplained respiratory symptoms or signs, oxaliplatin should be discontinued until further pulmonary investigations exclude an interstitial lung disease.

Allergic reactions to oxaliplatin

- Participants may develop acute hypersensitivity to oxaliplatin, usually only after several doses. During drug administration, the participant may develop rash, fever, swollen mouth or tongue, hypo- or hypertension and other signs/symptoms of hypersensitivity, although full-blown anaphylaxis is rare.
- If self-limiting hypersensitivity occurs, discontinue the infusion and treat with i.v. corticosteroid and antihistamine. The oxaliplatin infusion can be restarted on recovery, given over 6 hours. If hypersensitivity reoccurs again, the oxaliplatin should be permanently discontinued.
- After full recovery, the participant may continue with capecitabine alone.

Appendix E – Response evaluation criteria in solid tumours (RECIST)

Response to treatment will be assessed based on RECIST v1.1. A copy of the revised RECIST guideline is provided in the Investigator Site File and may also be obtained at:

http://www.eortc.be/recist/

Published date: January 2009.[38]

Appendix F – National Cancer Institute Common Terminology Criteria for Adverse Events (NCI-CTCAE)

Toxicities will be assessed based on the National Cancer Institute Common Terminology Criteria for Adverse Events (NCI-CTCAE) v4.0. A copy is provided in the Investigator Site File and may be obtained at:

http://evs.nci.nih.gov/ftp1/CTCAE/About.html

http://www.hrc.govt.nz/sites/default/files/CTCAE%20manual%20-%20DMCC.pdf

Published date: 28 May 2009.

Appendix G – Summaries of Product Characteristics

Up-to-date copies of the Summary of Product Characteristics for oxaliplatin and capecitabine are available by following the web link:

http://emc.medicines.org.uk/

For consistency, reference SPCs produced by named manufacturers are identified as follows:

Capecitabine : Roche Products Ltd

Oxaliplatin: Accord Healthcare Limited

Appendix H – Overall Treatment Utility (OTU) Definition

OTU is a novel clinical outcome measure incorporating objective and participant reported measures of anticancer efficacy, tolerability and acceptability of treatment, assessed 9 weeks post-randomisation and condensed into a simple 3-point score.

OTU may be regarded as asking the clinician: *"With the benefit of hindsight, are you glad you gave this treatment?"* and asking the participant: *"With the benefit of hindsight, are you glad you received it?"*: OTU is scored as good, intermediate or poor, corresponding to "yes", "uncertain/disagree" or "no" replies to these questions.

To score OTU, the participant is assessed 9 weeks after randomisation, using the following criteria:

1. **Is the treatment considered to have helped?**

   a. Scored as "YES" if all the following apply:
      - No evidence of radiological progression using RECIST
      - No other clinician-assessed evidence of cancer progression[1]
      - No major deterioration in Global QL[2]

   b. Scored as "NO" if any of the following apply:
      - Radiological progression using RECIST
      - Other clinician-assessed evidence of cancer progression
      - Major deterioration in Global QL

2. **Is the treatment tolerable and acceptable?**

   a. Scored as "YES" if all of the following apply:
      - No SAR or SUSAR definitely attributed to treatment
      - The patient's response to the question *"How much has your treatment interfered with your normal daily activities?"* is not "Very much" or "quite a bit".
      - The patient's response to question *"How worthwhile do you think your treatment has been?"* is not "Not at all"

   b. Scored as "NO" if any of the following apply:
      - SAR or SUSAR definitely attributed to treatment
      - The patient's response to the question *"How much has your treatment interfered with your normal daily activities?"* is "Very much" or "quite a bit"
      - Pthe patient's response to the question *"How worthwhile do you think your treatment has been?"* is "Not at all"

Scoring:

**Good OTU:**	Patient is alive and scores are "YES" for both 1 and 2.
**Intermediate OTU:**	Patient is alive and scores are "YES/NO" or "NO/YES".
**Poor OTU:**	Scores are "NO" for both 1 and 2, or patient has died.

[1] Clear clinical evidence of cancer progression which has not been confirmed radiologically.
[2] A drop of 16 or more points in EORTC QLQ-C30 Global QL Subscale

Appendix I - Safety monitoring plan

Study Title: **GO2**

Risks associated with trial interventions

☒ LOW ≡ Comparable to the risk of standard medical care

☐ MODERATE ≡ Somewhat higher than the risk of standard medical care

☐ HIGH ≡ Markedly higher than the risk of standard medical care

Justification: *Briefly justify the risk category selected and your conclusions below (where the table is completed in detail the detail need not be repeated, however a summary should be given):*

This trial involves the use of familiar drugs in line with standard practice.

What are the key risks related to therapeutic interventions you plan to monitor in this trial?		How will these risks be minimised?		
IMP/Intervention	Body system/Hazard	Activity	Frequency	Comments
Oxaliplatin	Vein pain during administration.	Recommend heat pad application during administration.	Day 1 per cycle (2 hour infusion)	
	Hypersensitivity reactions	Monitor during infusion. Dose modifications outlined in protocol.	Day 1 per cycle (2 hour infusion)	Full-blown anaphylaxis is rare
	Neurotoxicity, pulmonary interstitial lung disease (rare)	All participants seen by oncologist prior to each cycle to review toxicity and participant wellbeing.	Before each cycle	
Oxaliplatin and Capecitabine	Renal impairment, nausea, vomiting, diarrhoea, haematological changes	All participants seen by oncologist to review toxicity and FBC, U&Es and LFTs are checked prior to each cycle. Dose	Before each cycle	

		modifications for common side effects are outlined in the protocol, though sites may follow standard practice. Dose reductions in response to renal or hepatic impairment are mandated for cycle 1 but local practice may be followed for cycle 2 and subsequent cycles.		

Outline any other processes that have been put in place to mitigate risks to participant safety (e.g. IDMC, independent data review,...)

A data monitoring and ethics committee (DMEC) will be convened who will periodically (at least annually) review unblinded safety information. The DMEC will in light of these reports, have the authority to recommend trial closure to the Trial Steering Committee (TSC) should they have concerns over the safety or ethics of the trial. The TSC have the authority to recommend closure of the trial to the sponsor at any time.

Participant data will be entered onto a validated database and monitored for completeness and quality by the CTRU. Missing data will be chased until it is received, confirmed as not available, or the trial is at analysis. A validation check program will be incorporated into the database to verify the data, and discrepancy reports will be generated for resolution by the local investigator. Priority validations will be incorporated into the validation program to ensure that any discrepancies related to participant rights or the safety of participants are expedited to participating centres for resolution.

Appendix J - Definition of frailty

The definition of frailty is based on 9 domains assessed at baseline, using the comprehensive health assessment (CHA).

Domains assessed at baseline (CHA)	Tools used	Proposed cut off for impaired domain
**Weight loss**	How many Kg lost in the past 3 months<br><br>BMI	3kg or >5% body weight or<br><br>BMI <18.5
**Mobility**	Timed up and go test	>10 seconds or unable to complete test
**Falls**	G8 question	Has had 2 or more falls in the past 6 months
**Cognition**	G8 question	Mild or severe dementia diagnosis
**Function**	Nottingham ADL/IADL	One or more impairment in IADL or ADL
**Social**	Place of residence	Requires 24 hour care
**Mood**	EQ5D question (feelings today)<br><br>• Anxious or depressed: not/moderately/extremely	Extremely anxious/depressed
**Fatigue**	EORTC QLQC30 questions (not at all/ a little/quite a bit/ very much<br><br>• During the past week did you need to rest?<br>• During the past week were you tired?	Very much for either needing to rest or was tired<br><br>or<br><br>Quite a bit for both questions
**Polypharmacy**	Number of prescribed regular medications	5 or more
**9 domains**		

A participant is deemed frail if they have **impairment in two or more domains**. Further detail is given in the GO2 statistical analysis plan.
