# Supplementary material for: Efficacy of Reduced-Intensity Chemotherapy With Oxaliplatin and Capecitabine on Quality of Life and Cancer Control Among Older and Frail Patients With Advanced Gastroesophageal Cancer: The GO2 Phase 3 Randomized Clinical Trial
Source: JAMA Oncol. 2021 May 13;7(6):869–77. doi: 10.1001/jamaoncol.2021.0848 (PMC8120440; doi:10.1001/jamaoncol.2021.0848)
Supplement: Supplement 2. — Statistical Analysis Plan [file jamaoncol-e210848-s002.pdf]

ISRCTN 44687907

CLINICAL TRIALS RESEARCH UNIT (CTRU)
UNIVERSITY OF LEEDS

FINAL STATISTICAL ANALYSIS PLAN

GO2

v2.0

JANUARY 2019

VERSION CHANGES	
1.0 – 2.0	**General:**<br>• Trial Statistician updated to Alina Striha; Supervising Statistician updated to Helen Marshall; Senior Trial Coordinator updated to Sharon Ruddock; Delivery Lead updated to Helen Howard; Data Manager added - Eszter Katona.<br>• 100%, 80% and 60% amended to Levels A, B and C throughout.<br>• Randomisation A and B changed to the 'certain' randomisation and the 'uncertain' randomisation.<br><br>**1.1: Background**<br>• Text added to paragraph 4: In a feasibility study for this trial, "321GO", elderly and/or frail patients were randomised to receive 3, 2, or 1 drug treatment (epirubicin-oxaliplatin-capecitabine; oxaliplatin-capecitabine; or capecitabine) at 80% of the doses used in the standard EOxCap regimen (a.k.a. EOX), and showed that 2 drugs gave the best combination of tolerability and efficacy.<br><br>**1.3: Design**<br>• Chemotherapy vs. BSC comparison: Wording updated from 'The best supportive care arm will be used to consider if chemotherapy is worthwhile for participants in randomisation B and will compare BSC with 60% OxCap.' to 'The best supportive care (BSC) arm will be used to evaluate the impact of chemotherapy in patients where there is an uncertain benefit, by comparing BSC with Level C OxCap'.

- Chemotherapy intensity comparison: number of participants randomised to chemotherapy under the 4 way ‘uncertain’ randomisation prior to protocol v4 added.
- Minimisation factors added.

1.4: Sample size and expected accrual
- Length of recruitment period updated from 3 to 4 years.

1.5: Planned analyses
- Updated analysis timing to be final analysis will take place once the minimum required number of PFS events have occurred in each comparison in the certain benefit pathway, or when the most recently randomised, surviving participant has been followed up for 1 year post randomisation, whichever is reached sooner.
- Updated timing and method of data collection for the updated OS analysis to be following collection of extended survival data from sites approximately 1 year post randomisation of the final participant.
- Removed details of the updated QAS analysis as this will not be performed given only extended survival data will be collected i.e. extended QoL data will not be collected.

2.1: Chemotherapy intensity comparison
2.1.1: Primary end point
- Added that a test for heterogeneity will be performed between the three levels and if there is a difference, a test for trend will be carried out across the 3 arms.
- Amended the null hypothesis to be that the lower-dose treatment (i.e. Level B or Level C) is not non-inferior in terms of PFS when compared to Level A, rather than inferior, and added detail.
- Added further detail around the non-inferiority margin.

2.1.2: Derivation of primary endpoint
- Added further details of censoring to allow for follow-up visits taking place after 52 weeks.
- Added that radiological progression will also be included for non-RECIST evaluable at baseline participants.

2.1.3: Secondary endpoints
- Added: The assessment of PFS and OS by Overall Treatment Utility (OTU) status at 9 weeks will not incorporate a comparison between treatment groups. The comparison to be made will be between the three OTU groups (good, intermediate, poor), as given in Appendix 1, at 9 weeks post-randomisation. The null hypothesis is that there is no difference between the OTU status groups in terms of PFS and OS. The alternative hypothesis is that there is a difference, with increasing benefit (superiority) with increasing value of OTU anticipated. The test will be two-sided with a 5% significance level. Although this is not an endpoint it is deemed an important analysis.

2.1.4: Derivation of secondary endpoints
- Added details of reduction in QoL data collection and how this will be addressed in the analysis.
- Participant reported fatigue: Added ‘All time points will be used in multi-level repeated measures models to give treatment effect estimates at each time point. However, 9 weeks will be used as the primary assessment time point. This will allow exploration of differing missing data assumptions to determine how much this may affect the interpretation of the results.’
- Time to deterioration of participant reported fatigue: Added **‘**A sensitivity analysis will be performed using a magnitude of medium deterioration.’
- Added: For the analysis of PFS and OS by OTU status at 9 weeks, PFS is calculated from the date of the 9 week LHA where OTU is assessed, to first documented evidence of disease progression or death. Participants who have not progressed/died at the time of analysis will be censored at the last date they were known to be alive and progression-free. Participants who progress or die prior to their OTU assessment will be included as having had an event at time 0. OS is calculated from the date of the 9 week LHA where OTU is assessed, to the date of death. Participants who have not died at the time of analysis will be censored at the last date they were known to be alive. Participants who die prior to their OTU assessment will be included as having had an event at time 0.These analyses will include all participants with an OTU assessment.
- Quality adjusted survival: removed ‘and will be defined in a separate analysis plan’ as details of the planned analyses are now included in this analysis plan.
- Overall survival: Updated according to new method of data collection (i.e. from sites rather than routine data) for deaths not within 1 year.
- Toxicity: Added: Toxicity will be reported/summarised in parallel with patient reported toxicity to allow a visual comparison of concordance.

2.3: Missing data
- Updated: Where the death / progression date is unknown, however a month and year is known, the patient will be assumed to have died / progressed on the 15th of the month with the exception of the following situations: The imputed death/progression date will be cross checked with other patient dates to make sure that the imputed death/progression date is not before any dates when the patient is known to be still alive/ not progressed, in which

	case the date midway between the date where they were known to be still alive/ not progressed and the end of the month will be used. If it is known that the death/ progression date is before a particular date, the date used will be half way between the earliest and latest possible dates when death/ progression could have occurred i.e. if it is known that the participant died/progressed before a certain date e.g. 14th we would use the date half way between 1st and 14th. Sensitivity analyses will be conducted using the earliest and latest possible day of the month if there are >5% of partially missing dates.<br>• Added: 'If a participant has a missing questionnaire(s) and has not experienced a deterioration of participant reported fatigue, they will be censored at their last questionnaire completion date. If a participant dies following a missing questionnaire their deterioration of fatigue will also be censored at their last questionnaire completion date.'<br>• Clarified details of sensitivity analyses to be applied for missing questionnaires in relation to deterioration of participant reported fatigue.<br>• Added details of assumptions to be made if missing data exist for the participant-reported components of OTU and also relevant sensitivity analyses.<br><br>**3: Populations**<br>• Added clarification that for the superiority endpoints, a per-protocol analysis will only be performed where a significant number of participants are not in the PP population (>5%).<br><br>**3.2: Per protocol population**<br>• Added 'Participants who were subsequently found not to have histologically or cytologically confirmed carcinoma of the oesophagus, GO-junction or stomach' to list of major protocol violators.<br>• Included updated criteria for hepatic function (Protocol Version 6.0 onwards).<br><br>**3.3: Safety Population**<br>• Updated how we will deal with participants who do not fall within the 10% boundaries from being monitored by the DMEC to 'for any instances where a participant's starting dose does not fall within +/-10% of one of the treatment arms (e.g. if the dose lies between 66% and 72%, or between 88% and 90%), the absolute cut-points 70% and 90% will be used to determine which arm the participant will be included in i.e. participants receiving ≤69.9% of the relevant full dose will be included in the Level C arm, participants receiving 70-89.9% of the relevant full dose will be included in the Level B arm, whilst participants receiving ≥90% of the relevant full dose will be included in the Level A arm.'<br><br>**3.4: RECIST evaluable population**<br>• Added 'If there are discrepancies between the baseline and follow-up CRFs as to whether or not the participant was RECIST evaluable at baseline, the information provided at follow-up will be used e.g. if the 9-week CRF states that the participant was not RECIST evaluable at baseline, this information will be assumed to be correct.'<br><br>**5: Data Analysis**<br>• Clarification throughout that analyses adjusting or stratifying for the minimisation factors will not adjust/stratify for centre.<br><br>**5.2.1: Study summary**<br>**Relative dose intensity**<br>• Clarified that RDI will be calculated from the date treatment started to 9 and 18 weeks later and that patients who have died or stopped treatment due to progression within 9/18 weeks will be censored at their date of death/progression.<br><br>**5.2.4: Secondary endpoints**<br>• Added 'All data received from QoL questionnaires will be included in the final analysis. In addition a plot will be presented to show how closely the data adheres to the QoL time points. QoL questionnaires within +/-4 weeks of the specified time points will be included in the compliance level for the analyses.'<br>• Overall treatment utility - added that other covariates which are potentially prognostic of outcome will be included in a secondary analysis. Included that sensitivity analyses may also be performed.<br>• Section added: PFS and OS by OTU status at 9 weeks (superiority).<br>Progression-free survival and overall survival curves will be calculated using the Kaplan-Meier method and PFS/OS estimates as appropriate will be presented by OTU status at 9 weeks post-randomisation. A log-rank test will be used to compare progression-free survival and overall survival between the OTU status groups, adjusting for treatment group.<br>The Cox proportional hazards model (if appropriate), adjusting for relevant factors as appropriate, will also be used to compare progression-free survival and overall survival between the OTU status groups. Factors that could be adjusted for as appropriate include treatment received, minimisation factors (excluding centre) and other important prognostic factors identified, and a treatment by OTU status interaction. OTU status and other covariate estimates, standard errors, hazard ratios, 95% confidence intervals, as well as p-values will be presented for each model investigated.<br>This analysis will compare OTU status groups.<br>• Quality Adjusted Survival - included details of the planned analyses rather than stating that they will be defined in a separate analysis plan.

	• Frailty Analyses: added that sensitivity analyses may be performed.<br><br>**Further exploratory analyses**<br>• Subgroup analyses and exploratory prognostic factor analyses sections updated to remove 'specific details relating to these analyses will be documented prior to analyses being undertaken' and include 'specific covariates of interest, additional to the clinical randomisation factors, will include those listed in Appendix 3'.<br>• Added an exploratory analysis assessing the impact of baseline frailty on 9-week treatment tolerability.<br><br>**6: Reporting and Dissemination of the Results**<br>• Updated analysis timing to be final analysis will take place once the minimum required number of PFS events have occurred in each comparison in the certain benefit pathway, or when the most recently randomised, surviving participant has been followed up for 1 year post randomisation, whichever is reached sooner.<br>• Updated timing of the updated OS analysis to be following collection of extended survival data from sites approximately 1 year post randomisation of the final participant.<br>• Removed details of the updated QAS analysis as this will not be performed given only extended survival data will be collected i.e. extended QoL data will not be collected.<br><br>**Appendix 1 – Overall treatment utility**<br>• Updated to correspond with the updated appendix in Protocol Version 7.0.<br><br>**Appendix 3 – Covariates prognostic of outcome**<br>• Appendix added to detail additional covariates to include in multivariate analyses.

Trial Statistician:	Alina Striha
Supervising statistician:	Helen Marshall
Senior Trial Coordinator:	Sharon Ruddock
Delivery lead:	Helen Howard
Scientific lead:	Fiona Collinson
Data Manager:	Eszter Katona
Chief Investigators:	Professor Matthew Seymour and Dr Peter Hall

Table of Contents

1. Introduction 6
1.1 Background 6
1.2 Aims 6
1.3 Design 6
1.4 Sample size and accrual 7
1.5 Planned analyses 10
2. Endpoints 10
2.1 Chemotherapy intensity comparison 10
2.1.1 Primary endpoint 10
2.1.2 Derivation of primary endpoint 10
2.1.3 Secondary endpoints 11
2.1.4 Derivation of secondary endpoints 12
2.2 Chemotherapy vs. BSC comparison (exploratory) 13
2.2.1 Primary endpoint 13
2.2.2 Secondary endpoints 13
2.2.3 Derivation of primary and secondary endpoints 13
2.3 Missing data 13
3. Populations 15
3.1 Intention-to-treat population (ITT) 15
3.2 Per-protocol population (PP) 15
3.3 Safety population 16
3.4 RECIST evaluable population 16
4. Data Handling 16
4.1 Data monitoring 16
4.2 Data validation 17
5. Data Analysis 17
5.1 General calculations 17
5.2 Analysis 17
5.2.1 Study summary 17
5.2.2 Baseline characteristics 18
Chemotherapy intensity comparison 18
5.2.3 Primary endpoint: Progression-free survival (non-inferiority) 18
5.2.4 Secondary endpoints 18
Chemotherapy vs. BSC comparison (exploratory) 22
5.2.5 Primary endpoint: overall survival (superiority) 22
5.2.6 Secondary endpoints: participant reported fatigue and QoL (superiority) 22
6. Reporting and Dissemination of the Results 23
7. References 23
8. Appendices 25
Appendix 1 – Overall Treatment Utility (OTU) Definition 25
Appendix 2 – Definition of frailty 26
Appendix 3 – Covariates prognostic of outcome 27

1. Introduction

1.1 Background

Gastric and oesophageal (GO) cancer causes 13,000 deaths/year in the UK, at a median age of 77 years.[1] The peak age of diagnosis is becoming older,[2] and the diagnosis commonly follows a period of nutritional dysfunction. As a consequence, many GO cancer patients are frail, with co-morbidities and reduced performance status (PS).

Recent years have seen a welcome shift in UK cancer management: all patients with malignancy, including the frail and elderly, are now managed by multidisciplinary teams (MDTs) with site-specialised oncology expertise. Consequently, most patients with advanced GO cancer are considered for, and many receive, chemotherapy as part of their palliative management. A report published by the Department of Health in conjunction with MacMillan and Age UK highlighted the lack of standardised care for older patients with cancer. It pointed to a need to identify appropriate methods for assessing patients for prognosis and their potential to benefit from evidence-based treatment.[3]

Despite efforts to make the eligibility criteria for trials inclusive, there is a conspicuous mis-match between the age of patients with advanced GO cancer in the population (median over 75 years) and the populations recruited to randomised controlled trials (RCTs) such as REAL2[4] (median 63 years). There is a similar but less measurable mismatch in frailty, performance status and co-morbidity. This leaves uncertainty in both patient selection and choice of dose/regimen.

It is now well recognized that age alone is no bar to benefit from chemotherapy. But age-related changes in pharmacokinetics and pharmacodynamics can lead to higher toxicity when elderly patients are treated with doses established in younger or fitter patients.[5,6] Furthermore, the acceptability of complex treatments can be lower in this population.[7] In a feasibility study for this trial, “321GO”, elderly and/or frail patients were randomised to receive 3, 2, or 1 drug treatment (epirubicin-oxaliplatin-capecitabine; oxaliplatin-capecitabine; or capecitabine) at 80% of the doses used in the standard EOxCap regimen (a.k.a. EOX), and showed that 2 drugs gave the best combination of tolerability and efficacy. A large randomised controlled trial is now required for patients who are unfit for full-dose 3-drug chemotherapy (e.g. EOX, ECF), providing evidence to guide treatment.

1.2 Aims

GO2 aims to establish the optimum dose-intensity of 2-drug palliative chemotherapy for advanced GO cancer in frail/elderly patients, to achieve the best balance of cancer control, toxicity, patient acceptability and quality of life. It will also help establish pre-treatment patient characteristics in individual patients that predict for better or worse outcomes with chemotherapy at different dose intensities. In an exploratory analysis, GO2 also aims to address whether chemotherapy improves overall survival in frail/elderly patients for whom there is substantial uncertainty about the role of chemotherapy.

1.3 Design

GO2 is a phase III, randomised, multi-centre, prospective, controlled, open label, non-inferiority trial comparing three dose levels of combination chemotherapy - oxaliplatin and capecitabine (OxCap). The three doses tested, Levels A, B and C, represent 100%, 80% and 60% respectively of the doses of these drugs as used in standard-dose EOX. Eligible patients are those not fit for full dose 3-drug chemotherapy, but suitable for reduced intensity chemotherapy. A separate randomisation compares OxCap with best supportive care in patients for whom there is substantial uncertainty about the suitability of chemotherapy. Participants must provide informed consent to be randomised into the trial. The trial is in the setting of the UK National Health Service.

The minimisation factors are as follows:

- Centre
- Age (≥75 or <75 years)
- Distant metastases (yes or no)
- Histology (squamous or other)
- Dose reduction required due to renal or hepatic function (yes or no) – (see Section 11.3 and Table D2 in the GO2 protocol)
- Planned use of trastuzumab (yes or no/not yet decided)
- WHO Performance status (0-1 or 2 or >2).

After discussions with the TMG, investigators at sites and the DMEC, a decision was made in October 2014 to amend the uncertain randomisation from a 4-way randomisation to a 2-way randomisation (protocol version 4.0). This was based on recruitment issues seen, and concerns around finding patients who are suitable for any dose level of OxCap and BSC (options in the 4-way randomisation) and also willing to be randomly assigned to one of the 4 options.

Comparisons

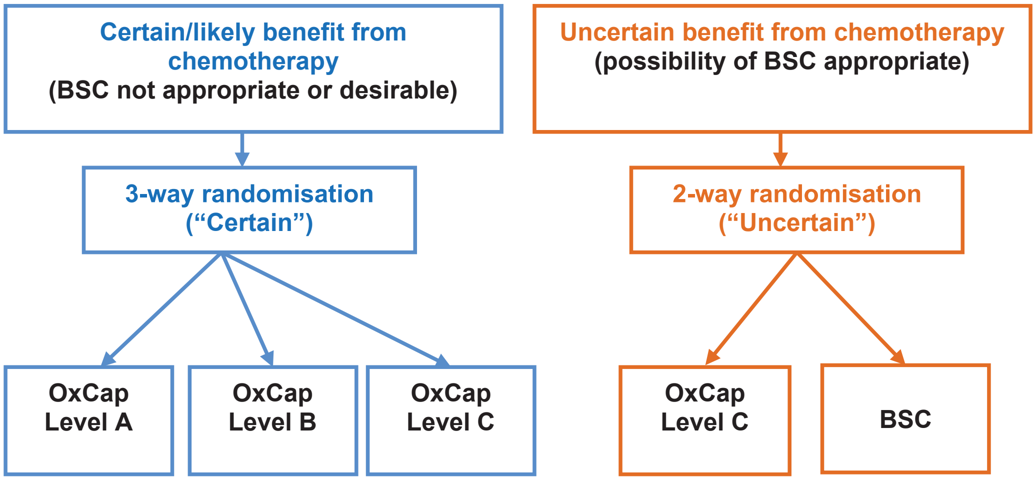

Participants entered into the uncertain randomisation prior to Protocol Version 4.0 were randomised 1:1:1:1 to Level A, Level B, Level C or BSC.

Chemotherapy intensity comparison
In order to determine the optimal chemotherapy dose intensity in GO2, the different intensity chemotherapy arms from the certain/likely benefit randomisation will be compared. Level C OxCap and Level B OxCap will be compared to Level A OxCap (i.e. two different comparisons).

Participants entered into the uncertain benefit randomisation prior to Protocol Version 4.0 were randomised to Level A OxCap, Level B OxCap, Level C OxCap or BSC. Those who were randomised to one of the chemotherapy arms will be included in the chemotherapy intensity question (2 participants). Participants entered into the uncertain randomisation under Protocol Version 4.0 onwards will not be included in the chemotherapy intensity comparison.

Chemotherapy vs. BSC comparison
The best supportive care (BSC) arm will be used to evaluate the impact of chemotherapy in patients where there is an uncertain benefit, by comparing BSC with Level C OxCap. Analysis of this comparison will be exploratory in nature. Participants entered into the uncertain randomisation prior to protocol version 4.0 who were randomised to Level C OxCap or BSC will be included in the chemotherapy vs. BSC comparison. Participants entered into the uncertain pathway prior to Protocol Version 4.0 who were randomised to Level A OxCap or Level B OxCap will not be included in this comparison.

1.4 Sample size and accrual
The initial planned length of the recruitment period for GO2 was 3 years, with no fixed sample size, but an aim to recruit a minimum of 500 participants to the certain pathway, and an additional 60 participants to the uncertain pathway during this recruitment period. Based on observed accrual rates after 2 years of accrual, the duration of accrual was amended to 4 years to ensure recruitment of at least 500 certain pathway patients.

Primary outcome based on chemotherapy intensity comparison: Progression-free survival
Initial analysis of data from 321GO, based on a median follow-up of 5.9 months (IQR 2.7-9.5 months), suggests that the overall median PFS for patients with advanced GO cancer who are not fit for full dose EOX but suitable for reduced dose chemotherapy is 132 days (95% CI 84 to 169 days).

Although the 95% confidence intervals around the median PFS estimate are wide, and the estimate could

change with further follow-up, we are confident that the true rate will be nearer to 132 days (4.4 months) than the limits of the confidence intervals. This is based on a comparison of the FOCUS2, FOCUS, REAL1 and REAL2 trials and the PFS ratio of 'non-fit' to 'fit' patients in the different populations as follows:

Table 1. Median PFS/FFS estimates in FOCUS2, FOCUS, REAL1 and REAL2 trials

Trial	Patient population	Median PFS/FFS estimate
FOCUS2[8]	Colorectal cancer 'non-fit' patients	Median PFS was 3.5, 5.8, 5.2 and 5.8 months in the FU, OxFU, Cap and OxCap groups respectively; a reasonable estimate for all FOCUS2 patients therefore is 5 months
FOCUS[9]	Colorectal cancer 'fit' patients	Median PFS was 6.3, 8.5 and 8.7 months in the fluorouracil, irinotecan + fluorouracil and oxaliplatin + fluorouracil first line therapy groups respectively; a reasonable estimate for all first line FOCUS patients therefore is 8 months
The ratio of non-fit:fit patients with colorectal cancer is therefore 5:8 i.e. 63%		
REAL1[7]	Oesophagogastric cancer 'fit' patients	Median FFS was 7 months in both the ECF and MCF groups
REAL2[4]	Oesophagogastric cancer 'fit' patients	Median PFS was 6.2, 6.7, 6.5 and 7.0 months in the ECF, ECX, EOF and EOX groups respectively; a reasonable estimate for all REAL2 patients therefore is 6.5 months

Therefore applying the same ratio of 'non-fit' to 'fit' patients seen in colorectal cancer to GO cancer, we can estimate median PFS for GO2 participants to be around 4-4.4 months using the REAL1 and REAL2 trials as the point of reference.

To determine an acceptable non-inferiority margin for the lower-dose treatments in GO2 (Level B OxCap and Level C OxCap), extensive consultation has been undertaken with clinical groups, including the Upper GI Clinical Studies Group, 321GO investigators and user groups:

- Clinical feedback has suggested a median PFS detriment of no more than 1 month in absolute terms (i.e. from 132 to 102 days), or a hazard ratio of around 1.25 in relative terms.
- The over-riding opinion from our patient and public involvement (PPI) representatives is that the optimal balance between survival and QoL will vary widely between individual patients, making it difficult to reach a consensus; however they have indicated that they would accept a larger loss in efficacy than clinicians in return for gains in QoL. Most considered a reduction of up to 6 weeks (42 days) in median PFS to be acceptable. They commented that *"some information is better than none when patients are faced with treatment decisions"* and suggested that we might concentrate on what non-inferiority margin was feasible to observe rather than setting an arbitrary or unachievable target.

Given these differences, and the anticipated recruitment rate in this population, rather than specifying an absolute target sample size, it is more appropriate to specify a minimum together with a target length of recruitment, with the aim to recruit as many participants as possible in this time. Recruiting more than the minimum number of participants will accommodate the uncertainty in the underlying sample size assumptions and will reduce the variability of treatment effect estimates in the analysis.

The table below provides a range of estimates showing what non-inferiority margins can be achieved for differing sample sizes. The sample sizes considered relate to the certain pathway in the trial; the uncertain pathway is considered separately.

- GO2 aims to recruit a minimum of 500 participants to the certain pathway (167 per chemotherapy dose intensity – Level A, Level B, Level C); this will permit a non-inferiority margin of 34 days median PFS in absolute terms, or HR non-inferiority boundary = 1.34 (80% power; 1-sided 5% significance level, based upon a 1-sided log rank test assuming all participants are followed up for 1 year and that the hazard ratio is constant).
- If recruitment into GO2 reaches our upper estimate of 750 participants in the certain pathway (250 per dose intensity), this will permit a non-inferiority margin of 28 days median PFS in absolute terms, or HR non-inferiority boundary = 1.27, with the same power.
- If recruitment proves more challenging than expected over the extended recruitment period, a total of 300 participants in the certain pathway (100 per dose intensity) would still allow exclusion of a PFS detriment of 42 days, in line with the consumer view.

These estimates have not accounted for any losses to follow-up as drop-out is assumed to be minimal given the short survival expectancy of these patients and their high dependency on medical services (zero drop-out was noted in 321GO). Losses will have a small impact on the non-inferiority margin; for example, with 500 participants, a 5% drop-out rate would result in an increase in the non-inferiority margin from 34 to 35 days and the HR boundary from 1.34 to 1.35.

Table 2. Anticipated non-inferiority margins based on differing sample sizes

Recruitment length	Number of participants/dose intensity; total denotes certain pathway only, assuming no dropout	Number of PFS events for each comparison	HR non-inferiority boundary	Reduction in median PFS (days) (=non-inferiority margin)
4 years	167 (500 in total)	284	1.34	34
	184 (550 in total)	314	1.32	32
	200 (600 in total)	341	1.31	31
	217 (650 in total)	370	1.30	30
	235 (700 in total)	401	1.28	29
	250 (750 in total)	427	1.27	28

Every effort will be made to recruit the minimum of 500 participants, therefore non-inferiority margins have not been calculated for smaller sample sizes.

Power for Quality of Life

The primary endpoint in GO2 of the chemotherapy intensity comparison is progression-free survival therefore no formal power calculation has been performed for the quality of life outcomes. However, using the operational definitions by Cohen[10], where a small effect size is defined to be between 0.2 and 0.5, a moderate effect size is defined to be between 0.5 and 0.8 and a large effect size is defined to be >0.8, a sample size of 500 participants in the certain pathway (167 per dose intensity) would give us power to detect an effect size of 0.307 with 80% power and a 2-sided 5% significance level, whilst a sample size of 750 participants in the certain pathway (250 per dose intensity) would give power to detect an effect size of 0.251.

However it is acknowledged that this does not take into consideration questionnaire non-compliance. In 321GO, where questionnaires were administered by research nurses in clinic, follow-up compliance was approximately 70%. Assuming this compliance for GO2 gives us power to detect effect sizes of 0.368 and 0.300 when recruiting 500 and 750 participants to chemotherapy respectively. Therefore given Cohen's definitions, it is expected that we will be able to detect small effect sizes i.e. small improvements in quality of life between the different dose intensities.

Recruitment

We aim to recruit a minimum of 500 participants to the certain pathway over an extended recruitment period of 4 years. Based on 321GO, the initial recruitment target of 500 participants over 3 years was felt to be achievable. However, the minimum target of 500 participants does not appear likely to be met within 3 years, therefore options with regards to continuing recruitment beyond three years were reviewed in discussion with the TSC. A decision was made in December 2015 to extend the recruitment phase for 1 year. Funding will be managed by streamlining QoL collection, removing the short quality of life questionnaire at the end of each cycle and the weekly EQ-VAS.

Primary outcome based on chemotherapy vs. BSC comparison (exploratory): Overall survival

The inclusion of a BSC arm is exploratory; in order to estimate the outcome in the BSC arm and compare it against chemotherapy we would need at least 30 participants in this arm.[11]

Historical trials that compared chemotherapy with best supportive care (BSC) were summarised by Wagner et al. in their systematic review and meta-analysis[12] (see protocol for further detail). These trials demonstrated an increase in median survival of around 6 months with the addition of chemotherapy to BSC, from around 3 months in the BSC arms to around 9 months in the treatment arms.

Therefore, although the emphasis of the chemotherapy vs. BSC comparison in GO2 is exploratory, with 30 participants in the BSC arm we calculate there to be sufficient power (80%) to detect a clinically relevant and justifiable hazard ratio of 0.43 (using a 5% 2-sided significance level) for overall survival based on a median overall survival of 3 months in the BSC arm. This corresponds to a median overall survival of 7 months in the Level C OxCap arm.

Recruitment into the uncertain benefit decision pathway will end after the inclusion of 60 participants or the completion of four years of recruitment. An additional 60 participants will therefore be required overall in the trial – this adds 12% to the required sample size. As the chemotherapy vs. BSC comparison is exploratory, the uncertain benefit decision pathway may be stopped early or otherwise adapted on the advice of the DMEC and TSC. Decisions will be based on recruitment feasibility, revised power calculations or emerging evidence of harm.

1.5 Planned analyses

A DMEC will be set up to meet at least annually to independently review interim efficacy, safety and recruitment data. Detailed un-blinded reports will be prepared by the CTRU for the DMEC at approximately 12-monthly intervals, dependent upon recruitment rates and the number of participants in the trial.

No formal interim analyses are planned so no statistical testing will take place until final analysis. Final analysis will take place once the minimum required number of PFS events have occurred (as specified in the sample size calculation): 284 in each comparison in the certain benefit pathway (Level B vs. Level A and Level C vs. Level A); or when the most recently randomised, surviving participant has been followed up for 1 year post randomisation, whichever is reached sooner. A further updated analysis of overall survival will be performed following collection of extended survival data from sites approximately 1 year post randomisation of the final participant.

2. Endpoints

2.1 Chemotherapy intensity comparison

2.1.1 Primary endpoint

The primary endpoint for the chemotherapy intensity comparison is progression-free survival.

Analysis of PFS will be based on the 90% confidence interval (CI) (one-sided type I error rate of 5%, corrected for multiplicity[13]) of the hazard ratio (HR); the 90% CI of the difference in PFS at 4 months, and other fixed time-points as necessary, will also be presented to aid interpretation. We will look at non-inferiority (with the same margin of non-inferiority) of both Level B OxCap and Level C OxCap compared with Level A OxCap i.e. two separate comparisons. A test for heterogeneity will be performed between the three levels and if there is a difference, a test for trend will be carried out across the 3 arms.

The null hypothesis to be investigated in each case is that the lower-dose treatment (i.e. Level B or Level C) is not non-inferior in terms of PFS when compared to Level A i.e. the upper limit of the CI is beyond the non-inferiority margin. The alternative hypothesis is that the lower-dose treatment is non-inferior to Level A in terms of PFS.

There are differing opinions across groups in what loss in efficacy would be deemed acceptable to be able to claim non-inferiority; the decision on the non-inferiority margin to be used for the analysis of primacy is based on expert clinician opinion (see Section 1.4: Sample size and accrual). The upper limit of the (multiplicity corrected) 90% CI around the HR, for each comparison, will therefore be compared with the non-inferiority margin of HR = 1.34, which is equivalent to a reduction of 34 days in median PFS compared to the Level A arm. If it is below this margin for either comparison, then the result will be taken as evidence that Level B OxCap or Level C OxCap (depending upon the comparison) is non-inferior to Level A OxCap. If the upper limit is above the non-inferiority margin, then non-inferiority will not have been demonstrated. Given the lack of consensus however around the non-inferiority margin, sensitivity analyses for the choice of non-inferiority margin will be carried out.

2.1.2 Derivation of primary endpoint

Progression-free survival is defined as the time from randomisation to first documented evidence of disease progression or death from any cause. This can be clinical or radiological progression; for RECIST evaluable disease, this will be radiological progression by RECIST principles. Participants who do not progress or die

will be censored at the last date they were known to be alive and progression-free. Details on progression and deaths will be reported by sites up to 1 year from the date of randomisation. Participants with no reported PFS event during their 1-year follow-up period will be censored at their last follow-up date where they were known to be alive and progression-free. Deaths without progressions and progressions will be included up to 56 weeks to allow for the timing of the 52-week follow-up assessment to be slightly over the specified 52-week time-period.

Calculation of patients' progression-free survival time will be performed using SAS after the data has been downloaded from the database. Survival time will be calculated in days and survival estimates presented in months (or other appropriate intervals), where one month is defined as time in days / 30.44.

2.1.3 Secondary endpoints

For the secondary endpoints, we will compare both Level B OxCap and Level C OxCap with Level A OxCap i.e. two separate comparisons.

The following endpoints are assessed for superiority of the lower doses of OxCap (i.e. Level B or Level C, depending on the comparison) against Level A OxCap, with respect to each endpoint. In each case, the null hypothesis is that there is no difference between Level B OxCap and Level A OxCap, or Level C OxCap and Level A OxCap, depending on the comparison, in terms of the relative endpoint. The alternative hypotheses are that there is a difference, with superiority of the lower doses of OxCap (i.e. Level B or Level C) anticipated. Hypothesis testing is two-sided for superiority endpoints, with a 5% significance level.

- Participant reported fatigue
- Time to deterioration of participant reported fatigue
- Overall Treatment Utility
- QoL & symptoms
- Quality adjusted survival

The null hypotheses for the following endpoints are that the lower doses of OxCap (i.e. Level B or Level C) are not non-inferior to the Level A OxCap arm with respect to each endpoint. The alternative hypotheses are that the Level B OxCap or Level C OxCap arm, depending on the comparison, is non-inferior to the Level A OxCap arm with respect to each endpoint. Hypothesis testing is one-sided for non-inferiority endpoints, with a 5% significance level. Non-inferiority margins have not been pre-specified for these analyses. The level of non-inferiority that can be attained for each endpoint will be determined by the upper (OS – hazard ratio) or lower (best response – odds ratio) limit of the corresponding 90% confidence interval. For example, if the upper limit of the confidence interval of the OS hazard ratio (Level B vs. Level A) is 1.1, Level B OxCap will have been shown to be non-inferior to Level A OxCap at a HR of 1.1. Level B OxCap can be claimed to be non-inferior to Level A OxCap if a HR of 1.1 is acceptable (i.e. if it would be acceptable that patients are 10% more likely to die on the Level B OxCap arm). The results of these analyses will be interpreted through discussion with the TMG and patient representatives at the time of final analysis.

- Overall survival (OS)
- Best response

The following endpoint will not be subjected to any formal statistical testing and hence no hypotheses have been proposed.

- Toxicity

The assessment of PFS and OS by Overall Treatment Utility (OTU) status at 9 weeks will not incorporate a comparison between treatment groups. The comparison to be made will be between the three OTU groups (good, intermediate, poor), as given in Appendix 1, at 9 weeks post-randomisation. The null hypothesis is that there is no difference between the OTU status groups in terms of PFS and OS. The alternative hypothesis is that there is a difference, with increasing benefit (superiority) with increasing value of OTU anticipated. The test will be two-sided with a 5% significance level. Although this is not an endpoint it is deemed an important analysis.

2.1.4 Derivation of secondary endpoints

From Protocol Version 6.0, QoL data collection was reduced to those time points that correspond with trial follow up visits (i.e. at baseline and at 9, 18, 27, 36 and 52 weeks). The intermediate QoL time points collected every week whilst on chemotherapy until week 18 (using the EQ-VAS weekly and the short QoL questionnaire at the end of each cycle) were no longer collected.

The aim of the frequency of QoL data collection during the treatment period was to observe changes in QoL that occur during chemotherapy cycles within patients and between treatment groups that may not be detected if QoL was collected less frequently. This was in particular to inform the time to deterioration of participant reported fatigue and the quality adjusted survival analysis; weekly EQ-VAS and 3-weekly short QoL assessments aimed to enable these endpoints to be more sensitive as QoL/fatigue was being assessed more regularly and at times (i.e. whilst on chemotherapy) when greater changes may possibly occur.

All QoL data collected will be included in the analyses, including the discontinued time points detailed above. However, for participants randomised from Protocol Version 6.0 onwards any deterioration in fatigue will not be picked up until the 9 week LHA. The number of participants with the reduced QoL data collection will be given to aid interpretation.

- **Participant reported fatigue** is based on the QLQ-C30 v3.0 fatigue component, taken from the Comprehensive Health Assessment (CHA) completed at baseline and the Limited Health Assessment (LHA) completed at 9 weeks post-randomisation (for participants randomised to OxCap), and a more frequent short follow-up QoL questionnaire, completed up to 52 weeks post-randomisation. Scoring of the QLQ-C30 v3.0 fatigue component will be according to the EORTC QLQ-C30 scoring manual.[14] All time points will be used in multi-level repeated measures models to give treatment effect estimates at each time point. However, 9 weeks will be used as the primary assessment time point. This will allow exploration of differing missing data assumptions to determine how much this may affect the interpretation of the results.

- **Time to deterioration of participant reported fatigue** is defined as the time from randomisation to a large deterioration (defined by Cocks et al[15] as a difference of <-15 points) of QLQ-C30 (v3.0) fatigue as compared from participant's baseline fatigue score. A sensitivity analysis will be performed using a magnitude of medium deterioration. Participants who have died within 1 year of randomisation without experiencing a large deterioration of fatigue will be considered as having a competing-risk event at their date of death. Participants who do not experience a large deterioration of fatigue but are not known to have died within 1 year of randomisation will be censored at their last questionnaire completion date.

- **Overall treatment utility (OTU)** will be calculated as per Appendix 1 at 9 weeks post randomisation.

- For the analysis of **PFS and OS by OTU status** at 9 weeks, PFS is calculated from the date of the 9 week LHA where OTU is assessed, to first documented evidence of disease progression or death. Participants who have not progressed/died at the time of analysis will be censored at the last date they were known to be alive and progression-free. Participants who progress or die prior to their OTU assessment will be included as having had an event at time 0. OS is calculated from the date of the 9 week LHA where OTU is assessed, to the date of death. Participants who have not died at the time of analysis will be censored at the last date they were known to be alive. Participants who die prior to their OTU assessment will be included as having had an event at time 0. These analyses will include all participants with an OTU assessment. Although this is not an endpoint it is deemed an important analysis.

- **QoL & symptoms** are based on a Comprehensive Health Assessment (CHA) completed at baseline and a Limited Health Assessment (LHA) completed at 9 weeks post-randomisation (for participants randomised to OxCap), and more frequent short follow-up QoL questionnaires. Appropriate scoring manuals will be used for the different components, including the QLQ-C30 scoring manual[14] for both the QLQ-C30 and QLQ-OG25 components and the EQ-5D-3L user guide[16] for the EQ-5D components. The chemotherapy side effects questions are found on the LHA completed at 9 weeks post-randomisation and will be summarised descriptively.

- **Quality adjusted survival** will use the EQ-VAS to weight overall survival based on participant preferences. This analysis will be performed by Dr Peter Hall.

- **Overall survival** is defined as the time from randomisation to death from any cause. Participants who are not known to have died will be censored at the last date they were known to be alive. Deaths will be reported by sites during each participants' 1 year follow-up period, after which date of death or 'last date

known to be alive' will be requested from site for those participants alive at the end of their 1 year follow up period. These extended survival data will be collected approximately 1 year after randomisation of the last participant for an updated OS analysis. Patients with full withdrawals will be censored at the last date known to be alive.

- For the **best response** endpoint, the population of participants with disease evaluable by RECIST criteria will be used and for these participants, a CT scan at 9 and 18 weeks, and as clinically indicated thereafter whilst on chemotherapy, is requested. Best response is defined as the proportion of participants with each best response (i.e. complete response, partial response, stable disease or progressive disease) within 1 year of randomisation.

- **Toxicity** will be recorded based on serious adverse events (SAEs), suspected unexpected serious adverse reactions (SUSARs) and adverse reactions, as graded by CTCAEv4.0, at each chemotherapy cycle and follow-up assessment. Toxicity will be reported/summarised in parallel with patient reported toxicity to allow a visual comparison of concordance.

The impact of baseline **frailty** on outcomes and treatment effect will be assessed for the progression-free survival, overall survival, overall treatment utility, QoL & symptoms and toxicity endpoints. Frailty is defined using the CHA completed at baseline, as given in Appendix 2.

2.2 Chemotherapy vs. BSC comparison (exploratory)

2.2.1 Primary endpoint
The primary endpoint for the exploratory chemotherapy vs. BSC comparison is overall survival. Analysis of this endpoint concerns the superiority of chemotherapy over best supportive care and compares BSC with Level C OxCap. The null hypothesis to be investigated is that there is no difference in terms of overall survival between the BSC and Level C OxCap. The alternative hypothesis is that there is a difference, with superiority of Level C OxCap anticipated.

2.2.2 Secondary endpoints
The following endpoints are assessed for superiority of Level C OxCap against BSC, with respect to each endpoint. In each case, the null hypothesis is that there is no difference between the BSC and Level C OxCap, in terms of the relative endpoint. The alternative hypotheses are that there is a difference, with superiority of Level C OxCap anticipated.

- Participant reported fatigue
- QoL

2.2.3 Derivation of primary and secondary endpoints
The derivation of overall survival, participant reported fatigue and QoL is as given above.

2.3 Missing data
Attempts will be made to retrieve missing data via a thorough data cleaning process. Every effort will be made to obtain complete dates for all key data, and missing dates will be monitored.

Completely missing dates are expected to be very rare. If, however, a patient is known to have died / progressed but no date of death / progression is available, their survival / progression-free survival will be censored at the last date they were known to be alive / alive and progression-free. If there are >5% of patients with completely missing dates for progression, sensitivity analyses will be performed in which the patient is classed as having an event at the date of follow-up where progressive disease was noted. The proportion of patients with missing data, although expected to be rare, will be summarised according to treatment arm.

If an exact death / progression date is unknown, however a month and year is known, the patient will be assumed to have died / progressed on the 15th of the month with the exception of the following situations: The imputed death/progression date will be cross checked with other patient dates to make sure that the imputed death/progression date is not before any dates when the patient is known to be still alive/ not progressed, in which case the date midway between the date where they were known to be still alive/ not progressed and the end of the month will be used. If it is known that the death/ progression date is before a particular date, the date used will be half way between the earliest and latest possible dates when death/ progression could have occurred i.e. if it is known that the participant died/progressed before a certain date e.g. 14th we would use the

date half way between 1st and 14th. Sensitivity analyses will be conducted using the earliest and latest possible day of the month if there are >5% of partially missing dates.

For the analysis of participant reported fatigue, QoL and symptoms that are components of either the QLQ-C30, QLQ-OG25 or EQ5D, the appropriate scoring manual will be followed and missing items within individual outcome measures will be treated according to the instructions for that particular measure provided ≤50% of item data are missing. If the level of missing item data is >50% then the outcome will be set as missing. An exception to this is deterioration of fatigue, which is discussed further in the paragraph below. Furthermore, if there is a significant amount of missing data, and missing data patterns suggest data are missing not at random, the missing data will be accounted for in the analyses as detailed in Section 5.2. For the chemotherapy side effects questions, the number of patients with a missing response to each question will be summarised under a category of "missing". If the response to a leading yes/no question is missing but the corresponding "If yes,…" question has been answered (i.e. the data indicates the leading question should have been answered "yes"), this will be included as a "yes" rather than "missing".

If a participant has a missing questionnaire(s) and has not experienced a deterioration of participant reported fatigue, they will be censored at their last questionnaire completion date. If a participant dies following a missing questionnaire their deterioration of fatigue will also be censored at their last questionnaire completion date.

If a participant is known to have had a deterioration of participant reported fatigue but the preceding questionnaire (or fatigue component of the preceding questionnaire) was not completed, for the main analysis their time to deterioration of fatigue will be censored at the last date they were known to be without a deterioration of participant reported fatigue. For example, if a patient completes a questionnaire at the end of cycle 4, with no deterioration, does not complete the questionnaire at the end of cycle 5, and then goes on to complete a questionnaire at the end of cycle 6, with a deterioration in fatigue, this would be censored at the cycle 4 time-point.

If there are >5% of participants with a deterioration with a missing questionnaire directly preceding this, two sensitivity analyses will be performed. The first sensitivity analysis will be such that the participant is classed as having the event (deterioration of fatigue) at the time of the missing questionnaire. The second sensitivity analysis will class the participant as having the event at the time of the completed questionnaire showing a deterioration. If there are >5% of missing data of this type, multiple imputation techniques may also be applied.

If a participant does not complete the LHA or the EORTC QLQ-C30 Global QoL subscale within the LHA is derived as missing (according to the EORTC scoring manual), the following assumptions will be made: if the participant is known to have died within 3 months post-randomisation, they will be classed as having a major deterioration in Global QoL for the OTU endpoint; if the participant is not known to have died within 3 months, they will be classed as having no major deterioration in Global QoL. A similar approach will be used for any missing data for the questions '*How much has your treatment interfered with your normal daily activities?*' and '*How worthwhile do you think your treatment has been?*': if the participant is known to have died within 3 months post-randomisation, their responses will be classed as '*Very much/quite a bit*' and '*Not at all*' respectively for the OTU endpoint; if the participant is not known to have died within 3 months, their responses will not be classed as '*Very much/quite a bit*' and '*Not at all*' respectively. If there are >5% of participants with missing data for a specific participant-reported component of the OTU endpoint, two sensitivity analyses will be performed. The first sensitivity analysis will assume participants have no major deterioration in Global QoL / their responses are not '*Very much/quite a bit*' / '*Not at all*'. The second sensitivity analysis will assume the alternative i.e. that participants have a major deterioration in Global QoL / their responses are '*Very much/quite a bit*' / '*Not at all*'. If there are >5% of missing data of this type, multiple imputation techniques may also be applied.

Toxicity data, i.e. ARs and SAEs, are monitored throughout the trial and a consolidation of AEs and SAEs is undertaken to ensure the relevant ARs are reported as SAEs where appropriate, and vice-versa. This is done on an ongoing basis by data management using database reports and validations. If, at the time of analysis, a toxicity section of a CRF contains some missing and some non-missing data, the events with missing data will be assumed to be not experienced, unless it is otherwise obvious that the event was experienced (e.g. a CTCAE grade is given or 'Yes' is ticked to indicate the event met the criteria of an SAE).

For the best response endpoint, patients who are included in the RECIST evaluable population but with no response assessments at the relevant follow-up time point (i.e. 9, 18, 27, 36 and 52 weeks) will be classed in an additional category, "missing". This is for the time point at which there is no response assessment only, and response assessments from other time points will be used to determine best response. A participant will only

have a best response of “missing” if their response assessments are missing at each follow-up time point. When calculating response rates, patients in the missing category will be included in the denominator. The proportion of patients with missing or not assessable response data will be summarised according to treatment arm.

3. Populations

Patients considered eligible for the study are those that fulfil all the inclusion and none of the exclusion criteria noted in the version of the protocol under which that patient is randomised. All patients should remain in the trial after randomisation unless they actively withdraw consent.

Analysis of the primary endpoint (chemotherapy intensity comparison) will be performed on both the intention-to-treat (ITT) population and the per-protocol (PP) population. If a difference is seen between these analyses, the remaining endpoints (excluding best response and toxicity as detailed below) will also be performed on both populations. If no difference is seen between the ITT and PP populations, the remaining endpoints will be performed on the ITT population only. The toxicity endpoint will be analysed using the safety population and the best response endpoint will be analysed using the RECIST evaluable population.

For the superiority endpoints, the ITT analysis will be given primacy; a per-protocol analysis will only be performed where a significant number of participants are not in the PP population (>5%). However for the non-inferiority endpoints, equal weighting will be given to both the ITT and per-protocol analyses, as the ITT is likely to be the least conservative approach when testing for non-inferiority.

3.1 Intention-to-treat population (ITT)

The intention-to-treat population will consist of all patients randomised into the trial regardless of whether they were eligible and/or remained in the trial. In the ITT population, patients will be grouped according to the treatment they were randomised to receive.

3.2 Per-protocol population (PP)

As decided based on discussions with the co-chief investigators, the per-protocol population will consist of participants who are not classed as major protocol violators. Major protocol violators include:

- Participants who deviate from ALL of the following objective clinical eligibility criteria at randomisation (N.B. a deviation from one or two criteria only is not deemed clinically significant and will not be classed as a major protocol violation):
  - Renal function: GFR ≥30 ml/min (estimated or measured)
  - Hepatic function*: bilirubin <3 times upper limit of normal (xULN) (Protocol Version 5.0 and below)
  - Hepatic function*: bilirubin <2 times upper limit of normal (xULN) and AST or ALT <5 times upper limit of normal (xULN) (Protocol Version 6.0 and above)
  - Bone marrow function: absolute neutrophil count ≥1.5 x10^9/l; white blood cell count ≥3 x10^9/l; platelets ≥100 x10^9/l
- Participants randomised to chemotherapy who do not receive any trial treatment
- Participants who were subsequently found not to have histologically or cytologically confirmed carcinoma of the oesophagus, GO-junction or stomach.

* From Protocol Version 6.0 the eligibility criterion for hepatic function was updated. Participants’ eligibility will be assessed according to the version of the protocol they were randomised to.

Renal function and bone marrow function will be checked against the baseline data collected on the CRFs. Hepatic function cannot be checked as the upper limit of normal for bilirubin is not collected at baseline for each participant.

Participants who deviate sufficiently from the protocol, e.g. participants who do not comply with their allocated dose of treatment, will be determined on a case-by-case basis, on an assessment of the participants’ data by the DMEC. Dose reductions and dose escalations will also be monitored by the DMEC and a plan will be developed to outline how to deal with patients who have dose reductions or dose escalations in the per-protocol analysis population. This decision will be made through open discussion with the DMEC, and may include sensitivity analyses if deemed necessary.

Participants will be summarised according to the treatment received in the first treatment cycle (see Section 3.3).

3.3 Safety population
The safety population will include all participants who receive at least one dose of any trial treatment. Participants whose starting dose is within 10% of their allocated dose (as randomised) will be included in the treatment arm which they were randomised to, as shown below.

Table 3. Boundaries of each randomised dose level +/-10% for safety population

Randomised treatment arm / allocated dose level	Allocated dose -10% (in terms of the relevant 'full Level A dose'*)	Allocated dose +10% (in terms of the relevant 'full Level A dose'*)
Level C OxCap	54%	66%
Level B OxCap	72%	88%
Level A OxCap	90%	110%

*The 'full Level A dose' will be lower for participants requiring a 25% dose reduction for impaired renal or hepatic function

Following the protocol and by using dose banding for Capecitabine, there should not be any participants whose starting dose does not fall within +/-10% of their randomised dose. However, for any instances where a participant's starting dose does not fall within +/-10% of one of the treatment arms (e.g. if the dose lies between 66% and 72%, or between 88% and 90%), the absolute cut-points 70% and 90% will be used to determine which arm the participant will be included in i.e. participants receiving ≤69.9% of the relevant full dose will be included in the Level C arm, participants receiving 70-89.9% of the relevant full dose will be included in the Level B arm, whilst participants receiving ≥90% of the relevant full dose will be included in the Level A arm.

Analyses based on the safety population will first summarise participants according to their starting dose (i.e. treatment received in the first treatment cycle), but may also be summarised taking into account dose reductions, as deemed appropriate.

3.4 RECIST evaluable population
The RECIST evaluable population will include all participants who had disease which was evaluable by RECIST criteria at baseline. If there are discrepancies between the baseline and follow-up CRFs as to whether or not the participant was RECIST evaluable at baseline, the information provided at follow-up will be used e.g. if the 9-week CRF states that the participant was not RECIST evaluable at baseline, this information will be assumed to be correct.

4. Data Handling

4.1 Data monitoring
Data will be monitored for quality and completeness by the CTRU. Missing data will be chased until it is received, confirmed as not available or the trial is at analysis. However missing data items will not be chased from participants, i.e. those relating to patient reported outcomes (although research nurses will perform a check of questionnaires completed in clinic).

The CTRU/Sponsor will reserve the right to intermittently conduct source data verification exercises on a sample of participants, which will be carried out by staff from the CTRU/Sponsor. Source data verification will involve direct access to patient notes at the participating hospital sites and the ongoing central collection of copies of consent forms and other relevant investigation reports.

An independent data monitoring and ethics committee (DMEC) will review the safety and ethics of the trial as described in Section 1.5.

The following will also be examined continuously during the course of the trial:

- Accrual
- Data quality
- CRF compliance
- Compliance with the protocol
- Pregnancy
- Withdrawal from the trial

4.2 Data validation

The Data Manager will carry out initial validation of the forms in accordance with the guidelines developed for the study. This will ensure that data is complete, consistent and up-to-date. Reasons should be obtained when data is unobtainable.

The database will also validate most dates and data in line with the pre-programmed validation rules in real time, as data is entered. Periodic batch validation will also be carried out to detect any data queries that may be missed if CRFs are entered in an order that does not allow the real time validation checks to work.

Key data items required are those for the primary endpoint (see Section 2.1.1), determination of safety and per-protocol population grouping and for treatment and withdrawal information. The following key data items will be checked manually by the data managers (or their delegate).

- Date of death
- Date of progression
- Date last known to be alive or progression-free, for participants who have not progressed or died
- Dose prescribed in cycle 1
- Withdrawal of consent and date

The following key data items do not require manual checking as the data that is auto-inserted into MACRO from the 24-hour randomisation system will be used.

- Date of randomisation
- Treatment allocation
- Minimisation factors

SAS will also be used to further validate the data and identify any missing or inconsistent data. Checks to be performed include:

- Eligibility checks (if not database validations)
- Sequential dates (if not database validations)
- Checks for unusual and outlying data (if not database validations)
- Checks for missing data (are there items of data which are systematically missing/do specific variables have a large amount of missing data etc.) (if not database validations)
- Other checks, as deemed appropriate

Any inconsistent data will be noted and an e-mail sent to the data manager responsible for the study (or their delegate). All queries will be resolved, if possible in time for final analysis and the outcome documented.

5. Data Analysis

5.1 General calculations

Unless otherwise stated, percentages will be calculated using the total number of patients in the appropriate population as the denominator (i.e. including all patients with missing data for that variable). All percentages, means, medians and interquartile ranges will be rounded to one decimal place (or 1 significant figure for values less than 1) and standard deviations to two decimal places. P-values will be rounded to four decimal places (those less than 0.0001 will be displayed as <0.0001) and parameter estimates, standard errors (SEs), odds ratios, hazard ratios (HR) and confidence intervals (CIs) will be reported to one decimal place (or 1 significant figure for values less than 1). Values that are below the limit of detection and therefore non-quantifiable will be summarised using the limit of quantification value. For listings, if required, the non-quantifiable value would be reported as an inequality. All analyses will be carried out using SAS unless stated otherwise.

5.2 Analysis

The comparisons to be made in the statistical analyses are given in Section 1.3. For the chemotherapy intensity comparison, we will compare both Level B OxCap and Level C OxCap with Level A OxCap i.e. two separate comparisons. For the chemotherapy vs. BSC comparison, we will compare BSC with Level C OxCap, from the uncertain benefit pathway. Analysis of this comparison will be exploratory in nature.

5.2.1 Study summary

The CONSORT flow diagram will be used to summarise the course of patients through the study. Protocol violations will be summarised, including violations of eligibility criteria on entry into the study and subsequent deviations from the protocol.

The number of patients randomised to the study who do not go on to receive any study treatment will be summarised. The number of cycles received by those patients randomised to a chemotherapy arm will also be summarised, along with reasons for stopping treatment. The number of dose reductions and delays will be summarised for both drugs separately and overall, by treatment group.

Relative dose intensity (RDI) at 9 and 18 weeks post-treatment start (i.e. calculated from the date treatment started to 9 and 18 weeks later) will be summarised for each treatment group, both for each drug separately and overall (average RDI). This will be calculated relative to the participant's full dose in the relevant allocated treatment group (Level A, Level B or Level C OxCap, taking into account BSA (body surface area) and dose reductions due to renal impairment, and banded for Capecitabine) to two decimal places and presented using descriptive summary statistics. Patients who have died or stopped treatment due to progression within 9/18 weeks post-treatment start will be censored at their date of death/progression.

The number of participants who, at cycle 1, are prescribed a starting dose which is lower or higher than their calculated/allocated dose (according to their randomised dose level, BSA and dose reductions due to renal impairment) will be summarised. A sensitivity analysis for RDI, using the participants starting dose as the reference, rather than their calculated/allocated dose, will be considered if >5% of participants have a starting dose different to their calculated/allocated dose.

The median follow-up time will be summarised overall and for patients still alive at the time of analysis, by treatment arm and overall.

The number of withdrawals of consent to the study will be summarised, along with reasons for withdrawal. A listing of all withdrawals from the study, broken down by centre, giving a patient identifier, the reason for withdrawal, the treatment, and the duration of treatment before withdrawal will be presented.

5.2.2 Baseline characteristics

Baseline characteristics, as reported on baseline assessments and randomisation forms, will be tabulated using summary statistics overall and by arm. No statistical testing will be carried out on these data. Randomisation data from both F03 and F00 will be summarised. Summaries of the number of incorrect data on the 24-hour randomisation form (F00) compared to F03 will be produced.

Chemotherapy intensity comparison

5.2.3 Primary endpoint: Progression-free survival (non-inferiority)

Progression-free survival curves will be calculated using the Kaplan-Meier method. Participants without a PFS event at the time of analysis will be censored at the time they were last known to be alive and progression-free. Median progression-free survival estimates and progression-free survival estimates at 4, 6 and 12 months, and other fixed time-points as necessary, with corresponding 90% confidence intervals will be presented by treatment group. A log-rank test, stratifying for the minimisation factors (excluding centre), will be used to compare progression-free survival between the treatment groups. The 90% CI of the difference in PFS at 4 months, and other fixed time-points as necessary, will also be presented to aid interpretation. The number of PFS events, broken down by progression or death, will be presented by treatment group.

Cox's Proportional Hazards model, if appropriate, adjusting for the minimisation factors (excluding centre), will also be used to compare PFS between the treatment groups. Treatment HRs and corresponding 90% CIs will be obtained, and the upper limit of the CI for PFS compared with the non-inferiority margin, after the CIs have been adjusted for multiplicity[13]. Treatment and covariate estimates, standard errors, hazard ratios and 90% confidence intervals will be presented for all variables incorporated in the model.

The proportional hazards assumption will be assessed by plotting the hazards over time (i.e. the log cumulative hazard plot) for each treatment group. The 'ASSESS' statement in SAS's PHREG procedure, if appropriate, will also be used to check the proportional hazards assumption; this statement uses the methods of Lin et al[17] to check the adequacy of the Cox regression model. If the proportional hazards assumption is violated, alternative methods such as piecewise Cox models or parametric modelling will be investigated as deemed appropriate.

5.2.4 Secondary endpoints

All data received from QoL questionnaires will be included in the final analysis. In addition a plot will be presented to show how closely the data adheres to the QoL time points. QoL questionnaires within +/-4 weeks of the specified time points will be included in the compliance level for the analyses.

Participant reported fatigue (superiority)
Participant reported fatigue will be summarised for each treatment arm at each post-randomisation time-point, using adjusted for baseline mean scores and 95% CIs. These summaries and differences between treatment arms will be obtained and compared using a multi-level repeated measures random coefficients model accounting for data at all post-baseline time points, regardless of time of completion for the time-point not of interest, assuming missing data at random [MAR] and allowing for time, treatment, treatment-time interaction, and adjusting for baseline QoL and the minimisation factors (excluding centre) [all fixed effects] and for participant and participant-time interaction [random coefficients].

Data will also be summarised descriptively using bar charts, box plots, plots of mean QoL over time and summary tables. Missing data patterns will be examined carefully and sensitivity analyses using different missing data assumptions will be performed if appropriate. Analyses may be carried out using methods such as: multiple imputation; pattern-mixture multi-level models categorising participants into strata based on clinical information which is believed to represent the reasons for missing data (assuming MAR data conditional upon participants' clinical data); and pattern mixture models for bivariate (baseline and 9 week) data fitted using a variety of restrictions reflecting the missing data pattern ranging from complete case missing variable restriction (MAR) to Brown's protective restriction (assuming data are missing not at random (MNAR)).[18]

Time to deterioration of participant reported fatigue (superiority)
Time to deterioration of participant reported fatigue will be investigated using cumulative incidence function curves and the median time to deterioration and 95% confidence intervals will be presented by treatment group. Participants without deterioration of fatigue and who are not known to have died within 1 year of randomisation will be censored at their last questionnaire completion date. Participants who have died within 1 year of randomisation without evidence of deterioration of fatigue will be censored at their date of death in the analysis estimating the treatment effect via Cox's Proportional Hazards model and classed as having a competing-risk event (i.e. not censored) in the analysis estimating, and comparing, the incidence of deterioration of fatigue (i.e. the cumulative incidence function curves and Gray's test[19]), as well as in the analysis of the treatment effect in the presence of competing risks via a Fine and Gray model.[20]

Gray's test[19], stratifying for the minimisation factors (excluding centre), will be used to compare the cumulative incidence functions for time to deterioration of fatigue between the treatment groups. Cox's Proportional Hazards model, if appropriate, adjusting for the minimisation factors (excluding centre), will also be used to compare time to deterioration of fatigue between the treatment groups. Treatment and covariate estimates, standard errors, hazard ratios, 95% confidence intervals and p-values will be presented for all variables incorporated in the model. A Fine and Gray model[20], if appropriate, may also be used to compare time to deterioration of fatigue between the treatment groups, in the presence of competing risks. This will adjust for the minimisation factors (excluding centre). Treatment and covariate estimates, standard errors, hazard ratios and p-values will be presented for all variables incorporated in the model.

The proportional hazards assumption will be assessed by plotting the hazards over time (i.e. the log cumulative hazard plot) for each treatment group. The 'ASSESS' statement in SAS's PHREG procedure, if appropriate, will also be used to check the proportional hazards assumption; this statement uses the methods of Lin et al[17] to check the adequacy of the Cox regression model. If the proportional hazards assumption is violated, alternative methods will be investigated as deemed appropriate. Missing data will be accounted for, as described in Section 2.3.

Overall treatment utility (superiority)
Overall treatment utility (OTU) will be calculated as per Appendix 1 at 9 weeks post randomisation and summarised by calculating the differences in rates between the treatment groups with corresponding 95% CIs. Treatment groups will be compared using ordered logistic regression to adjust for the minimisation factors (excluding centre) and, in a secondary analysis, other covariates identified as being potentially prognostic of outcome, as given in Appendix 3. Treatment and covariate estimates, standard errors, odds ratios, 95% confidence intervals and p-values will be presented for all variables incorporated in the model.

Sensitivity analyses using revised definitions of OTU, but still incorporating the same information and questions, may be performed as appropriate. The research questions will not change, rather the boundaries regarding the level of deterioration required or the responses to patient reported outcomes included as not tolerable may be investigated.

PFS and OS by OTU status at 9 weeks (superiority)

Progression-free survival and overall survival curves will be calculated using the Kaplan-Meier method and PFS/OS estimates as appropriate will be presented by OTU status at 9 weeks post-randomisation. A log-rank test will be used to compare progression-free survival and overall survival between the OTU status groups, adjusting for treatment group.

The Cox proportional hazards model (if appropriate), adjusting for relevant factors as appropriate, will also be used to compare progression-free survival and overall survival between the OTU status groups. Factors that could be adjusted for as appropriate include treatment received, minimisation factors (excluding centre) and other important prognostic factors identified, and a treatment by OTU status interaction. OTU status and other covariate estimates, standard errors, hazard ratios, 95% confidence intervals, as well as p-values will be presented for each model investigated.

This analysis will compare OTU status groups.

QoL & symptoms (superiority)
Quality of life, including global QoL and symptoms, excluding the chemotherapy side effects questions, will be summarised for each treatment arm at each post-randomisation time-point, using adjusted for baseline mean scores and 95% CIs. These summaries and differences between treatment arms will be obtained and compared using a multi-level repeated measures random coefficients model accounting for data at all post-baseline time points, regardless of time of completion for the time-point not of interest, assuming missing data at random [MAR] and allowing for time, treatment, treatment-time interaction, and adjusting for baseline QoL and the minimisation factors (excluding centre) [all fixed effects] and for participant and participant-time interaction [random coefficients].

Data will also be summarised descriptively using bar charts, box plots, plots of mean QoL over time and summary tables. Missing data patterns will be examined carefully and sensitivity analyses using different missing data assumptions will be performed if appropriate.

Sensitivity analyses may be carried out using methods such as: multiple imputation; pattern-mixture multi-level models categorising participants into strata based on clinical information which is believed to represent the reasons for missing data (assuming MAR data conditional upon participants' clinical data); and pattern mixture models for bivariate (baseline and 9 week) data fitted using a variety of restrictions reflecting the missing data pattern ranging from complete case missing variable restriction (MAR) to Brown's protective restriction (assuming data are missing not at random (MNAR)).

The chemotherapy side effects questions will be summarised descriptively using summary tables. There is no scoring manual for this section of the 9 week Limited Health Assessment (LHA) so the analysis of these questions will not include any modelling.

Quality adjusted survival (superiority)
Quality adjusted survival (QAS) will be calculated up to 1 year follow-up for each participant using the methods described by Billingham and Abrams[21]. This will rely on the longitudinally measured EQ-VAS to weight QoL based on participant preferences. Initial analysis will rely on the integrated quality-survival product. The analysis will be repeated for QoL weight measured by the EQ-5D tariff. The role of missing data will be tested by sensitivity analysis and, where appropriate, by imputation. If missing data are thought to be causing bias in QAS or if there is a need to extrapolate survival and QoL outcomes beyond the available data then a second approach will use a multistate transition model with dropout-specific and health-specific states. We will compare both Level B OxCap and Level C OxCap with Level A OxCap i.e. two separate comparisons. This analysis will be performed by Dr Peter Hall.

Overall survival (non-inferiority)
Overall survival (OS) curves will be calculated using the Kaplan-Meier method and the median overall survival estimates, overall survival estimates at 6 and 12 months, and other fixed time-points as necessary, and 90% confidence intervals will be presented by treatment group. The number of deaths will be presented by treatment group. Participants without an OS event at the time of analysis will be censored at the time they were last known to be alive.

A log-rank test, stratifying for the minimisation factors (excluding centre), will be used to compare overall survival between the treatment groups. Cox's Proportional Hazards model, if appropriate, adjusting for the minimisation factors (excluding centre) will also be used to compare OS between the treatment groups. Treatment and covariate estimates, standard errors, hazard ratios and 90% confidence intervals will be

presented for all variables incorporated in the model.

The proportional hazards assumption will be assessed by plotting the hazards over time (i.e. the log cumulative hazard plot) for each treatment group. The 'ASSESS' statement in SAS's PHREG procedure, if appropriate, will also be used to check the proportional hazards assumption; this statement uses the methods of Lin et al[17] to check the adequacy of the Cox regression model. If the proportional hazards assumption is violated, alternative methods will be investigated as deemed appropriate.

A non-inferiority margin has not been pre-specified for this analysis. The level of non-inferiority that can be attained will be determined by the upper limit of the 90% confidence interval (hazard ratio), as detailed in Section 2.1.3. The results of this analysis will be interpreted through discussion with the TMG and patient representatives at the time of final analysis.

Best response (non-inferiority)
Best response within 1 year of randomisation will be summarised by the proportion of participants achieving each best response (complete response, partial response, stable disease, progressive disease or missing response (if necessary)).[22] The differences in rates between the treatment groups will be presented with corresponding 90% CIs and compared using ordered logistic regression to adjust for the minimisation factors (excluding centre). Treatment and covariate estimates, standard errors, odds ratios, 90% confidence intervals and p-values will be presented for all variables incorporated in the model.

A non-inferiority margin has not been pre-specified for this analysis. The level of non-inferiority that can be attained will be determined by the lower limit of the 90% confidence interval for the odds ratio, as detailed in Section 2.1.3. The results of this analysis will be interpreted through discussion with the TMG and patient representatives at the time of final analysis.

Toxicity
To assess toxicity, the maximum grade per participant for each toxicity overall and per cycle will be summarised descriptively for each treatment group. Percentages will be calculated using the number of patients with at least some non-missing data as the denominator. Treatment delays and modifications, as described in Section 5.2.1, and withdrawals will also be summarised together with additional safety data e.g. SAEs, SARs, SUSARs and deaths within 30 days of last treatment administration or which are considered to be related to treatment. Analyses based on the safety population will first summarise participants according to their starting dose (i.e. treatment received in the first treatment cycle), but may also be summarised taking into account dose reductions, as deemed appropriate. For example, if a participant is receiving Level A and then has their dose reduced to 80% due to toxicity or SAEs, the first analysis would include the participant in the Level A arm, but may later look at separating the toxicity or SAEs experienced whilst receiving 80% (equivalent to Level B) from those experienced whilst receiving 100% Level A.

Frailty analyses
The impact of baseline frailty and its prospectively defined constituents (considered individually) on outcomes and treatment effect will be assessed for the progression-free survival, overall survival, overall treatment utility, QoL & symptoms and toxicity endpoints, using the methods summarised above for each endpoint.

The primary analysis of frailty will use impairment in two or more domains (as given in Appendix 2) as the cut-off for frailty to define participants as frail or not frail. Both the prognostic and predictive effect of baseline frailty will be assessed, incorporating a frailty-treatment interaction term in multivariate models where appropriate, and performing a subgroup analysis by frailty where this is not possible (e.g. for the toxicity endpoint).

Analyses of comprehensive geriatric assessment (CGA) scores (i.e. the domains used in the definition of frailty, as given in Appendix 2) will also be performed to determine whether increasing score is associated with worse outcomes and to assess heterogeneity of the treatment effect on outcomes.

Sensitivity analyses may be performed as appropriate using different cut-off points and scoring criteria for the definition of frailty.

Further exploratory analyses

Subgroup analyses
Subgroup analyses for the clinical randomisation factors and other baseline participant characteristics will be performed to investigate whether there is heterogeneity of treatment effect on outcomes. Specific covariates

of interest, additional to the clinical randomisation factors, will include those listed in Appendix 3.

Exploratory prognostic factor analyses
Baseline participant characteristics and items in the CHA will be investigated to determine whether they are prognostic of outcomes. Specific covariates of interest, additional to the clinical randomisation factors, will include those listed in Appendix 3.

Tolerability of treatment
The impact of baseline frailty on 9-week tolerability will use impairment in two or more domains (as given in Appendix 2) as the cut-off to define participants as frail or not frail. A participant will be classed as not tolerating treatment if they have had one or more of the following events: death due to toxicity; SAR (serious adverse reaction) requiring or prolonging hospitalisation (includes SUSARs); stopped treatment due to toxicity; and dose reduced but continued past cycle 3.

Tolerability will be presented as a hierarchy of categorical reasons for not tolerating treatment and ordered logistic regression will be used based on the following scoring: 0-Tolerates treatment (reference category); 1-Tolerates to some degree: dose is reduced but treatment continues past cycle 3; 2-Treatment is not tolerated: death due to toxicity; SAR requiring or prolonging hospitalisation (includes SUSARs); stopped treatment due to toxicity. Where appropriate interaction terms between treatment and frailty will be incorporated.

Sensitivity analyses may be performed as appropriate using different cut-off points and scoring criteria for the definition of frailty. Further analyses may also be performed to investigate the domains included in the comprehensive geriatric assessment (CGA) used to define frailty (Appendix 2) to determine those that are potentially significant predictors of tolerability.

Chemotherapy vs. BSC comparison (exploratory)

5.2.5 Primary endpoint: overall survival (superiority)
Overall survival (OS) curves will be calculated using the Kaplan-Meier method and the median overall survival estimates, overall survival estimates at 6 and 12 months, and other fixed time-points as necessary, and 95% confidence intervals will be presented by treatment group. The number of deaths will also be presented by treatment group. Analysis of this endpoint concerns the superiority of Level C OxCap over best supportive care in terms of overall survival. A log-rank test, stratifying for the minimisation factors (excluding centre), will be used to compare overall survival between the treatment groups. Participants without an OS event at the time of analysis will be censored at the time they were last known to be alive.

Cox's Proportional Hazards model, if appropriate, adjusting for the minimisation factors (excluding centre), will also be used to compare OS between the treatment groups. Treatment and covariate estimates, standard errors, hazard ratios, 95% confidence intervals and p-values will be presented for all variables incorporated in the model.

The proportional hazards assumption will be assessed by plotting the hazards over time (i.e. the log cumulative hazard plot) for each treatment group. The 'ASSESS' statement in SAS's PHREG procedure, if appropriate, will also be used to check the proportional hazards assumption; this statement uses the methods of Lin et al[17] to check the adequacy of the Cox regression model. If the proportional hazards assumption is violated, alternative methods will be investigated as deemed appropriate.

5.2.6 Secondary endpoints: participant reported fatigue and QoL (superiority)
Quality of life, including fatigue, will be summarised for each treatment arm at each post-randomisation time-point, using adjusted for baseline mean scores and 95% CIs. These summaries and differences between treatment arms will be obtained and compared using a multi-level repeated measures random coefficients model accounting for data at all post-baseline time points, regardless of time of completion for the time-point not of interest, assuming missing data at random [MAR] and allowing for time, treatment, treatment-time interaction, and adjusting for baseline QoL and the minimisation factors (excluding centre) [all fixed effects] and for participant and participant-time interaction [random coefficients] where appropriate.

Data will also be summarised descriptively using bar charts, box plots, plots of mean QoL over time and summary tables. Missing data patterns will be examined carefully and alternative analyses using different missing data assumptions will be performed if appropriate.

6. Reporting and Dissemination of the Results

Final analysis will take place once the minimum required number of PFS events have occurred (as specified in the sample size calculation): 284 in each comparison in the certain benefit pathway (Level B vs. Level A and Level C vs. Level A); or when the most recently randomised, surviving participant has been followed up for 1 year post randomisation, whichever is reached sooner. A further updated analysis of overall survival will be performed following collection of extended survival data from sites approximately 1 year post randomisation of the final participant.

After each analysis is complete, the results will be presented to the project team who will discuss them and decide if any further analysis or investigation is required. After this, members of the project team will write up the results with a view to submitting a manuscript to a peer-reviewed scientific journal. The results will also be submitted as abstracts to appropriate conferences for either poster or oral presentation. All abstracts and manuscripts must be reviewed by each member of the project team and an external referee if appropriate, before submission.

To maintain the scientific integrity of the trial, data will not be released prior to the first publication of the analysis of the primary endpoint, either for trial publication or oral presentation purposes, without the permission of the Trial Steering Committee. In addition, individual collaborators must not publish data concerning their participants which is directly relevant to the questions posed in the trial until the first publication of the analysis of the primary endpoint.

7. References

1. Cancer_Research_UK. CancerStats. http://info.cancerresearchuk.org/cancerstats 2007.

2. Kocher HM, Linklater K, Patel S, Ellul JP. Epidemiological study of oesophageal and gastric cancer in south-east England. *Br J Surg* 2001;88(9):1249-57.

3. MacMillan Cancer Support DaAU. Cancer Services Coming of Age: Learning from the Improving Cancer Treatment, Assessment and Support for Older People Project. . http://www.macmillan.org.uk/Documents/AboutUs/Health_professionals/OlderPeoplesProject/CancerServicesComingofAge.pdf 2012.

4. Cunningham D, Starling N, Rao S, Iveson T, Nicolson M, Coxon F, et al. Capecitabine and oxaliplatin for advanced esophagogastric cancer. *N Engl J Med* 2008;358(1):36-46.

5. Popescu RA, Norman A, Ross PJ, Parikh B, Cunningham D. Adjuvant or palliative chemotherapy for colorectal cancer in patients 70 years or older. *J Clin Oncol* 1999;17(8):2412-8.

6. Stein BN, Petrelli NJ, Douglass HO, Driscoll DL, Arcangeli G, Meropol NJ. Age and sex are independent predictors of 5-fluorouracil toxicity. Analysis of a large scale phase III trial. *Cancer* 1995;75(1):11-7.

7. Ross P, Nicolson M, Cunningham D, Valle J, Seymour M, Harper P, et al. Prospective randomized trial comparing mitomycin, cisplatin, and protracted venous-infusion fluorouracil (PVI 5-FU) With epirubicin, cisplatin, and PVI 5-FU in advanced esophagogastric cancer. *J Clin Oncol* 2002;20(8):1996-2004.

8. Seymour MT, Thompson LC, Wasan HS, Middleton G, Brewster AE, Shepherd SF, et al. Chemotherapy options in elderly and frail patients with metastatic colorectal cancer (MRC FOCUS2): an open-label, randomised factorial trial. *Lancet* 2011;377(9779):1749-59.

9. Seymour MT, Maughan TS, Ledermann JA, Topham C, James R, Gwyther SJ, et al. Different strategies of sequential and combination chemotherapy for patients with poor prognosis advanced colorectal cancer (MRC FOCUS): a randomised controlled trial.[Erratum appears in Lancet. 2007 Aug 18;370(9587):566]. *Lancet* 2007;370(9582):143-52.

10. Cohen J. *Statistical power analysis for the behavioural sciences*. 2nd ed. Hillsdale, N.J: L. Erlbaum Associates, 1988.

11. Lancaster GA, Dodd S, Williamson PR. Design and analysis of pilot studies: recommendations for good practice. *J Eval Clin Pract* 2004;10(2):307-12.

12. Wagner AD, Grothe W, Haerting J, Kleber G, Grothey A, Fleig WE. Chemotherapy in Advanced Gastric Cancer: A Systematic Review and Meta-Analysis Based on Aggregate Data. *J Clin Oncol* 2006;24(18):2903-09.

13. Efird JT and Nielsen SS. A method to compute multiplicity corrected confidence intervals for odds ratios and other relative effect estimates. *International journal of environmental research and public health* 2008; 5(5):394-398

14. Fayers PM, Aaronson NK, Biordal K, Groenvold M, Curran D, Bottomley A, on behalf of the EORTC Quality of Live Group. EORTC QLQ-C30 Scoring Manual (3rd edition). Brussels: EORTC 2001. ISBN 2-9300 64-22-6

15. Cocks K, King MT, Velikova G, de Castro G, Jr., Martyn St-James M, Fayers PM, et al. Evidence-based guidelines for interpreting change scores for the European Organisation for the Research and Treatment of Cancer Quality of Life Questionnaire Core 30. *European Journal of Cancer* 2012;48(11):1713-21.

16. Rabin R, Oemar M, Oppe M EuroQol Group Executive Office on behalf of the EuroQol Group. EQ-5D-3L User Guide. Basic information on how to use the EQ-5D-3L instrument Version 4.0. 2011. www.euroqol.org.

17. Lin D, Wei L, Ying Z. Checking the Cox Model with Cumulative Sums of Martingale-Based Residuals. Biometrika 1993;80:557-72.

18. Fairclough DL: Design and Analysis of Quality of Life Studies in Clinical Trials. New York, Chapman & Hall, 2002, pp 153-167

19. Gray RJ. A class of *k*-sample tests for comparing the cumulative incidence of a competing risk. Annals of Statistics 1988; 16: 1141-1154.

20. Fine JP, Gray RJ. A proportional hazards model for the subdistribution of a competing risk. Journal of the American Statistical Association 1999; 94: 496-509.

21. Billingham LJ, Abrams KR. Simultaneous analysis of quality of life and survival data. Statistical Methods in Medical Research 2002; 11(1): 25-48.

22. Therasse P, Arbuck SG, Eisenhauer EA, Wanders J, Kaplan RS, Rubinstein L, et al. New guidelines to evaluate the response to treatment in solid tumors. *Journal of the National Cancer Institute* 2000;92(3):205-1

8. Appendices

Appendix 1 - Overall Treatment Utility (OTU) Definition

OTU is a novel clinical outcome measure incorporating objective and participant reported measures of anticancer efficacy, tolerability and acceptability of treatment, assessed 9 weeks post-randomisation and condensed into a simple 3-point score.

OTU may be regarded as asking the clinician: *"With the benefit of hindsight, are you glad you gave this treatment?"* and asking the participant: *"With the benefit of hindsight, are you glad you received it?"*. OTU is scored as good, intermediate or poor, corresponding to "yes", "uncertain/disagree" or "no" replies to these questions.

To score OTU, the participant is assessed 9 weeks after randomisation, using the following criteria:

1. **Is the treatment considered to have helped?**

   a. Scored as "YES" if all of the following apply:
      - No evidence of radiological progression using RECIST
      - No other clinician-assessed evidence of cancer progression[1]
      - No major deterioration in Global QoL[2]

   b. Scored as "NO" if any of the following apply:
      - Radiological progression using RECIST
      - Other clinician-assessed evidence of cancer progression
      - Major deterioration in Global QoL

2. **Is the treatment tolerable and acceptable?**

   a. Scored as "YES" if all of the following apply:
      - No SAR or SUSAR definitely attributed to treatment
      - The patient's response to the question *"How much has your treatment interfered with your normal daily activities?"* is not "Very much" or "quite a bit".
      - The patient's response to the question *"How worthwhile do you think your treatment has been?"* is not "Not at all"

   b. Scored as "NO" if any of the following apply:
      - SAR or SUSAR definitely attributed to treatment
      - The patient's response to the question *"How much has your treatment interfered with your normal daily activities?"* is "Very much" or "quite a bit"
      - The patient's response to the question *"How worthwhile do you think your treatment has been?"* is "Not at all"

Scoring:

**Good OTU:**	Patient is alive and scores are "YES" for both 1 and 2.
**Intermediate OTU:**	Patient is alive and scores are "YES/NO" or "NO/YES".
**Poor OTU:**	Scores are "NO" for both 1 and 2, or patient has died.

---

[1] Clear clinical evidence of cancer progression which has not been confirmed radiologically.
[2] A drop of 16 or more points in the EORTC QLQ-C30 Global QoL Subscale

Appendix 2 – Definition of frailty

The definition of frailty is based on 9 domains assessed at baseline, using the comprehensive health assessment (CHA).

Domains assessed at baseline (CHA)	Tools used	Proposed cut off for impaired domain
**Weight loss**	How many Kg lost in the past 3 months<br>BMI	3kg or >5% body weight or<br>BMI <18.5
**Mobility**	Timed up and go test	>10 seconds or unable to complete test
**Falls**	G8 question	Has had 2 or more falls in the past 6 months
**Cognition**	G8 question	Mild or severe dementia diagnosis
**Function**	Nottingham ADL/IADL (Activities of daily living/ Instrumental activities of daily living)	One or more impairment in IADL or ADL
**Social**	Place of residence	Requires 24 hour care
**Mood**	EQ5D question (feelings today)<br>• Anxious or depressed: not/moderately/extremely	Extremely anxious/depressed
**Fatigue**	EORTC QLQC30 questions (not at all/ a little/quite a bit/ very much<br>• During the past week did you need to rest?<br>• During the past week were you tired?	Very much for either needing to rest or was tired<br>or<br>Quite a bit for both questions
**Polypharmacy**	Number of prescribed regular medications	5 or more
**9 domains**		

A participant is deemed frail if they have **impairment in two or more domains**.

This scoring system is based on comprehensive geriatric assessment (CGA) methods used in geriatric medicine, where impairment of two or more domains is accepted as a cut-off for the identification of frailty. However, there are only a small number of published studies of frailty in older cancer patients. These have used a variety of tools to assess each domain, and some have used non-standard cut-offs for frailty. This has been summarised in a literature review performed by Cat Handforth (GO2 TMG member) and is saved separately to this analysis plan.

Appendix 3 – Covariates prognostic of outcome

This appendix will be reviewed, and updated if necessary, before any analysis begins.

The following lists other covariates (excluding the minimisation factors) identified as being potentially prognostic of outcome (with the proposed cut-points indicated where applicable) which will be considered in additional multivariate analyses:

- Age, measured continuously
- Sex
- Site of primary tumour (categorised as gastric, GO junction or oesophageal)
- Frailty and its domains, as defined in Appendix 2
- Baseline EQ-VAS or EORTC QLQ-C30 global QoL (categorised as <lower quintile or ≥lower quintile)
- Baseline EQ-5D pain (measured as a 3-level ordered categorical variable), EORTC QLQ-C30 pain (measured continuously) or EORTC QLQ-OG25 pain (measured continuously)
- Baseline EORTC QLQ-C30 nausea and vomiting (measured continuously)
- Baseline fatigue, as defined in Appendix 2
- Baseline EORTC QLQ-OG25 dysphagia (categorised as 0 or any)
- Baseline EORTC QLQ-OG25 odynophagia (categorised as 0 or any)
- Baseline EORTC QLQ-OG25 taste (categorised as 0 or any)
- Baseline cardiac strain, measured using BNP or NT-Pro-BNP (categorised as >ULN or ≤ULN)
- Baseline haemoglobin (categorised as <12 g/dl or ≥12 g/dl)
- Baseline white cell count (categorised as >11 or ≤11)
- Baseline neutrophil to lymphocyte ratio (categorised as >4.0 or ≤4.0)
- Baseline platelets (categorised as >400 or ≤400)
- Baseline GFR (formula derived) (categorised as <60ml/min or ≥60ml/min)
- Baseline bilirubin (categorised as >21 umol/l or ≤21 umol/l)
- Baseline AST or ALT (categorised as either ALT or AST >50 u/l or ≤50 u/l)
- Baseline albumin (categorised as <30 or ≥30)
- Baseline alkaline phosphatase (categorised as >2.5x institutional ULN or ≤2.5x institutional ULN)
- Baseline urea (categorised as >6 or ≤6)
- Baseline sodium (categorised as <135 or ≥135)
- Baseline CEA (categorised as >3ng/l or ≤3ng/l)
- Baseline Ca19-9 (categorised as >37 ku/l or ≤37 ku/l)

Approval of Analysis Plan

Clinical Trials Research Unit (CTRU)

The following Final analysis plan, v2.0, January 2019, for the GO2 study has been approved by the following personnel. Any signed amendments to the plan will be filed with this document.

Trial Statistician (Alina Striha): ______________________________

Date: ______________________________

Supervising statistician (Helen Marshall): ______________________________

Date: ______________________________

Senior Trial Coordinator (Sharon Ruddock):______________________________

Date: ______________________________

Delivery lead (Helen Howard): ______________________________

Date: ______________________________

Data Manager (Eszter Katona): ______________________________

Date: ______________________________

Scientific lead (Fiona Collinson): ______________________________

Date: ______________________________

Chief Investigator (Professor Matthew Seymour): ______________________________

Date: ______________________________

Chief Investigator (Dr Peter Hall): ______________________________

Date: ______________________________

Additional information:

