# Supplementary material for: Efficacy of Reduced-Intensity Chemotherapy With Oxaliplatin and Capecitabine on Quality of Life and Cancer Control Among Older and Frail Patients With Advanced Gastroesophageal Cancer: The GO2 Phase 3 Randomized Clinical Trial
Source: JAMA Oncol. 2021 May 13;7(6):869–77. doi: 10.1001/jamaoncol.2021.0848 (PMC8120440; doi:10.1001/jamaoncol.2021.0848)
Supplement: Supplement 3. — eTable 1: GO2 Study Sites and Principal Investigators eTable 2: Baseline characteristics eTable 3: Baseline Frailty domains eTable 4a: Summaries of missing data for quality of life questionnaires in CHEMO-INTENSITY pathway eTable 4b: Summaries of missing data for quality of life questionnaires in the CHEMO-BSC pathway eTable 5: EORTC-QLQ-C30 Global health status in the CHEMO-INTENSITY pathway eTable 6: EORTC-QLQ-C30 Functional scales in the CHEMO-INTENSITY pathway eTable 7: EORTC-QLQ-C30 Symptom scales in the CHEMO-INTENSITY pathway eTable 8: EORTC-QLQ-C30 Symptom scales - CHEMO-INTENSITY pathway eTable 9: EORTC-QLQ-C30 Fatigue scale in the CHEMO-INTENSITY pathway eTable 10: EORTC-OG-25 Symptom scales in the CHEMO-INTENSITY pathway eTable 11: Summaries of best response eTable 12: Factors Associated with OTU outcome eTable 13: Lookup table for multivariable prognostic model· eTable 14: Predictive ability of the multivariable prognostic model evaluated at each variable combination eTable 15: Chemotherapy delivery and tolerability (CHEMO-INTENSITY randomisation) eFigure 1: Influence of baseline factors and dose level on OTU eFigure 2: Univariate analysis: impact of baseline variables Overall Treatment Utility eFigure 3: Univariate analysis : Impact of baseline symptoms Overall Treatment Utility eFigure 4: Forest plot for CHEMO-INTENSITY PFS (Level A vs. Level B) eFigure 5: Forest plot for CHEMO-INTENSITY PFS (Level A vs. Level C) eFigure 6: Forest plot for CHEMO-INTENSITY OS (Level A vs. Level B) eFigure 7: Forest plot for CHEMO-INTENSITY OS (Level A vs. Level C) eFigure 8: EORTC-QLQ-C30 Fatigue scale in the CHEMO-INTENSITY pathway eFigure 9: EuroqoL EQ-5D-3L Score in the CHEMO-INTENSITY pathway eFigure 10: EORTC-QLQ-C30 Fatigue scale in the CHEMO-BSC pathway eFigure 11: EuroqoL EQ-5D-3L Score in the CHEMO-BSC pathway eFigure 12: Time to Deterioration of Fatigue in the CHEMO-INTENSITY Pathway eFigure 13: Per Protocol Population: PFS curves by randomised treatment arm [file jamaoncol-e210848-s003.pdf]

Supplemental Online Content

Hall PS, Swinson D, Cairns DA, et al; GO2 Trial Investigators. Efficacy of reduced-intensity chemotherapy with oxaliplatin and capecitabine on quality of life and cancer control among older and frail patients with advanced gastroesophageal cancer: the GO2 phase 3 randomized clinical trial. *JAMA Oncol*. Published online May 13, 2021. doi:10.1001/jamaoncol.2021.0848

eTable 1: GO2 Study Sites and Principal Investigators

eTable 2: Baseline characteristics

eTable 3: Baseline Frailty domains

eTable 4a: Summaries of missing data for quality of life questionnaires in CHEMO-INTENSITY pathway

eTable 4b: Summaries of missing data for quality of life questionnaires in the CHEMO-BSC pathway

eTable 5: EORTC-QLQ-C30 Global health status in the CHEMO-INTENSITY pathway

eTable 6: EORTC-QLQ-C30 Functional scales in the CHEMO-INTENSITY pathway

eTable 7: EORTC-QLQ-C30 Symptom scales in the CHEMO-INTENSITY pathway

eTable 8: EORTC-QLQ-C30 Symptom scales - CHEMO-INTENSITY pathway

eTable 9: EORTC-QLQ-C30 Fatigue scale in the CHEMO-INTENSITY pathway

eTable 10: EORTC-OG-25 Symptom scales in the CHEMO-INTENSITY pathway

eTable 11: Summaries of best response

eTable 12: Factors Associated with OTU outcome

eTable 13: Lookup table for multivariable prognostic model·

eTable 14: Predictive ability of the multivariable prognostic model evaluated at each variable combination

eTable 15: Chemotherapy delivery and tolerability (CHEMO-INTENSITY randomisation)

© 2021 Hall PS et al. *Jama Oncol.*

eFigure 1: Influence of baseline factors and dose level on OTU

eFigure 2: Univariate analysis: impact of baseline variables Overall Treatment Utility

eFigure 3: Univariate analysis : Impact of baseline symptoms Overall Treatment Utility

eFigure 4: Forest plot for CHEMO-INTENSITY PFS (Level A vs. Level B)

eFigure 5: Forest plot for CHEMO-INTENSITY PFS (Level A vs. Level C)

eFigure 6: Forest plot for CHEMO-INTENSITY OS (Level A vs. Level B)

eFigure 7: Forest plot for CHEMO-INTENSITY OS (Level A vs. Level C)

eFigure 8: EORTC-QLQ-C30 Fatigue scale in the CHEMO-INTENSITY pathway

eFigure 9: EuroqoL EQ-5D-3L Score in the CHEMO-INTENSITY pathway

eFigure 10: EORTC-QLQ-C30 Fatigue scale in the CHEMO-BSC pathway

eFigure 11: EuroqoL EQ-5D-3L Score in the CHEMO-BSC pathway

eFigure 12: Time to Deterioration of Fatigue in the CHEMO-INTENSITY Pathway

eFigure 13: Per Protocol Population: PFS curves by randomised treatment arm

This supplemental material has been provided by the authors to give readers additional information about their work.

Supplementary Tables

eTable 1 GO2 Study Sites and Principal Investigators

Centre	Total Recruited	CHEMO-INTENSITY Pathway	CHEMO-BSC Pathway	PI name
Leeds Teaching Hospitals NHS FT Trust	58	55	3	Dr Daniel Swinson
East Kent Hospitals NHS Trust	33	32	1	Dr Justin Waters
Hull and East Yorkshire Hospitals NHS Trust	25	24	1	Dr Rajarshi Roy
University Hospitals Bristol NHS Foundation Trust	25	24	1	Dr Stephen Falk
Sheffield Teaching Hospitals NHS Trust	25	25	0	Dr Jonathan Wadsley
The Royal Wolverhampton NHS Trust	23	23	0	Dr Mano Joseph
Mid Yorkshire Hospitals NHS Trust	21	18	3	Dr Kostantinos-Vellios
North Cumbria University Hospitals NHS Trust	21	21	0	Dr Jonathan Nicoll
Royal United Hospital Bath NHS Trust	20	20	0	Dr Tania Tillett
Royal Surrey County Hospital NHS Foundation Trust	19	19	0	Dr Sebastian Cummins
The Dudley Group NHS Foundation Trust	18	16	2	Dr Simon Grumett
United Lincolnshire Hospitals NHS Trust	18	10	8	Dr Zuzana Stokes
The Christie NHS Foundation Trust	16	15	1	Dr Tom Waddell
The Shrewsbury and Telford Hospital NHS Trust	14	11	3	Dr Anirban Chatterjee
Betsi Cadwaldr University LHB	13	13	0	Dr Angel Garcia
York Teaching Hospital NHS Foundation Trust (Scarb)	13	4	9	Dr Mohammed Khan
Guys and St Thomas's NHS Foundation Trust	12	11	1	Dr Nick Maisey
Worcestershire Acute Hospitals NHS Trust	12	9	3	Dr Cheng Boon
Calderdale and Huddersfield NHS Foundation Trust	11	7	4	Dr Jo Dent
Sherwood Forest NHS Foundation Trust	9	9	0	Dr Eleanor James
NHS Tayside	9	9	0	Prof Russell Petty
Bradford Teaching Hospitals NHS Foundation Trust	9	8	1	Dr Sue Cheeseman
Norfolk and Norwich University Hospitals NHS Foundation Trust	8	8	0	Dr Tom Roques
Imperial College Healthcare NHS Trust	8	8	0	Dr Nick Reed
Gloucestershire Hospitals NHS Foundation Trust	7	7	0	Dr Charles Candish
University Hospitals of Morecambe Bay NHS Foundation Trust	7	7	0	Dr David Fyfe
York Teaching Hospital NHS Foundation Trust (York)	7	6	1	Dr Kim Last
Royal Cornwall NHS Trust	7	7	0	Dr Richard Ellis
NHS Grampian	7	7	0	Dr Leslie Samuel
Western Sussex Hospitals NHS Foundation Trust	7	7	0	Dr Rebecca Herbertson
South Devon Healthcare NHS Trust	6	6	0	Dr Louise Medley
Oxford University Hospitals NHS Trust	6	6	0	Dr Kinnari Patel

Plymouth Hospitals NHS Trust	6	6	0	Dr David Sherriff
East Sussex Healthcare NHS Trust	6	6	0	Dr Angus Robinson
Blackpool Teaching Hospitals NHS Trust	5	5	0	Dr Pavel Bezecny
Betsi Cadwaldr University LHB	5	5	0	Dr Duncan Wilkins
Hinchingbrooke Health Care NHS Trust	4	4	0	Dr Adam McGeoch
Barts Health NHS Trust	4	4	0	Dr David Propper
Ysbyty Gwynedd	4	3	1	Dr Rachel Williams
Weston Area Healthcare Trust	3	3	0	Dr Serena Hilman
Barking, Havering & Redbridge University Hospitals NHS Trust	3	3	0	Dr Sherif Raouf
Great Western Hospitals NHS Foundation Trust	3	3	0	Dr Claire Blesing
The Royal Bournemouth and Christchurch Hospitals NHS Foundation Trust	3	3	0	Dr Joanne Parkinson
South Tees Hospitals NHS Foundation Trust	3	3	0	Dr Nick Wadd
Milton Keynes Hospital NHS Foundation Trust	2	2	0	Dr Wasiru Saka
Walsall Healthcare NHS Trust	2	2	0	Dr Victoria Kunene
Abertawe Bro Morgannwg University Local Health Board	2	2	0	Dr Colin Askill
Mid Cheshire Hospitals Foundation Trust	2	1	1	Dr Arshad Jamil
Taunton and Somerset NHS Foundation Trust	2	1	1	Dr Emma Cattell
Dorset County Hospital NHS Foundation Trust	2	2	0	Dr Lauren Gorf
Hywel Dda Health Board (Bronglais)	2	1	1	Dr Vallipuram Vigneswaran
Yeovil District Hospital NHS Foundation Trust	1	1	0	Dr Erica Beaumont
County Durham and Darlington NHS Foundation Trust	0	0	0	Dr Syed Zubair
Hywel Dda Health Board	0	0	0	Dr Elin Jones
Wye Valley NHS Trust	0	0	0	Dr Nick Reed
Salisbury NHS F Trust	0	0	0	Dr Alaaeldon Shablak
Northern Lincolnshire and Goole NHS Foundation Trust	0	0	0	Dr Georgios Bozas

eTable 2: Baseline characteristics

Used in subgroup analysis and prognostic modelling. Frailty domains are summarised in Table S2.

	CHEMO-INTENSITY pathway				CHEMO-BSC pathway	
	Level A (n=170)	Level B (n=171)	Level C (n=173)	Total (n=514)	Level C (n=23)	BSC (n=22)
**Age group (years)**						
<75	73 (43%)	73 (43%)	72 (42%)	218 (42%)	6 (26·1%)	4 (18·2%)
>=75	97 (57%)	98 (57%)	101 (58%)	296 (58%)	17 (73·9%)	18 (81·8%)
**Gender**						
Male	131 (77%)	129 (75%)	125 (72%)	385 (75%)	14 (60·9%)	13 (59·1%)
Female	39 (23%)	42 (24%)	48 (28%)	129 (25%)	9 (39·1%)	9 (40·9%)
**WHO PS**						
0-1	117 (69%)	116 (68%)	120 (70%)	353 (69%)	10 (43·5%)	7 (31·8%)
2	49 (29%)	49 (29%)	50 (29%)	148 (29%)	10 (43·5%)	13 (59·1%)
>2	4 (2·4%)	6 (3·5%)	3 (1·7%)	13 (2·5%)	3 (13·0%)	2 (9·1%)
**Distant metastases**						
No	55 (32%)	53 (31%)	52 (30%)	160 (31%)	11 (47·8%)	10 (45·5%)
Yes	115 (68%)	118 (69%)	121 (70%)	354 (69%)	12 (52·2%)	12 (54·5%)
**Tumour histology**						
Squamous	20 (12%)	18 (11%)	20 (12%)	58 (11%)	4 (17·4%)	5 (22·7%)
Other	150 (88%)	153 (90%)	153 (88%)	456 (89%)	19 (82·6%)	17 (77·3%)
**Dose reduction**						
No	159 (94%)	159 (93%)	162 (94%)	480 (93%)	23 (100·0%)	20 (90·9%)
Yes	11 (6·5%)	12 (7·0%)	11 (6·4%)	34 (6·6%)	0 (0·0%)	2 (9·1%)
**Trastuzumab**						
No	163 (95·9%)	161 (94·2%)	163 (94·2%)	487 (94·7%)	22 (95·7%)	21 (95·5%)
Yes	7 (4·1%)	10 (5·8%)	10 (5·8%)	27 (5·3%)	1 (4·3%)	1 (4·5%)
**Site of primary tumour**						
Esophagus	55 (32%)	73 (42%)	69 (40%)	197 (38%)	13 (56·5%)	9 (40·9%)
GE junction	50 (29·4%)	34 (20%)	39 (23%)	123 (24%)	4 (17·4%)	4 (18·2%)
Gastric	64 (38%)	64 (37%)	64 (37%)	192 (37%)	6 (26·1%)	9 (40·9%)
Missing	1 (0·6%)	0 (0·0%)	1 (0·6%)	2 (0·4%)		
**VAS <50 (EQ-5D)**						
No	143 (84·1%)	141 (82·5%)	141 (81·5%)	425 (82·7%)	17 (73·9%)	16 (72·7%)
Yes	22 (13%)	26 (15%)	29 (17%)	77 (15%)	5 (21·7%)	4 (18·2%)
Missing	5 (2·9%)	4 (2·3%)	3 (1·7%)	12 (2·3%)	1 (4·3%)	2 (9·1%)
**GHS/QoL <41·6 (C-30)**						
No	128 (75·3%)	120 (70·2%)	129 (74·6%)	377 (73·3%)	13 (56·5%)	17 (77·3%)
Yes	36 (21%)	50 (29%)	38 (22%)	124 (24%)	8 (34·8%)	5 (22·7%)
Missing	6 (3·5%)	1 (0·6%)	6 (3·5%)	13 (2·5%)	2 (8·7%)	0 (0·0%)
**Pain (EQ-5D)**						
I have no pain or discomfort	53 (31%)	43 (25%)	58 (34%)	154 (30%)	9 (39·1%)	4 (18·2%)
I have moderate pain or discomfort	100 (58·8%)	114 (66·7%)	97 (56·1%)	311 (61%)	11 (47·8%)	16 (72·7%)
I have extreme pain or discomfort	11 (6·5%)	7 (4·1%)	14 (8·1%)	32 (6·2%)	2 (8·7%)	2 (9·1%)
Missing	6 (3·5%)	7 (4·1%)	4 (2·3%)	17 (3·3%)	1 (4·3%)	0 (0·0%)
**Dysphagia (OG-25)**						
No	57 (34%)	45 (26%)	45 (26%)	147 (29%)	6 (26·1%)	7 (31·8%)
Yes	108 (64%)	124 (73%)	124 (72%)	356 (69%)	16 (69·6%)	15 (68·2%)
Missing	5 (2·9%)	2 (1·2%)	4 (2·3%)	11 (2·1%)	1 (4·3%)	0 (0·0%)
**Baseline Odynophagia (OG-25)**						
No	65 (38%)	63 (37%)	64 (37%)	192 (37%)	10 (43·5%)	9 (40·9%)
Yes	99 (58%)	106 (62%)	105 (61%)	310 (60%)	12 (52·2%)	13 (59·1%)
Missing	6 (3·5%)	2 (1·2%)	4 (2·3%)	12 (2·3%)	1 (4·3%)	0 (0·0%)
**Trouble with taste (OG-25)**						
No	100 (59%)	108 (63%)	101 (58%)	309 (60%)	13 (56·5%)	14 (63·6%)
Yes	65 (38%)	61 (36%)	68 (39%)	194 (38%)	9 (39·1%)	7 (31·8%)
Missing	5 (2·9%)	2 (1·2%)	4 (2·3%)	11 (2·1%)	1 (4·3%)	1 (4·5%)

© 2021 Hall PS et al. *Jama Oncol.*

	CHEMO-INTENSITY pathway				CHEMO-BSC pathway	
	Level A (n=170)	Level B (n=171)	Level C (n=173)	Total (n=514)	Level C (n=23)	BSC (n=22)
**Number of reported symptoms**						
0	35 (21%)	23 (14%)	28 (16%)	86 (17%)	3 (13·0%)	6 (27·3%)
1	30 (18%)	40 (23%)	31 (18%)	101 (20%)	5 (21·7%)	3 (13·6%)
2	57 (34%)	67 (39%)	64 (37%)	188 (37%)	10 (43·5%)	6 (27·3%)
3	42 (25%)	39 (23%)	46 (27%)	127 (25%)	4 (17·4%)	6 (27·3%)
Missing	6 (3·5%)	2 (1·2%)	4 (2·3%)	12 (2·3%)	1 (4·3%)	1 (4·5%)
**BNP > 100 or NT-Pro-BNP > 400**						
No	96 (57%)	98 (57·%)	102 (59%)	296 (58%)	11 (47·8%)	9 (40·9%)
Yes	55 (32%)	61 (36%)	56 (32%)	172 (34%)	11 (47·8%)	13 (59·1%)
Missing	19 (11%)	12 (7·0%)	15 (8·7%)	46 (8·9%)	1 (4·3%)	0 (0·0%)
**Haemoglobin <12 g/dl**						
No	94 (55%)	94 (55%)	90 (52%)	278 (54%)	12 (52·2%)	7 (31·8%)
Yes	75 (44%)	77 (45%)	82 (47%)	234 (46%)	11 (47·8%)	15 (68·2%)
Missing	1 (0·6%)	0 (0·0%)	1 (0·6%)	2 (0·4%)		
**White Cell Count > 11(*10/l)**						
No	141 (83%)	136 (80%)	141 (82%)	418 (81%)	14 (60·9%)	18 (81·8%)
Yes	29 (17%)	35 (21%)	31 (18%)	95 (19%)	9 (39·1%)	4 (18·2%)
Missing	0 (0·0%)	0 (0·0%)	1 (0·6%)	1 (0·2%)		
**Neutrophil:lymphocyte ratio > 4·0**						
No	74 (44%)	90 (53%)	85 (49%)	249 (48%)	9 (39·1%)	9 (40·9%)
Yes	96 (56·5%)	81 (47·4%)	87 (50·3%)	264 (51·4%)	14 (60·9%)	13 (59·1%)
Missing	0 (0·0%)	0 (0·0%)	1 (0·6%)	1 (0·2%)		
**Platelets > 400(*10/l)**						
No	142 (84%)	141 (83%)	138 (80%)	421 (82%)	17 (73·9%)	15 (68·2%)
Yes	28 (17%)	30 (18%)	34 (20%)	92 (18%)	6 (26·1%)	7 (31·8%)
Missing	0 (0·0%)	0 (0·0%)	1 (0·6%)	1 (0·2%)		
**GFR (formula derived) <60ml/min**						
No	136 (80%)	126 (74%)	127 (73%)	389 (76%)	19 (82·6%)	13 (59·1%)
Yes	32 (19%)	43 (25%)	42 (24%)	117 (23%)	4 (17·4%)	9 (40·9%)
Missing	2 (1·2%)	2 (1·2%)	4 (2·3%)	8 (1·6%)		
**Bilirubin >21 umol/l**						
No	164 (97%)	164 (96%)	167 (97%)	495 (96%)	23 (100·0%)	22 (100·0%)
Yes	6 (3·5%)	7 (4·1%)	5 (2·9%)	18 (3·5%)		
Missing	0 (0·0%)	0 (0·0%)	1 (0·6%)	1 (0·2%)		
**Albumin <30 g/dl**						
No	140 (82%)	139 (81%)	133 (77%)	412 (80%)	17 (73·9%)	18 (81·8%)
Yes	30 (18%)	32 (19%)	38 (22%)	100 (20%)	6 (26·1%)	4 (18·2%)
Missing	0 (0·0%)	0 (0·0%)	2 (1·2%)	2 (0·4%)		
**Urea >6 mmol/l**						
No	105 (62%)	101 (59%)	113 (65%)	319 (62%)	13 (56·5%)	13 (59·1%)
Yes	57 (34%)	64 (37%)	53 (31%)	174 (34%)	9 (39·1%)	8 (36·4%)
Missing	8 (4·7%)	6 (3·5%)	7 (4·0%)	21 (4·1%)	1 (4·3%)	1 (4·5%)
**Sodium <135 mmol/l**						
No	133 (78%)	132 (77%)	129 (75%)	394 (77%)	17 (73·9%)	17 (77·3%)
Yes	36 (21%)	39 (23%)	43 (25%)	118 (23%)	6 (26·1%)	5 (22·7%)
Missing	1 (0·6%)	0 (0·0%)	1 (0·6%)	2 (0·4%)		
**CEA >3ug/ml or Ca19-9 >37ku/l**						
No	60 (35%)	62 (36%)	62 (36%)	184 (36%)	11 (47·8%)	10 (45·5%)
Yes	104 (61%)	109 (64%)	107 (62%)	320 (62%)	12 (52·2%)	12 (54·5%)
Missing	6 (3·5%)	0 (0·0%)	4 (2·3%)	10 (1·9%)		

© 2021 Hall PS et al. *Jama Oncol.*

eTable 3: Baseline Frailty domains

Derived using the comprehensive health assessment (CHA).

	CHEMO-INTENSITY pathway			CHEMO-BSC pathway	
	**Level A (n=170)**	**Level B (n=171)**	**Level C (n=173)**	**Level C (n=23)**	**BSC (n=22)**
**Weight Loss: BMI < 18·5 (kg/m^2)**					
Yes	7 (4·1%)	13 (7·6%)	11 (6·4%)	2 (8·7%)	6 (27%)
No	163 (96%)	157 (92%)	160 (93%)	21 (91%)	15 (68%)
Missing	0 (0·0%)	1 (0·6%)	2 (1·2%)	0 (0·0%)	1 (4·5%)
**Weight Loss: 3kg or >5%**					
Yes	92 (54%)	94 (55%)	85 (49%)	11 (48%)	10 (46%)
No	78 (46%)	75 (44%)	85 (49%)	12 (52%)	11 (50%)
Missing	0 (0·0%)	2 (1·2%)	3 (1·7%)	0 (0·0%)	1 (4·5%)
**Mobility: >10 seconds or unable to complete test**					
Yes	103 (61%)	91 (53%)	95 (55%)	19 (83%)	14 (66%)
No	64 (38%)	78 (46%)	75 (43%)	4 (17%)	7 (32%)
Missing	3 (1·8%)	2 (1·2%)	3 (1·7%)	0 (0·0%)	1 (4·5%)
**Falls: Has had 2 or more falls in the past 6 months**					
Yes	8 (4·7%)	9 (5·3%)	7 (4·0%)	2 (8·7%)	0 (0·0%)
No	162 (95%)	162 (95%)	166 (96%)	21 (91%)	22 (100%)
**Cognition: Mild or severe dementia diagnosis**					
Yes	22 (13%)	25 (15%)	26 (15%)	4 (17%)	3 (14%)
No	148 (87%)	145 (85%)	147 (85%)	19 (83%)	18 (82%)
Missing	0 (0·0%)	1 (0·6%)	0 (0·0%)	0 (0·0%)	1 (4·5%)
**Function: ADL/IADL**					
0	73 (43%)	74 (43%)	73 (42%)	7 (30%)	3 (14%)
1	42 (25%)	32 (19%)	43 (25%)	4 (17%)	5 (23%)
2	30 (18%)	30 (18%)	23 (13%)	4 (17%)	4 (18%)
3	13 (7·6%)	17 (9·9%)	7 (4·0%)	2 (8·7%)	5 (22·7%)
4	7 (4·1%)	7 (4·1%)	11 (6·4%)	3 (13%)	2 (9·1%)
5	4 (2·4%)	7 (4·1%)	9 (5·2%)	1 (4·3%)	2 (9·1%)
6	1 (0·6%)	4 (2·3%)	6 (3·5%)	2 (8·7%)	0 (0·0%)
7	0 (0·0%)	0 (0·0%)	1 (0·6%)	0 (0·0%)	1 (4·5%)
**Function: One or more impairment in ADL or IADL**					
Yes	97 (57%)	97 (57%)	100 (58%)	16 (70%)	19 (86%)
No	73 (43%)	74 (43%)	73 (42%)	7 (30%)	3 (14%)
**Social: Required 24 hour care**					
Yes	0 (0·0%)	2 (1·2%)	1 (0·6%)		
No	168 (99%)	168 (98%)	171 (99%)	23 (100%)	21 (96%)
Missing	2 (1·2%)	1 (0·6%)	1 (0·6%)	0 (0·0%)	1 (4·5%)
**Mood: Extremely anxious or depressed**					
Yes	2 (1·2%)	4 (2·3%)	3 (1·7%)		
No	163 (96%)	160 (94%)	166 (96%)	21 (91%)	22 (100%)
Missing	5 (2·9%)	7 (4·1%)	4 (2·3%)	2 (8·7%)	0 (0·0%)
**Fatigue: 'Very much for either needing to rest or was tired' or**					

© 2021 Hall PS et al. *Jama Oncol.*

	CHEMO-INTENSITY pathway			CHEMO-BSC pathway	
	**Level A (n=170)**	**Level B (n=171)**	**Level C (n=173)**	**Level C (n=23)**	**BSC (n=22)**
**'Quite a bit for both questions'**					
Yes	42 (25%)	42 (25%)	42 (24%)	5 (22%)	7 (32%)
No	117 (69%)	126 (74%)	121 (70%)	15 (65%)	15 (68%)
Missing	11 (6·5%)	3 (1·8%)	10 (5·8%)	3 (13·0%)	0 (0·0%)
**Polypharmacy: 5 or more prescribed regular medications**					
Yes	127 (75%)	129 (75%)	116 (67%)	19 (83%)	14 (64%)
No	42 (25%)	41 (24%)	56 (32%)	4 (17%)	7 (32%)
Missing	1 (0·6%)	1 (0·6%)	1 (0·6%)	0 (0·0%)	1 (4·5%)

© 2021 Hall PS et al. *Jama Oncol.*

eTable 4a Summaries of missing data for quality of life questionnaires in CHEMO-INTENSITY pathway (Percentage out of total expected questionnaires)

	EORTC-QLQ-C30			Fatigue			EQ-5D-3L		
**QoL time point**	**Number not missing**	**Number missing**	**Percentage missing**	**Number not missing**	**Number missing**	**Percentage missing***	**Number not missing**	**Number missing**	**Percentage missing**
Baseline	499	15	2.92	499	15	2.92	490	24	4.67
Cycle 1				279	213	43.3			
Cycle 2				227	243	51.7			
9 weeks / Cycle 3	230	121	34.5	314	121	27.8	297	138	31.7
Cycle 4				160	246	60.6			
Cycle 5				148	232	61.1			
18 weeks / Cycle 6				208	146	41.2	210	156	42.6
27 weeks				161	115	41.7	163	113	40.9
36 weeks				115	90	43.9	115	90	43.9
52 weeks				47	42	47.2	48	41	46.1

*Percentage out of total expected questionnaires

eTable 4b Summaries of missing data for quality of life questionnaires in the CHEMO-BSC pathway

	Fatigue			EQ-5D-3L		
**QoL time point**	**Number not missing**	**Number missing**	**Percentage missing**	**Number not missing**	**Number missing**	**Percentage missing**
Baseline	43	2	4.44	43	2	4.44
Cycle 1	4	28	87.5			
Cycle 2	2	25	92.6			
9 weeks / Cycle 3	14	9	39.1	12	11	47.8
Cycle 4	1	19	95.0			
Cycle 5	1	15	93.8			
18 weeks / Cycle 6	8	7	46.7	8	7	46.7
27 weeks	2	7	77.8	2	7	77.8
36 weeks	1	2	66.7	1	2	66.7
52 weeks	1	0	0	1	0	0

*Percentage out of total expected questionnaires

eTable 5: EORTC-QLQ-C30 Global health status in the CHEMO-INTENSITY pathway

© 2021 Hall PS et al. *Jama Oncol.*

(mean and 95% Confidence Intervals for baseline, adjusted mean and 95% Confidence Intervals for 9 weeks)

	Level A	Level B	Level C
**Global health status**			
Baseline	60 (57, 63)	57 (54, 61)	61 (58, 64)
9 weeks	60 (57, 64)	63 (59, 66)	66 (62, 69)

eTable 6: EORTC-QLQ-C30 Functional scales in the CHEMO-INTENSITY pathway

(mean and 95% Confidence Intervals for baseline, adjusted mean and 95% Confidence Intervals for 9 weeks)

	Level A	Level B	Level C
**Physical functioning**			
Baseline	76 (73, 79)	73 (70, 76)	74 (71, 77)
9 weeks	65 (62, 68)	67 (63, 70)	71 (68, 74)
**Role functioning**			
Baseline	7 (69, 77)	68 (63, 72)	68 (63, 73)
9 weeks	61 (57, 65)	66 (61, 70)	70 (66, 74)
**Emotional functioning**			
Baseline	79 (76, 81)	76 (73, 80)	77 (73, 80)
9 weeks	80 (77, 83)	78 (75, 81)	81 (78, 84)
**Cognitive functioning**			
Baseline	86 (83, 89)	84 (81, 88)	85 (82, 88)
9 weeks	80 (76, 83)	83 (80, 86)	83 (80, 86)
**Social functioning**			
Baseline	76 (71, 80)	71 (66, 75)	72 (67, 76)
9 weeks	68 (63, 72)	75 (70, 80)	77 (73, 82)

eTable 7: EORTC-QLQ-C30 Symptom scales in the CHEMO-INTENSITY pathway

(mean and 95% Confidence Intervals for baseline, adjusted mean and 95% Confidence Intervals for 9 weeks)

	Level A	Level B	Level C
**Fatigue**			
Baseline	38 (34, 42)	42 (38, 46)	39 (35, 43)
9 weeks	46 (42, 50)	45 (41, 49)	37 (33, 41)
**Nausea / Vomiting**			
Baseline	15 (11, 18)	17 (14, 21)	17 (14, 21)
9 weeks	17 (13, 20)	11 (7·5, 14)	11 (8, 14)
**Pain**			
Baseline	29 (24, 33)	24 (20, 28)	25 (21, 29)
9 weeks	17 (13, 21)	20 (16, 24)	17 (13, 20)

© 2021 Hall PS et al. *Jama Oncol.*

eTable 8: EORTC-QLQ-C30 Symptom scales - CHEMO-INTENSITY pathway
(mean and 95% Confidence Intervals for baseline, adjusted mean and 95% Confidence Intervals for 9 weeks)

	Level A	Level B	Level C
**Dysponea**			
Baseline	24 (20, 28)	27 (22, 31)	23 (18, 27)
9 weeks	25 (21, 30)	30 (26, 34)	20 (16, 24)
**Insomnia**			
Baseline	29 (24, 34)	28 (24, 33)	30 (25, 35)
9 weeks	24 (19, 29)	27 (22, 32)	27 (22, 31)
**Appetite loss**			
Baseline	41 (36, 47)	46 (41, 51)	43 (37, 48)
9 weeks	33 (28, 38)	32 (27, 37)	27 (22, 32)
**Constipation**			
Baseline	31 (26, 35)	28 (23, 32)	23 (19, 28)
9 weeks	22 (18, 26)	15 (11, 20)	17 (13, 21)
**Diarrhoea**			
Baseline	12 (8·3, 15)	6·1 (3·8, 8·4)	9·7 (6·5, 13)
9 weeks	15·1 (11, 19)	12 (8·3, 16)	11 (7·6, 14·9)
**Financial Problems**			
Baseline	7·6 (4·47, 10·8)	8·8 (5·5, 12)	8·9 (5·75, 12)
9 weeks	5·7 (3·33, 8·15)	4·9 (2·5, 7·3)	3·7 (1·3, 6·0)

eTable 9: EORTC-QLQ-C30 Fatigue scale in the CHEMO-INTENSITY pathway
(mean and 95% Confidence Intervals for baseline, adjusted mean and 95% Confidence Intervals for subsequent assessments)

	Level A	Level B	Level C
Baseline	38·4 (34·5, 42·3)	42·2 (38·5, 46·0)	38·8 (34·9, 42·7)
Cycle 1	44·8 (40·4, 49·2)	43·9 (39·6, 48·1)	43·8 (39·8, 47·9)
Cycle 2	48·2 (43·0, 53·4)	42·7 (37·4, 48·1)	41·9 (36·7, 47·0)
9 weeks / Cycle 3	47·0 (42·4, 51·5)	45·6 (41·1, 50·2)	38·3 (33·8, 42·7)
Cycle 4	44·7 (38·7, 50·7)	47·7 (41·7, 53·7)	48·8 (43·2, 54·4)
Cycle 5	43·8 (37·6, 50·0)	48·4 (42·2, 54·7)	45·0 (39·4, 50·6)
18 weeks / Cycle 6	46·7 (41·0, 52·3)	51·0 (45·5, 56·5)	48·0 (42·8, 53·1)
27 weeks	48·8 (42·0, 55·7)	56·0 (49·3, 62·6)	54·5 (48·0, 61·0)
36 weeks	46·1 (38·8, 53·5)	63·9 (56·9, 71·0)	49·4 (42·4, 56·3)
52 weeks	64·8 (53·6, 76·0)	63·0 (51·2, 74·8)	59·7 (48·6, 70·8)

© 2021 Hall PS et al. *Jama Oncol.*

eTable 10: EORTC-OG-25 Symptom scales in the CHEMO-INTENSITY pathway

(mean and 95% Confidence Intervals for baseline, adjusted mean and 95% Confidence Intervals for subsequent assessments)

	Level A	Level B	Level C
**Dysphagia**			
Baseline	22·1 (18·5, 25·7)	22·5 (19·2, 25·8)	22·2 (19·0, 25·3)
9 weeks	11·4 (8·8, 14·0)	12·3 (9·7, 15·0)	9·7 (7·2, 12·3)
**Odynophagia**			
Baseline	22·5 (18·7, 26·2)	23·5 (19·4, 27·5)	23·6 (19·6, 27·6)
9 weeks	11·1 (8·2, 14·0)	10·9 (78·0, 13·8)	10·2 (7·3, 13·1)
**Pain and discomfort**			
Baseline	26·9 (22·7, 31·1)	27·0 (22·92, 31·05)	27·0 (22·78, 31·26)
9 weeks	15·8 (12·4, 19·1)	16·2 (12·82, 19·49)	18·4 (15·08, 21·65)
**Trouble with taste**			
Baseline	21·4 (16·6, 26·2)	18·7 (14·4, 23·1)	19·3 (15·2, 23·5)
9 weeks	26·5 (21·6, 31·3)	23·9 (19·0, 28·7)	20·2 (15·4, 25·0)

© 2021 Hall PS et al. *Jama Oncol.*

eTable 11: Summaries of best response

By allocated treatment and differences between standard and experimental groups with exact 90% confidence intervals.

	Level A (n=125)	Level B (n=112)	Level C (n=112)	Level A vs. Level B	Level A vs. Level C
**RECIST response at week 9-52**					
Responses: CR + PR	59 (47%) (40, 55)	32 (29%) (22, 36)	35 (31%) (24, 39)	19% (8 to 29)	16% (5 to 26)
Disease control: CR + PR + SD	86 (69%) (61, 76)	69 (62%) (53, 69)	70 (63%) (54, 70)	7·2% (-3·5 to 17·8)	6·3% (-4·4 to 17)
Complete response	6 (4·8%) (2, 9)	5 (4·5%) (2, 9)	5 (4·5%) (2, 9)	0·3% (-10·3 to 11·0)	0·3% (-10 to 11)
Partial response	53 (42%) (35, 50)	27 (24%) (18, 32)	30 (27%) (20, 35)	18% (7·6 to 29)	16% (4·9 to 26)
Stable disease	27 (22%) (16, 29)	37 (33%) (26, 41)	35 (31%) (24, 39)	-11% (-21·9 to -0·7)	-9·7% (-20 to 1·1)
Progressive disease	13 (10%) (6·3, 16)	18 (16%) (11, 23)	13 (12%) (7·0, 18)	-5·7% (-16·3 to 5·0)	-1·2% (-12 to 10)
Non measureable disease	1 (0·8%) (0·1, 3·7)	0 (0·0%) (0·0, 2·6)	2 (1·8%) (0·3, 5·5)	0·8% (-9·9 to 11·5)	-1·0% (-12 to 8)
No assessment	25 (20%) (14, 26)	25 (22%) (16, 30)	27 (24%) (18, 32)	-2·3% (-13·0 to 8·4)	-4·1% (-15 to 7)

eTable 12: Factors Associated with OTU outcome

(multivariable analysis)

	Odds of a worse outcome (95% CI)	Chi-sq	Degrees of freedom	p value
**Frailty**				
Severely frail (≥3) vs. Not/Slightly frail (0-2)	1·44 (1·03-2·02)	4·51	1	0·034
**Baseline VAS <50 (EQ-5D)**				
Yes vs. No	1·69 (1·06- 2·69)	4·87	1	0·027
**Neutrophil:lymphocyte ratio > 4·0**				
Yes vs. No	1·59 (1·14, 2·20)	7·65	1	0·006

© 2021 Hall PS et al. *Jama Oncol.*

eTable 13: Lookup table for multivariable prognostic model·

Baseline Frailty	Baseline QL (EQ-5D VAS)	Baseline NLR	Predicted class probabilities		
			probability of Good OTU	probability of Intermediate OTU	probability of Poor OTU
Not/Slightly frail (0-2)	VAS score ≥50	NLR ≤4·0	0·44	0·29	0·27
Not/Slightly frail (0-2)	VAS score ≥50	***NLR >4·0***	0·35	0·30	0·36
Not/Slightly frail (0-2)	***VAS score <50***	NLR ≤4·0	0·33	0·30	0·38
Severely frail (≥3)	VAS score ≥50	NLR ≤4·0	0·35	0·30	0·35
Not/Slightly frail (0-2)	***VAS score <50***	***NLR >4·0***	0·24	0·28	0·47
Severely frail (≥3)	VAS score ≥50	***NLR >4·0***	0·26	0·29	0·45
Severely frail (≥3)	***VAS score <50***	NLR ≤4·0	0·25	0·28	0·47
Severely frail (≥3)	***VAS score <50***	***NLR >4·0***	0·18	0·25	0·57

QL=quality of life; VAS=visual analogue scale (≥50 = better QL; <50 = worse QL); NLR=neutrophil:lymphocyte ratio; OTU=overall treatment utility
Adverse baseline characteristics shown in ***bold italics***

© 2021 Hall PS et al. *Jama Oncol.*

eTable 14: Predictive ability of the multivariable prognostic model evaluated at each variable combination

Normalised linear predictor	Linear predictor corresponds to:			Odds of a worse outcome (95% CI)		Predicted class probabilities			
	Baseline Frailty	Baseline QL (EQ-5D VAS)	Baseline NLR ratio	Level B vs. Level A	Level C vs. Level A	Allocation	P(Good OTU)	P(Intermediate OTU)	P(Poor OTU)
1	Not/Slightly frail (0-2)	VAS score ≥50 (better QL)	NLR ≤4·0	1·01 (0·50-2·00)	0·33 (0·16-0·68)	Level A	0·41	0·30	0·29
						Level B	0·41	0·30	0·29
						Level C	0·68	0·20	0·12
0·698	Severely frail (≥3)	VAS score ≥50 (better QL)	NLR ≤4·0	1·13 (0·74-1·73)	0·62 (0·40-0·94)	Level A	0·38	0·30	0·32
						Level B	0·35	0·30	0·35
						Level C	0·50	0·28	0·22
0·685	Not/Slightly frail (0-2)	VAS score ≥50 (better QL)	NLR >4·0	1·14 (0·75-1·73)	0·63 (0·42-0·96)	Level A	0·38	0·30	0·32
						Level B	0·35	0·30	0·35
						Level C	0·49	0·30	0·23
0·617	Not/Slightly frail (0-2)	VAS score <50 (worse QL)	NLR ≤4·0	1·17 (0·79-1·74)	0·73 (0·49-1·08)	Level A	0·37	0·30	0·33
						Level B	0·33	0·30	0·36
						Level C	0·45	0·29	0·26
0·383	Severely frail (≥3)	VAS score ≥50 (better QL)	NLR >4·0	1·29 (0·78-2·13)	1·17 (0·72-1·90)	Level A	0·35	0·30	0·35
						Level B	0·29	0·30	0·41
						Level C	0·31	0·30	0·39
0·315	Severely frail (≥3)	VAS score <50 (worse QL)	NLR ≤4·0	1·32 (0·75-2·33)	1·35 (0·78-2·33)	Level A	0·34	0·03	0·36
						Level B	0·28	0·30	0·42
						Level C	0·28	0·30	0·43
0·302	Not/Slightly frail (0-2)	VAS score <50 (worse QL)	NLR >4·0	1·33	1·38 (0·79-2·43)	Level A	0·34	0·30	0·36

© 2021 Hall PS et al. *Jama Oncol.*

Normalised linear predictor	Linear predictor corresponds to:			Odds of a worse outcome (95% CI)		Predicted class probabilities			
	Baseline Frailty	Baseline QL (EQ-5D VAS)	Baseline NLR ratio	Level B vs. Level A	Level C vs. Level A	Allocation	P(Good OTU)	P(Intermediate OTU)	P(Poor OTU)
				(0·74-2·38)		Level B	0·28	0·30	0·43
						Level C	0·27	0·30	0·44
00	Severely frail (≥3)	VAS score <50 (worse QL)	NLR >4·0	1·50 (0·59-3·83)	2·56 (1·02-6·46)	Level A	0·31	0·30	0·39
						Level B	0·23	0·28	0·49
						Level C	0·15	0·23	0·62

eTable 15 Chemotherapy delivery and tolerability (CHEMO-INTENSITY randomisation)

[Note that participants who did not receive any treatment were excluded so only 492 participants were included in this table

	Level A	Level B	Level C
n	170	171	173
Started treatment	161	163	168
Required dose-reduction for toxicity (cycle 1-3)	63 (39%)	39 (24%)	21 (13%)
Stopped for toxicity (cycle 1-3)	53 (33%)	47 (29%)	34 (20%)
Any of the above (dose-reduction or stop)	104 (65%)	79 (49%)	51 (30%)
Tolerated treatment but stopped before cycle 3 because of cancer progression or death	18 (11%)	31 (19%)	31 (19%)
Received 3 cycles at intended dose without delay (>1 week)	51 (32%)	72 (44%)	97 (58%)

© 2021 Hall PS et al. *Jama Oncol.*

Supplementary Figures

eFigure 1. Influence of baseline factors and dose level on OTU

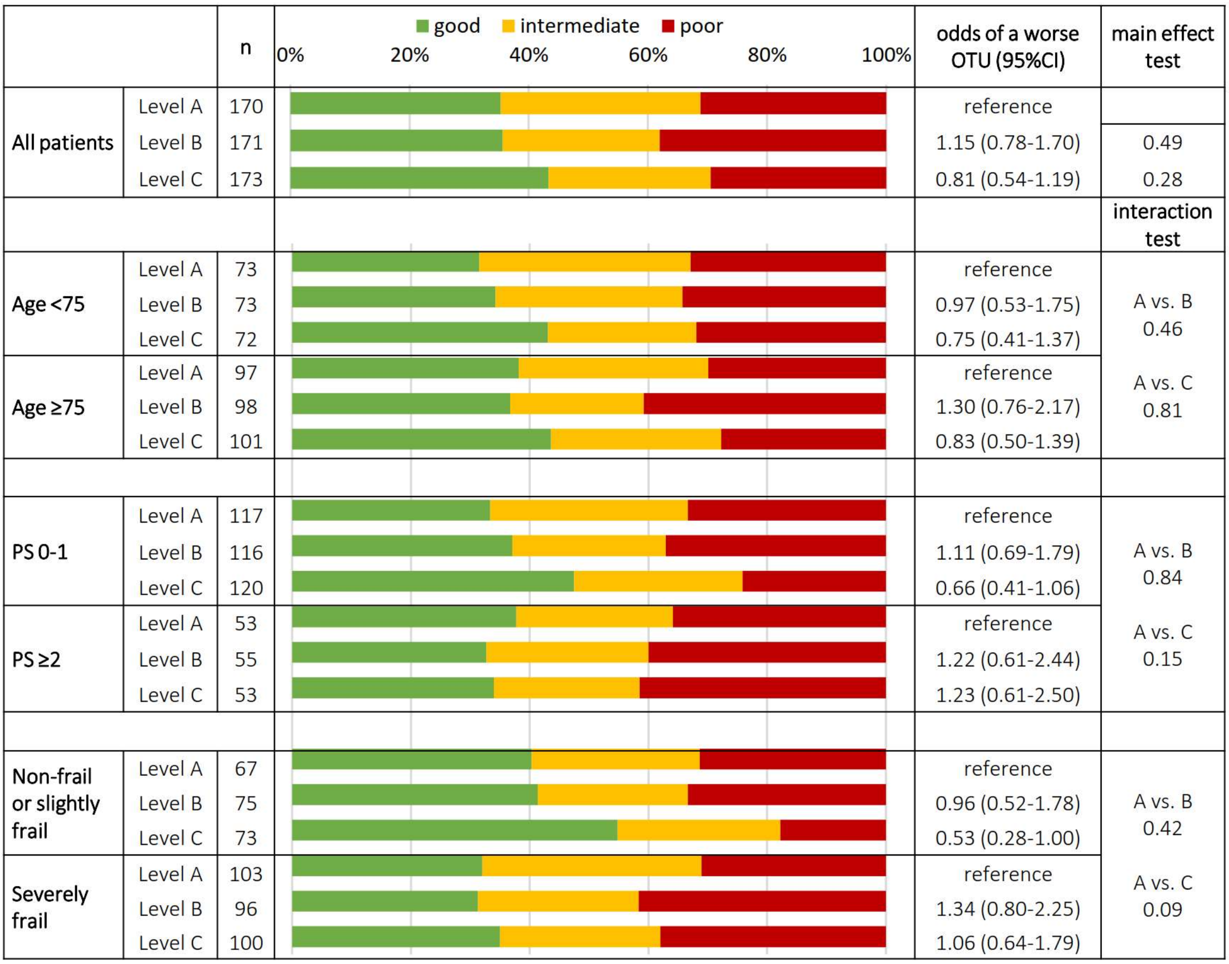

		n	odds of a worse OTU (95%CI)	main effect test
All patients	Level A	170	reference	
	Level B	171	1.15 (0.78-1.70)	0.49
	Level C	173	0.81 (0.54-1.19)	0.28
				interaction test
Age <75	Level A	73	reference	A vs. B 0.46
	Level B	73	0.97 (0.53-1.75)	
	Level C	72	0.75 (0.41-1.37)	
Age ≥75	Level A	97	reference	A vs. C 0.81
	Level B	98	1.30 (0.76-2.17)	
	Level C	101	0.83 (0.50-1.39)	
PS 0-1	Level A	117	reference	A vs. B 0.84
	Level B	116	1.11 (0.69-1.79)	
	Level C	120	0.66 (0.41-1.06)	
PS ≥2	Level A	53	reference	A vs. C 0.15
	Level B	55	1.22 (0.61-2.44)	
	Level C	53	1.23 (0.61-2.50)	
Non-frail or slightly frail	Level A	67	reference	A vs. B 0.42
	Level B	75	0.96 (0.52-1.78)	
	Level C	73	0.53 (0.28-1.00)	
Severely frail	Level A	103	reference	A vs. C 0.09
	Level B	96	1.34 (0.80-2.25)	
	Level C	100	1.06 (0.64-1.79)	

This analysis includes all 514 patients in the CHEMO-INTENSITY pathway. All odds ratios are adjusted for the minimisation stratification factors (age, PS, metastases, histology (squamous/other), renal function, planned use of trastuzumab). Tests for interaction are a one degree of freedom test for two-category subgroups which sought treatment effect heterogeneity.

© 2021 Hall PS et al. *Jama Oncol.*

eFigure 2. Univariate analysis: impact of baseline variables Overall Treatment Utility

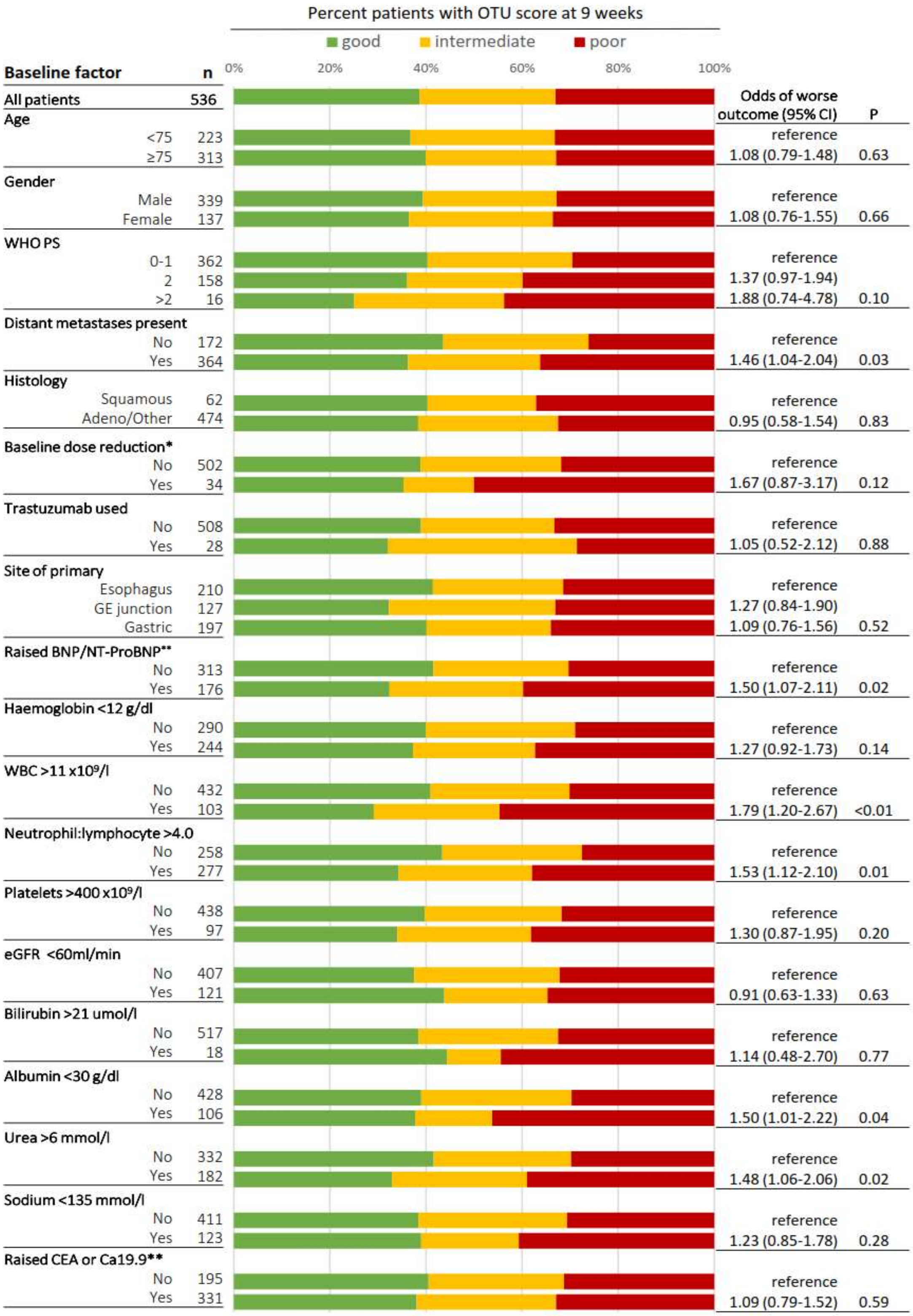

This analysis includes all patients allocated chemotherapy (all 514 in the CHEMO-INTENSITY plus 23 in the CHEMO-BSC pathway). Patients allocated BSC alone are not included as OTU cannot be scored in patients not allocated chemotherapy. *A baseline capecitabine dose reduction of 25% was mandated for patients with eGFR 30-50ml/min or bilirubin 1.5-2 xULN. Patients with eGFR <30 or bili >2xULN were not eligible. ** "Raised" defined as: BNP>100 pg/ml, NT-proBNP >400 pg/ml, CEA >3 ug/ml, Ca19.9 >37 kU/l

eFigure 3 Univariate analysis : Impact of baseline symptoms Overall Treatment Utility

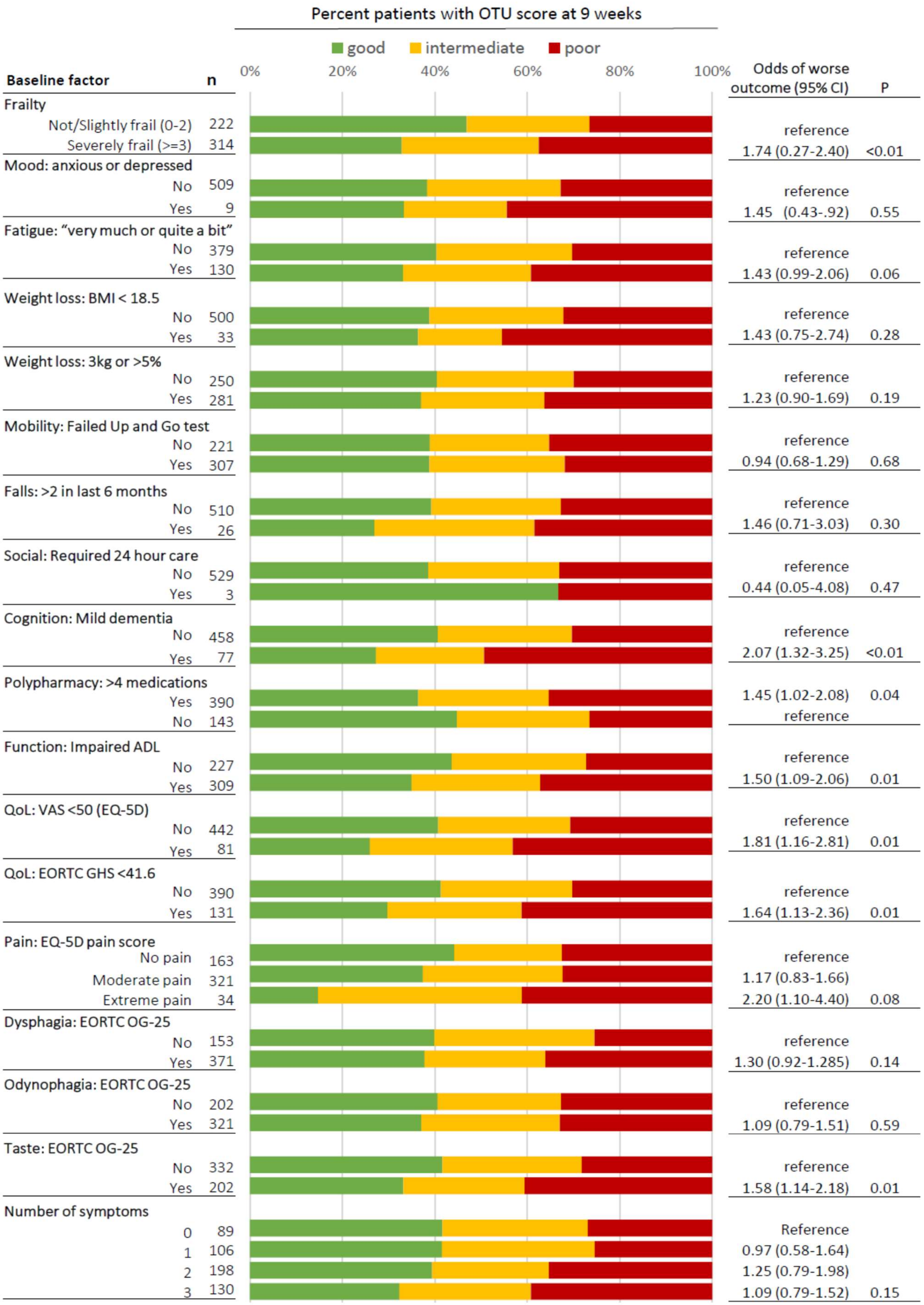

eFigure 4: Forest plot for CHEMO-INTENSITY PFS (Level A vs. Level B)

© 2021 Hall PS et al. *Jama Oncol.*

Subgroup	Category	Level A n/N	Level B n/N	HR (90%CI)	P. (het)
Age	<75	62/73	66/73	1.27 (0.92, 1.74)	0.4483
	>=75	80/97	81/98	0.98 (0.75, 1.28)	
Gender	Male	113/131	111/129	0.98 (0.78, 1.23)	0.1097
	Female	29/39	36/42	1.50 (0.98, 2.30)	
Distant metastases	Yes	99/115	103/118	1.08 (0.85, 1.36)	0.7819
	No	43/55	44/53	1.07 (0.75, 1.53)	
Tumour histology	Squamous	15/20	17/18	0.91 (0.47, 1.74)	0.9659
	Other	127/150	130/153	1.08 (0.87, 1.33)	
Dose reduction	Yes	9/11	11/12	0.94 (0.39, 2.29)	0.8138
	No	133/159	136/159	1.07 (0.87, 1.31)	
Trastuzumab	Yes	5/7	9/10	0.30 (0.08, 1.10)	0.1575
	No/Not Decided	137/163	138/161	1.09 (0.90, 1.34)	
WHO PS	0-1	97/117	98/116	1.30 (1.02, 1.66)	0.0581
	>=2	45/53	49/55	0.78 (0.55, 1.11)	
Site of primary tumour	Oesophagus	44/55	66/73	1.12 (0.80, 1.55)	0.9646
	GO junction	42/50	30/34	1.09 (0.70, 1.68)	
	Gastric	55/64	51/64	1.08 (0.77, 1.51)	
Mood	Yes	2/2	2/4		0.586
	No	136/163	139/160	1.09 (0.89, 1.34)	
Fatigue	Yes	34/42	39/42	1.35 (0.85, 2.14)	0.6521
	No	98/117	105/126	1.03 (0.81, 1.31)	
Weight Loss: BMI < 18.5	Yes	5/7	12/13	0.43 (0.09, 2.14)	0.8195
	No	137/163	135/157	1.07 (0.88, 1.32)	
Weight Loss	Yes	77/92	86/94	1.28 (0.98, 1.67)	0.1108
	No	65/78	61/75	0.86 (0.64, 1.17)	
Mobility	Yes	83/103	78/91	1.10 (0.83, 1.46)	0.5377
	No	56/64	69/78	0.93 (0.68, 1.28)	
Falls	Yes	7/8	8/9	0.50 (0.14, 1.86)	0.6573
	No	135/162	139/162	1.08 (0.88, 1.33)	
Social	Yes	0/0	1/2		0.9752
	No	141/168	146/168	1.09 (0.89, 1.32)	
Cognition	Yes	20/22	22/25	2.52 (1.29, 4.94)	0.2686
	No	122/148	125/145	1.03 (0.83, 1.28)	
Polypharmacy	Yes	104/127	109/129	1.02 (0.81, 1.29)	0.4342
	No	38/42	38/41	1.34 (0.89, 2.01)	
Function	Yes	80/97	87/97	1.19 (0.91, 1.55)	0.6701
	No	62/73	60/74	1.00 (0.73, 1.37)	
Frailty	No (0-1)	21/23	24/30	0.68 (0.38, 1.24)	0.4352
	Slightly (2)	35/44	36/45	1.07 (0.70, 1.63)	
	Severely (>=3)	86/103	87/96	1.25 (0.95, 1.63)	
Frailty	Not/Slightly frail (0-2)	56/67	60/75	0.91 (0.66, 1.25)	0.216
	Severely (>=3)	86/103	87/96	1.25 (0.95, 1.63)	
Baseline VAS <50 (EQ-5D)	Yes	18/22	23/26	1.03 (0.54, 1.95)	0.6154
	No	120/143	120/141	1.06 (0.86, 1.32)	
Baseline GHS/QoL <41.6 (C-30)	Yes	29/36	46/50	1.04 (0.67, 1.64)	0.9452
	No	108/128	100/120	1.03 (0.82, 1.30)	
Baseline Pain (EQ-5D)	No pain	40/53	32/43	1.19 (0.77, 1.85)	0.5342
	Moderate pain	86/100	102/114	1.08 (0.83, 1.39)	
	Extreme pain	11/11	7/7	1.51 (0.50, 4.50)	
Baseline Dysphagia (OG-25)	Yes	91/108	111/124	1.15 (0.91, 1.46)	0.2695
	No	47/57	34/45	0.76 (0.50, 1.14)	
Baseline Odynophagia (OG-25)	Yes	85/99	95/106	1.02 (0.79, 1.32)	0.926
	No	52/65	50/63	1.06 (0.76, 1.48)	
Baseline Trouble with taste (OG-25)	Yes	56/65	55/61	1.09 (0.78, 1.51)	0.9726
	No	82/100	90/108	1.08 (0.84, 1.40)	
BNP > 100 or NT-Pro-BNP > 400	Yes	46/53	52/59	1.22 (0.87, 1.72)	0.7102
	No	81/98	85/100	1.10 (0.85, 1.43)	
Haemoglobin <12 g/dl	Yes	59/75	69/77	1.44 (1.06, 1.96)	0.0185
	No	82/94	78/94	0.80 (0.61, 1.05)	
White Cell Count > 11(*10^9/l)	Yes	24/29	32/35	1.48 (0.86, 2.56)	0.3678
	No	118/141	115/136	1.03 (0.83, 1.29)	
Neutrophil:lymphocyte ratio > 4.0	Yes	85/96	71/81	1.09 (0.83, 1.43)	0.9673
	No	57/74	76/90	1.13 (0.84, 1.53)	
Platelets > 400(*10^9/l)	Yes	23/28	29/30	1.26 (0.75, 2.13)	0.2459
	No	119/142	118/141	1.02 (0.82, 1.26)	
GFR (formula derived) <60ml/min	Yes	25/32	40/43	1.40 (0.89, 2.18)	0.3215
	No	115/136	106/126	1.01 (0.81, 1.27)	
Bilirubin >21 umol/l	Yes	4/6	5/7	1.67 (0.08, 36.75)	0.2712
	No	138/164	142/164	1.10 (0.90, 1.34)	
Albumin <30 g/dl	Yes	27/30	31/32	0.93 (0.58, 1.51)	0.8993
	No	115/140	116/139	1.08 (0.86, 1.34)	
Urea >6 mmol/l	Yes	50/57	58/64	0.97 (0.69, 1.35)	0.6876
	No	87/105	83/101	1.06 (0.81, 1.37)	
Sodium <135 mmol/l	Yes	31/36	30/39	0.89 (0.57, 1.40)	0.4698
	No	111/133	117/132	1.12 (0.90, 1.40)	
CEA >3ug/ml or Ca19-9 >37ku/l	Yes	94/104	93/109	1.06 (0.82, 1.35)	0.3216
	No	42/60	54/62	1.28 (0.91, 1.82)	
**Overall**		**142/170**	**147/171**	**1.09 (0.89, 1.32)**	

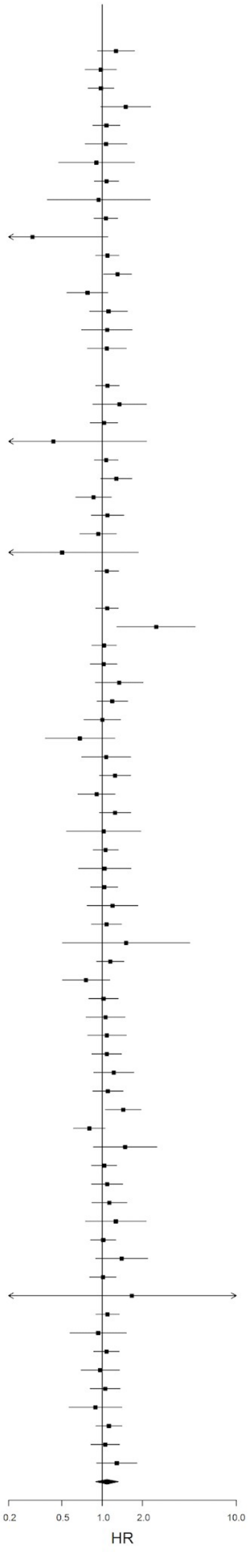

© 2021 Hall PS et al. *Jama Oncol.*

eFigure 5: Forest plot for CHEMO-INTENSITY PFS (Level A vs. Level C)

Subgroup	Category	Level A n/N	Level C n/N	HR (90%CI)	P. (het)
Age	<75	62/73	67/72	1.28 (0.95, 1.73)	0.2555
	>=75	80/97	82/101	0.96 (0.73, 1.25)	
Gender	Male	113/131	107/125	1.00 (0.79, 1.25)	0.1701
	Female	29/39	42/48	1.71 (1.11, 2.65)	
Distant metastases	Yes	99/115	111/121	1.19 (0.95, 1.51)	0.2663
	No	43/55	38/52	0.94 (0.64, 1.37)	
Tumour histology	Squamous	15/20	18/20	1.47 (0.75, 2.86)	0.2113
	Other	127/150	131/153	1.04 (0.85, 1.28)	
Dose reduction	Yes	9/11	10/11	2.70 (1.05, 6.97)	0.1115
	No	133/159	139/162	1.05 (0.86, 1.28)	
Trastuzumab	Yes	5/7	8/10	0.35 (0.10, 1.21)	0.1785
	No/Not Decided	137/163	141/163	1.14 (0.93, 1.39)	
WHO PS	0-1	97/117	102/120	1.10 (0.87, 1.39)	0.985
	>=2	45/53	47/53	1.12 (0.79, 1.59)	
Site of primary tumour	Oesophagus	44/55	57/69	1.01 (0.72, 1.42)	0.8411
	GO junction	42/50	35/39	1.58 (1.05, 2.38)	
	Gastric	55/64	56/64	1.12 (0.81, 1.55)	
Mood	Yes	2/2	2/3	1.00 (0.10, 10.24)	0.3956
	No	136/163	145/166	1.08 (0.89, 1.32)	
Fatigue	Yes	34/42	38/42	0.89 (0.59, 1.35)	0.1296
	No	98/117	103/121	1.08 (0.85, 1.37)	
Weight Loss: BMI < 18.5	Yes	5/7	9/11	15.95 (2.03, 125.68)	0.9328
	No	137/163	138/160	1.10 (0.90, 1.35)	
Weight Loss	Yes	77/92	78/85	1.33 (1.01, 1.76)	0.094
	No	65/78	69/85	0.86 (0.63, 1.16)	
Mobility	Yes	83/103	79/95	1.05 (0.80, 1.38)	0.7237
	No	56/64	67/75	1.04 (0.76, 1.42)	
Falls	Yes	7/8	6/7	0.63 (0.14, 2.78)	0.6907
	No	135/162	143/166	1.11 (0.91, 1.36)	
Social	Yes	0/0	1/1	0.63 (0.14, 2.78)	0.1006
	No	141/168	147/171	1.10 (0.90, 1.33)	
Cognition	Yes	20/22	24/26	3.51 (1.83, 6.76)	2e-04
	No	122/148	125/147	0.96 (0.78, 1.18)	
Polypharmacy	Yes	104/127	104/116	1.22 (0.97, 1.54)	0.7437
	No	38/42	44/56	0.68 (0.46, 1.01)	
Function	Yes	80/97	86/100	1.12 (0.86, 1.46)	0.9993
	No	62/73	63/73	1.08 (0.79, 1.46)	
Frailty	No (0-1)	21/23	33/41	0.82 (0.47, 1.43)	0.6581
	Slightly (2)	35/44	28/32	0.93 (0.57, 1.52)	
	Severely (>=3)	86/103	88/100	1.23 (0.95, 1.58)	
Frailty	Not/Slightly frail (0-2)	56/67	61/73	0.97 (0.71, 1.33)	0.3902
	Severely (>=3)	86/103	88/100	1.23 (0.95, 1.58)	
Baseline VAS <50 (EQ-5D)	Yes	18/22	28/29	1.73 (0.97, 3.10)	0.2307
	No	120/143	120/141	1.01 (0.81, 1.26)	
Baseline GHS/QoL <41.6 (C-30)	Yes	29/36	34/38	0.52 (0.32, 0.84)	0.1884
	No	108/128	111/129	1.14 (0.91, 1.43)	
Baseline Pain (EQ-5D)	No pain	40/53	49/58	1.22 (0.83, 1.80)	0.3994
	Moderate pain	86/100	85/97	0.91 (0.70, 1.18)	
	Extreme pain	11/11	13/14	1.76 (0.79, 3.92)	
Baseline Dysphagia (OG-25)	Yes	91/108	109/124	1.22 (0.96, 1.55)	0.0765
	No	47/57	38/45	0.75 (0.51, 1.09)	
Baseline Odynophagia (OG-25)	Yes	85/99	95/105	1.16 (0.90, 1.49)	0.446
	No	52/65	52/64	0.97 (0.70, 1.36)	
Baseline Trouble with taste (OG-25)	Yes	56/65	60/68	1.19 (0.85, 1.67)	0.7394
	No	82/100	87/101	1.02 (0.79, 1.33)	
BNP > 100 or NT-Pro-BNP > 400	Yes	46/53	47/53	1.12 (0.78, 1.59)	0.8478
	No	81/98	90/105	1.12 (0.86, 1.45)	
Haemoglobin <12 g/dl	Yes	59/75	74/82	1.54 (1.14, 2.08)	0.0124
	No	82/94	74/90	0.83 (0.63, 1.09)	
White Cell Count > 11(*10^9/l)	Yes	24/29	29/31	1.12 (0.66, 1.89)	0.76
	No	118/141	119/141	1.12 (0.90, 1.39)	
Neutrophil:lymphocyte ratio > 4.0	Yes	85/96	79/87	1.13 (0.87, 1.46)	0.9577
	No	57/74	69/85	1.09 (0.80, 1.48)	
Platelets > 400(*10^9/l)	Yes	23/28	33/34	2.03 (1.22, 3.39)	0.1097
	No	119/142	115/138	1.01 (0.81, 1.25)	
GFR (formula derived) <60ml/min	Yes	25/32	34/42	1.31 (0.82, 2.10)	0.5656
	No	115/136	111/127	1.08 (0.87, 1.36)	
Bilirubin >21 umol/l	Yes	4/6	5/5	5.15 (0.33, 79.53)	0.6499
	No	138/164	143/167	1.08 (0.89, 1.32)	
Albumin <30 g/dl	Yes	27/30	32/38	1.35 (0.83, 2.19)	0.7031
	No	115/140	115/133	1.11 (0.89, 1.39)	
Urea >6 mmol/l	Yes	50/57	48/53	0.95 (0.67, 1.35)	0.6589
	No	87/105	95/113	1.12 (0.87, 1.43)	
Sodium <135 mmol/l	Yes	31/36	39/43	1.60 (1.03, 2.50)	0.1595
	No	111/133	109/129	0.99 (0.79, 1.25)	
CEA >3ug/ml or Ca19-9 >37ku/l	Yes	94/104	95/107	1.18 (0.92, 1.51)	0.9261
	No	42/60	51/62	1.18 (0.82, 1.69)	
**Overall**		**142/170**	**149/173**	**1.10 (0.90, 1.33)**	

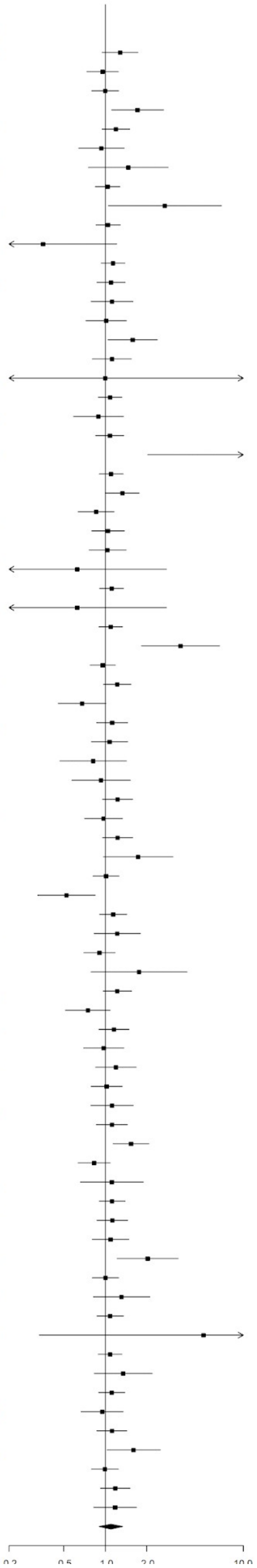

© 2021 Hall PS et al. *Jama Oncol.*

eFigure 6: Forest plot for CHEMO-INTENSITY OS (Level A vs. Level B)

Subgroup	Category	Level A n/N	Level B n/N	HR (90% CI)	P. (het)
Age	<75	56/73	54/73	0.99 (0.70, 1.40)	0.4485
	>=75	62/97	71/98	1.18 (0.87, 1.58)	
Gender	Male	92/131	99/129	1.07 (0.84, 1.37)	0.8856
	Female	26/39	26/42	1.04 (0.65, 1.67)	
Distant metastases	Yes	89/115	91/118	1.00 (0.78, 1.29)	0.1992
	No	29/55	34/53	1.36 (0.90, 2.08)	
Tumour histology	Squamous	11/20	15/18	1.16 (0.57, 2.38)	0.6433
	Other	107/150	110/153	1.06 (0.84, 1.32)	
Dose reduction	Yes	8/11	10/12	1.21 (0.48, 3.08)	0.9355
	No	110/159	115/159	1.09 (0.87, 1.36)	
Trastuzumab	Yes	4/7	8/10	0.62 (0.16, 2.44)	0.7565
	No/Not Decided	114/163	117/161	1.07 (0.86, 1.34)	
WHO PS	0-1	83/117	83/116	1.26 (0.97, 1.63)	0.148
	>=2	35/53	42/55	0.84 (0.57, 1.23)	
Site of primary tumour	Oesophagus	34/55	57/73	1.36 (0.94, 1.96)	0.5526
	GO junction	34/50	23/34	1.07 (0.66, 1.75)	
	Gastric	49/64	45/64	1.00 (0.70, 1.43)	
Mood	Yes	2/2	2/4		0.8974
	No	112/163	118/160	1.11 (0.89, 1.38)	
Fatigue	Yes	30/42	35/42	1.17 (0.73, 1.88)	0.5804
	No	79/117	87/126	1.07 (0.83, 1.40)	
Weight Loss: BMI < 18.5	Yes	5/7	9/13	0.30 (0.05, 1.93)	0.5298
	No	113/163	116/157	1.10 (0.88, 1.38)	
Weight Loss	Yes	69/92	72/94	1.16 (0.87, 1.54)	0.6048
	No	49/78	53/75	1.00 (0.71, 1.40)	
Mobility	Yes	70/103	67/91	1.09 (0.81, 1.47)	0.8566
	No	45/64	58/78	1.04 (0.73, 1.47)	
Falls	Yes	6/8	7/9	0.76 (0.20, 2.81)	0.886
	No	112/162	118/162	1.09 (0.87, 1.35)	
Social	Yes	0/0	1/2		0.9805
	No	117/168	124/168	1.09 (0.88, 1.35)	
Cognition	Yes	15/22	21/25	5.09 (2.27, 11.41)	0.0588
	No	103/148	104/145	0.99 (0.78, 1.25)	
Polypharmacy	Yes	86/127	93/129	1.07 (0.83, 1.37)	0.7244
	No	32/42	32/41	1.31 (0.84, 2.04)	
Function	Yes	69/97	74/97	1.01 (0.76, 1.34)	0.521
	No	49/73	51/74	1.13 (0.80, 1.60)	
Frailty	No (0-1)	16/23	19/30	0.74 (0.38, 1.44)	0.7584
	Slightly (2)	26/44	31/45	1.29 (0.81, 2.06)	
	Severely (>=3)	76/103	75/96	1.07 (0.81, 1.42)	
Frailty	Not/Slightly frail (0-2)	42/67	50/75	1.06 (0.74, 1.53)	0.9669
	Severely (>=3)	76/103	75/96	1.07 (0.81, 1.42)	
Baseline VAS <50 (EQ-5D)	Yes	16/22	22/26	1.40 (0.75, 2.63)	0.8652
	No	98/143	99/141	1.05 (0.83, 1.33)	
Baseline GHS/QoL <41.6 (C-30)	Yes	27/36	41/50	0.85 (0.53, 1.35)	0.5453
	No	86/128	83/120	1.08 (0.83, 1.39)	
Baseline Pain (EQ-5D)	No pain	31/53	30/43	1.87 (1.17, 3.00)	0.033
	Moderate pain	72/100	83/114	0.90 (0.68, 1.19)	
	Extreme pain	11/11	7/7	2.75 (0.93, 8.19)	
Baseline Dysphagia (OG-25)	Yes	75/108	91/124	1.11 (0.86, 1.44)	0.5124
	No	39/57	32/45	0.87 (0.56, 1.35)	
Baseline Odynophagia (OG-25)	Yes	72/99	76/106	0.87 (0.66, 1.15)	0.1235
	No	41/65	47/63	1.53 (1.07, 2.19)	
Baseline Trouble with taste (OG-25)	Yes	49/65	49/61	1.09 (0.77, 1.55)	0.9655
	No	65/100	74/108	1.10 (0.83, 1.46)	
BNP > 100 or NT-Pro-BNP > 400	Yes	40/53	51/59	1.54 (1.08, 2.19)	0.1221
	No	68/98	67/100	0.94 (0.70, 1.25)	
Haemoglobin <12 g/dl	Yes	51/75	60/77	1.37 (0.98, 1.92)	0.1281
	No	66/94	65/94	0.91 (0.67, 1.22)	
White Cell Count > 11(*10^9/l)	Yes	22/29	30/35	1.49 (0.84, 2.63)	0.4566
	No	96/141	95/136	1.05 (0.82, 1.33)	
Neutrophil:lymphocyte ratio > 4.0	Yes	72/96	62/81	1.07 (0.80, 1.44)	0.6393
	No	46/74	63/90	1.21 (0.88, 1.68)	
Platelets > 400(*10^9/l)	Yes	22/28	25/30	1.17 (0.65, 2.10)	0.3505
	No	96/142	100/141	1.04 (0.82, 1.32)	
GFR (formula derived) <60ml/min	Yes	23/32	36/43	1.33 (0.83, 2.13)	0.3337
	No	93/136	88/126	1.01 (0.78, 1.30)	
Bilirubin >21 umol/l	Yes	3/6	5/7		0.6867
	No	115/164	120/164	1.07 (0.86, 1.33)	
Albumin <30 g/dl	Yes	23/30	26/32	0.77 (0.46, 1.28)	0.3857
	No	95/140	99/139	1.15 (0.91, 1.46)	
Urea >6 mmol/l	Yes	41/57	51/64	1.00 (0.70, 1.43)	0.6741
	No	74/105	68/101	0.97 (0.73, 1.28)	
Sodium <135 mmol/l	Yes	23/36	28/39	0.99 (0.61, 1.63)	0.9972
	No	95/133	97/132	1.07 (0.84, 1.37)	
CEA >3ug/ml or Ca19-9 >37ku/l	Yes	82/104	81/109	1.01 (0.77, 1.32)	0.1163
	No	31/60	44/62	1.45 (0.98, 2.15)	
**Overall**		**118/170**	**125/171**	**1.09 (0.88, 1.36)**	

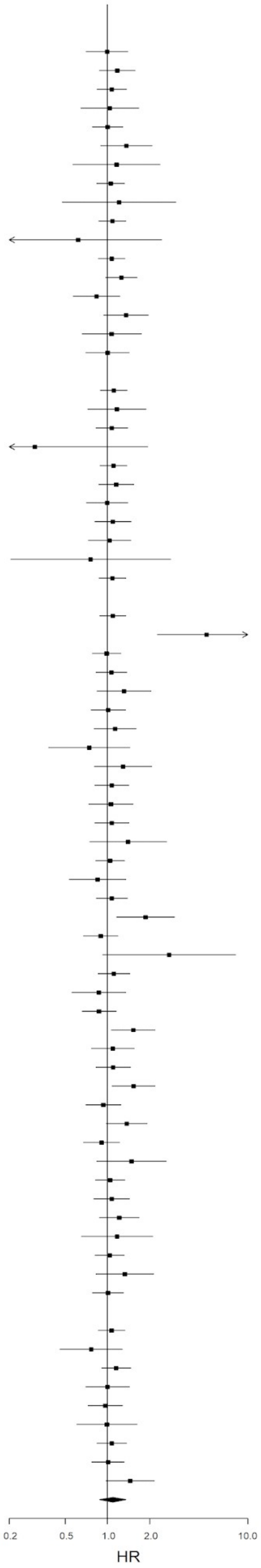

© 2021 Hall PS et al. *Jama Oncol.*

eFigure 7: Forest plot for CHEMO-INTENSITY OS (Level A vs. Level C)

Subgroup	Category	Level A n/N	Level C n/N	HR (90% CI)	P. (het)
Age	<75	56/73	63/72	1.22 (0.89, 1.66)	0.4685
	>=75	62/97	67/101	1.05 (0.78, 1.41)	
Gender	Male	92/131	96/125	1.07 (0.83, 1.36)	0.545
	Female	26/39	34/48	1.33 (0.84, 2.10)	
Distant metastases	Yes	89/115	101/121	1.13 (0.89, 1.45)	0.7494
	No	29/55	29/52	1.05 (0.67, 1.64)	
Tumour histology	Squamous	11/20	17/20	1.86 (0.89, 3.92)	0.0559
	Other	107/150	113/153	1.04 (0.83, 1.30)	
Dose reduction	Yes	8/11	10/11	1.44 (0.59, 3.53)	0.2886
	No	110/159	120/162	1.09 (0.87, 1.35)	
Trastuzumab	Yes	4/7	8/10	0.39 (0.11, 1.44)	0.7192
	No/Not Decided	114/163	122/163	1.13 (0.91, 1.41)	
WHO PS	0-1	83/117	83/120	0.95 (0.73, 1.23)	0.0553
	>=2	35/53	47/53	1.49 (1.02, 2.16)	
Site of primary tumour	Oesophagus	34/55	50/69	1.15 (0.79, 1.67)	0.9577
	GO junction	34/50	30/39	1.38 (0.88, 2.15)	
	Gastric	49/64	49/64	1.08 (0.76, 1.52)	
Mood	Yes	2/2	2/3		0.299
	No	112/163	126/166	1.13 (0.91, 1.40)	
Fatigue	Yes	30/42	37/42	0.89 (0.58, 1.36)	0.5582
	No	79/117	87/121	1.15 (0.88, 1.49)	
Weight Loss: BMI < 18.5	Yes	5/7	9/11	5.20 (0.98, 27.51)	0.3817
	No	113/163	119/160	1.11 (0.89, 1.38)	
Weight Loss	Yes	69/92	70/85	1.31 (0.98, 1.75)	0.2394
	No	49/78	58/85	0.88 (0.63, 1.24)	
Mobility	Yes	70/103	68/95	1.03 (0.77, 1.37)	0.5783
	No	45/64	60/75	1.27 (0.91, 1.79)	
Falls	Yes	6/8	6/7	0.55 (0.11, 2.75)	0.7775
	No	112/162	124/166	1.14 (0.91, 1.41)	
Social	Yes	0/0	1/1	0.55 (0.11, 2.75)	0.0439
	No	117/168	128/171	1.13 (0.91, 1.39)	
Cognition	Yes	15/22	25/26	3.08 (1.63, 5.83)	6e-04
	No	103/148	105/147	0.96 (0.76, 1.21)	
Polypharmacy	Yes	86/127	93/116	1.28 (1.00, 1.64)	0.7911
	No	32/42	36/56	0.71 (0.46, 1.09)	
Function	Yes	69/97	74/100	1.08 (0.81, 1.42)	0.8868
	No	49/73	56/73	1.13 (0.81, 1.57)	
Frailty	No (0-1)	16/23	28/41	0.82 (0.45, 1.48)	0.8152
	Slightly (2)	26/44	23/32	1.26 (0.72, 2.19)	
	Severely (>=3)	76/103	79/100	1.14 (0.87, 1.49)	
Frailty	Not/Slightly frail (0-2)	42/67	51/73	1.13 (0.79, 1.63)	0.9052
	Severely (>=3)	76/103	79/100	1.14 (0.87, 1.49)	
Baseline VAS <50 (EQ-5D)	Yes	16/22	26/29	1.96 (1.06, 3.62)	0.4295
	No	98/143	103/141	1.05 (0.83, 1.34)	
Baseline GHS/QoL <41.6 (C-30)	Yes	27/36	31/38	0.45 (0.28, 0.74)	0.0238
	No	86/128	97/129	1.26 (0.98, 1.62)	
Baseline Pain (EQ-5D)	No pain	31/53	43/58	1.49 (0.98, 2.28)	0.2443
	Moderate pain	72/100	74/97	0.90 (0.68, 1.19)	
	Extreme pain	11/11	11/14	1.30 (0.59, 2.88)	
Baseline Dysphagia (OG-25)	Yes	75/108	95/124	1.17 (0.90, 1.51)	0.4795
	No	39/57	33/45	0.95 (0.63, 1.43)	
Baseline Odynophagia (OG-25)	Yes	72/99	83/105	1.01 (0.77, 1.32)	0.4208
	No	41/65	45/64	1.17 (0.82, 1.69)	
Baseline Trouble with taste (OG-25)	Yes	49/65	54/68	0.99 (0.69, 1.41)	0.8569
	No	65/100	74/101	1.09 (0.82, 1.45)	
BNP > 100 or NT-Pro-BNP > 400	Yes	40/53	41/53	1.16 (0.80, 1.69)	0.7346
	No	68/98	78/105	1.02 (0.77, 1.36)	
Haemoglobin <12 g/dl	Yes	51/75	63/82	1.31 (0.96, 1.80)	0.342
	No	66/94	66/90	0.98 (0.73, 1.31)	
White Cell Count > 11(*10^9/l)	Yes	22/29	25/31	1.06 (0.62, 1.83)	0.462
	No	96/141	104/141	1.18 (0.93, 1.49)	
Neutrophil:lymphocyte ratio > 4.0	Yes	72/96	74/87	1.16 (0.88, 1.53)	0.9315
	No	46/74	55/85	1.15 (0.82, 1.62)	
Platelets > 400(*10^9/l)	Yes	22/28	28/34	2.02 (1.14, 3.57)	0.4406
	No	96/142	101/138	1.07 (0.84, 1.36)	
GFR (formula derived) <60ml/min	Yes	23/32	32/42	1.26 (0.77, 2.04)	0.8375
	No	93/136	95/127	1.01 (0.79, 1.29)	
Bilirubin >21 umol/l	Yes	3/6	5/5	5.36 (0.27, 105.92)	0.1869
	No	115/164	124/167	1.09 (0.88, 1.35)	
Albumin <30 g/dl	Yes	23/30	28/38	1.10 (0.64, 1.88)	0.5896
	No	95/140	100/133	1.15 (0.90, 1.46)	
Urea >6 mmol/l	Yes	41/57	41/53	0.93 (0.63, 1.38)	0.7251
	No	74/105	83/113	1.07 (0.82, 1.41)	
Sodium <135 mmol/l	Yes	23/36	35/43	1.65 (1.01, 2.70)	0.1121
	No	95/133	94/129	0.99 (0.78, 1.26)	
CEA >3ug/ml or Ca19-9 >37ku/l	Yes	82/104	88/107	1.17 (0.90, 1.51)	0.7179
	No	31/60	40/62	1.26 (0.84, 1.90)	
**Overall**		**118/170**	**130/173**	**1.14 (0.92, 1.41)**	

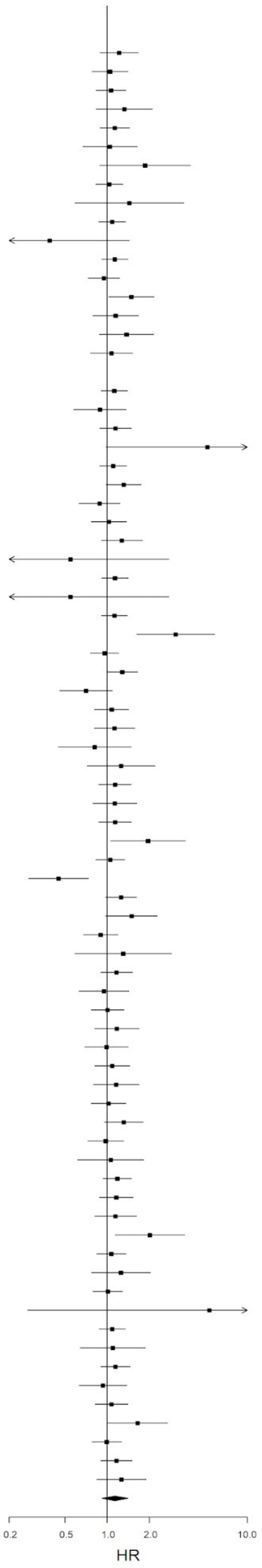

© 2021 Hall PS et al. *Jama Oncol.*

eFigure 8: EORTC-QLQ-C30 Fatigue scale in the CHEMO-INTENSITY pathway
(Mean and 95% Confidence Intervals for baseline, adjusted mean and 95% Confidence Intervals for subsequent assessments)

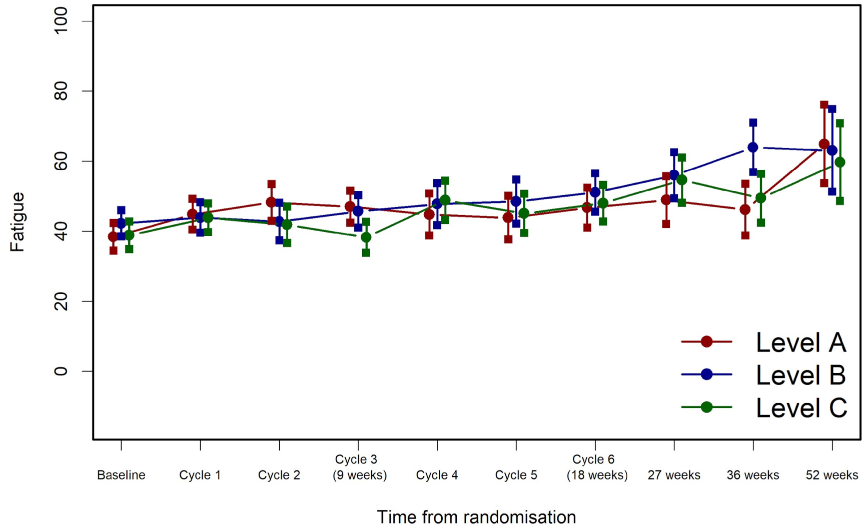

eFigure 9: EuroqoL EQ-5D-3L Score in the CHEMO-INTENSITY pathway
(mean and 95% Confidence Intervals for baseline, adjusted mean and 95% Confidence Intervals for subsequent assessments)

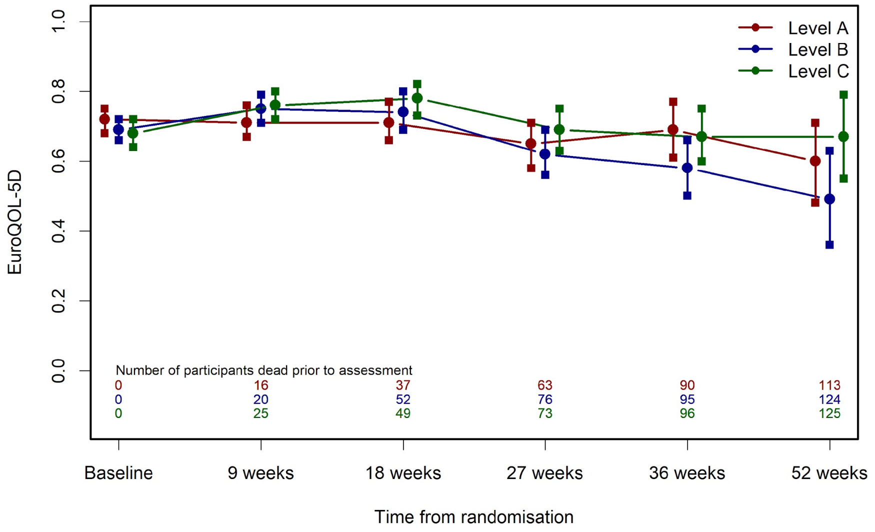

© 2021 Hall PS et al. *Jama Oncol.*

eFigure 10: EORTC-QLQ-C30 Fatigue scale in the CHEMO-BSC pathway
(mean and 95% Confidence Intervals for baseline, adjusted mean and 95% Confidence Intervals for subsequent assessments)

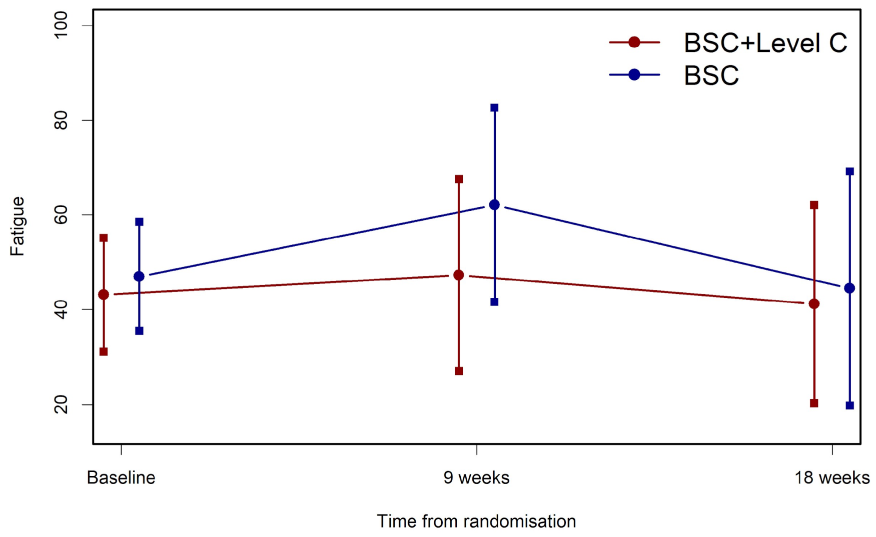

eFigure 11: EuroqoL EQ-5D-3L Score in the CHEMO-BSC pathway
(mean and 95% Confidence Intervals for baseline, adjusted mean and 95% Confidence Intervals for subsequent assessments)

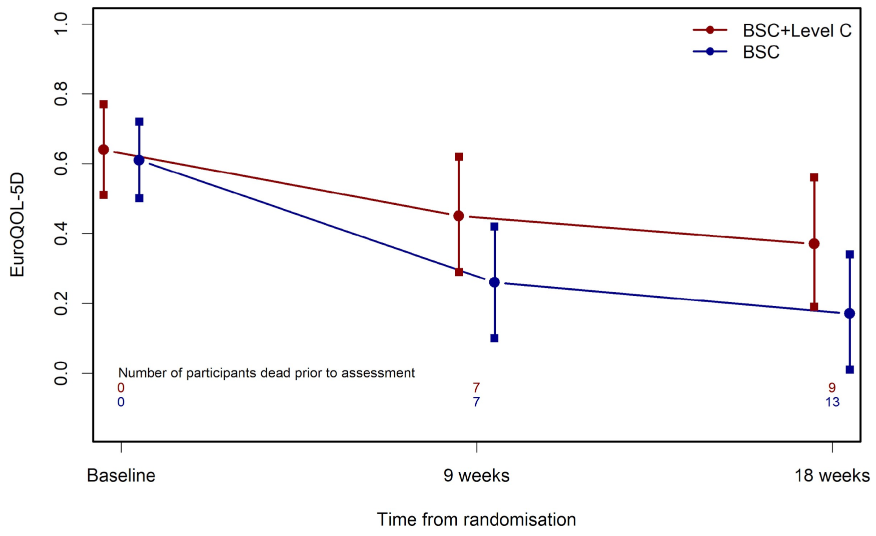

© 2021 Hall PS et al. *Jama Oncol.*

eFigure 12: Time to Deterioration of Fatigue in the CHEMO-INTENSITY Pathway

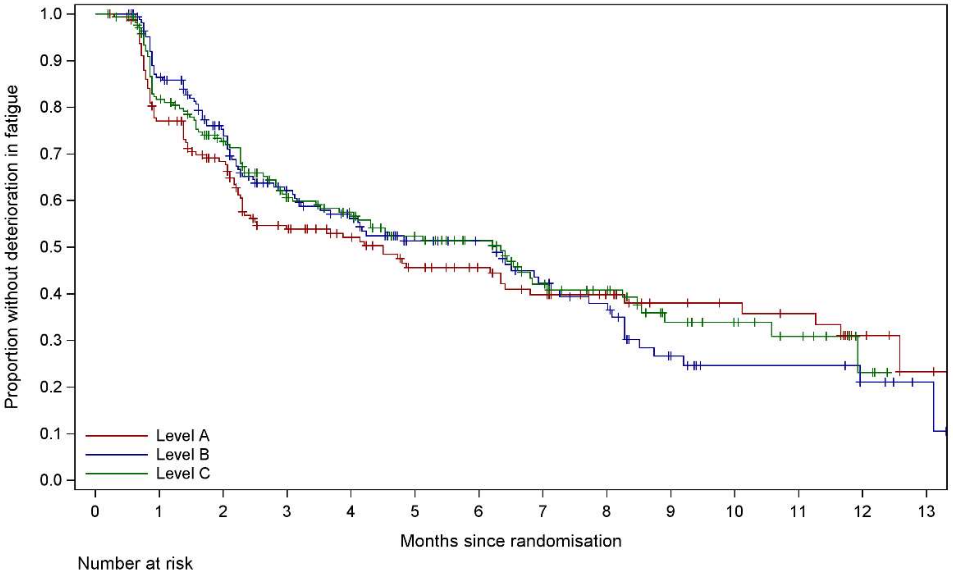

Number at risk

	0	1	2	3	4	5	6	7	8	9	10	11	12	13
Level A	163	120	97	69	59	47	40	33	25	19	17	15	6	2
Level B	169	140	105	75	62	45	41	31	26	14	8	8	5	2
Level C	171	133	108	79	70	57	48	34	28	17	13	9	3	0

eFigure 13: Per Protocol Population: PFS curves by randomised treatment arm

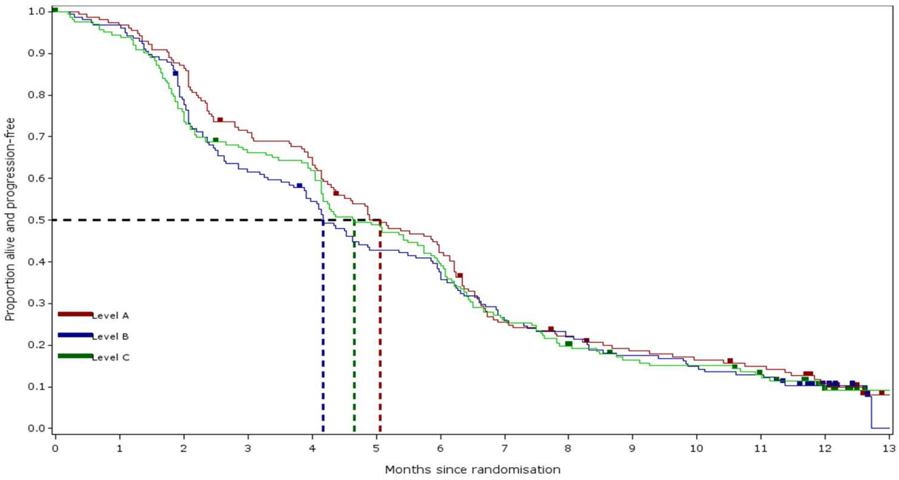

Number at Risk

	0	1	2	3	4	5	6	7	8	9	10	11	12	13
Level A	161	150	135	109	100	76	64	38	32	26	23	20	12	0
Level B	163	152	123	96	84	66	58	40	34	27	23	20	8	0
Level C	168	154	124	107	100	79	64	42	31	24	22	17	7	0

© 2021 Hall PS et al. *Jama Oncol.*
