# Supplementary material for: Efficacy of Reduced-Intensity Chemotherapy With Oxaliplatin and Capecitabine on Quality of Life and Cancer Control Among Older and Frail Patients With Advanced Gastroesophageal Cancer: The GO2 Phase 3 Randomized Clinical Trial
Source: JAMA Oncol. 2021 May 13;7(6):869–77. doi: 10.1001/jamaoncol.2021.0848 (PMC8120440; doi:10.1001/jamaoncol.2021.0848)
Supplement: Supplement 4. — Nonauthor Collaborators [file jamaoncol-e210848-s004.pdf]

Supplemental Online Content: Nonauthor Collaborators

*Indicates required information. Only first name, last name, and suffix will appear in PubMed.

Group Name(s):* The GO2 Trial Investigators							
First Name and Middle Initial(s)*	Last Name*	Suffix (eg, Jr, III)*	Academic Degrees	Institution	Location (city, state/province, country)	Role or Contribution, eg, chair, principal investigator	Group (if more than 1 Group listed in the byline) and/or Subgroup (eg, Steering Committee)
Eleanor	James			Sherwood Forest NHS Foundation Trust	Nottingham, UK	Recruiting Centre PI	
Sue	Cheeseman			Bradford Teaching Hospitals NHS Foundation Trust	Bradford, UK	Recruiting Centre PI	
Tom	Roques			Norfolk and Norwich University Hospitals NHS Foundation Trust	Norwich, UK	Recruiting Centre PI	
Nick	Reed			Imperial College Healthcare NHS Trust	London, UK	Recruiting Centre PI	
Charles	Candish			Gloucestershire Hospitals NHS Foundation Tru	Gloucester, UK	Recruiting Centre PI	
David	Fyfe			University Hospitals of Morecambe Bay NHS Foundation Trust	Lancaster, UK	Recruiting Centre PI	
Kim	Last			York Teaching Hospitals Foundation Trust	York, UK	Recruiting Centre PI	
Richard	Ellis			Royal Cornwall NHS Trust	Truro, UK	Recruiting Centre PI	
Lesley	Samuel			NHS Grampian, Aberdeen	Aberdeen, UK	Recruiting Centre PI	
Rebecca	Herbertson			Western Sussex Hospitals NHS Foundation Trust	Brighton, UK	Recruiting Centre PI	
Louise	Medley			South Devon Healthcare NHS Trust,	Torbay, UK	Recruiting Centre PI	
Kinnari	Patel			Oxford University Hospitals NHS Trust,	Oxford, UK	Recruiting Centre PI	
David	Sherriff			Plymouth Hospitals NHS Trust	Plymouth, UK	Recruiting Centre PI	
Angus	Robinson			East Sussex Healthcare NHS Trust	Brighton, UK	Recruiting Centre PI	
Pavel	Bezecny			Blackpool Teaching Hospitals NHS Trust	Preston, UK	Recruiting Centre PI	
Dunca	Wilkins			Betsi Cadwaldr University LHB	Clwyd, UK	Recruiting Centre PI	
Adam	McGeoch			Hinchingbrooke Healthcare NHS Trust	Huntingdon, UK	Recruiting Centre PI	
Daniel	Propper			Barts Cancer Institute	London, UK	Recruiting Centre PI	
Olwyn	Williams			Ysbyty Gwynedd	Bangor, UK	Recruiting Centre PI	
Serena	Hilman			Western Area Healthcare Trust,	Bristol, UK	Recruiting Centre PI	
Sherif	Raouf			Barking, Havering & Redbridge University Hospitals NHS Trust	London, UK	Recruiting Centre PI	
Claire	Hobbs			Great Western Hospitals NHS Foundation Trust	Swindon, UK	Recruiting Centre PI	
Jo	Parkinson			The Royal Bournemouth and Christchurch Hospitals NHS Foundation Trust	Bournemouth, UK	Recruiting Centre PI	
Nick	Wadd			South Tees Hospitals NHS Foundation Trust	Middlesborough, UK	Recruiting Centre PI	
W	Saku			Milton Keynes Hospital NHS Trust	Milton Keynes, UK	Recruiting Centre PI	
Victori	Kunene			Walsall Healthcare NHS Trust	Birmingham, UK	Recruiting Centre PI	
Colin	Askill			Abertawe Bro Morgannwg NHS Trust	Swansea, UK	Recruiting Centre PI	
Arshad	Jamil			Mid Cheshire Hospitals Foundation Trust	Crewe, UK	Recruiting Centre PI	
Emma	Cattell			Taunton and Somerset NHS Foundation Trust	Taunton, UK	Recruiting Centre PI	
Lauren	Gorf			Dorset Country Hospitals NHS Foundation Trust	Dorset, UK	Recruiting Centre PI	
Vallipuram	Vigneswaran			Singleton Hospital, Hywel Dda Health Board	Swansea, UK	Recruiting Centre PI	
Erica	Beaumont			Yeovil District Hospital NHS Foundation Trust	Yeovil, UK	Recruiting Centre PI	
Syed	Zubair			County Durham and Darlington NHS Foundation Trust	Darlington, UK	Recruiting Centre PI	
Elin	Jones			Hywel Dda Health Board	Haverfordwest, UK	Recruiting Centre PI	
Nicholas	Reed			Wye Valley NHS Trust	Cheltenham, UK	Recruiting Centre PI	
Alaaeldin	Shablak			Salisbury NHS Foundation Trust	Salisbury, UK	Recruiting Centre PI	
George	Bozas			Northern Lincolnshire and Goole NHS Foundation Trust	Hull, UK	Recruiting Centre PI	
Sheela	Rao			Royal Marsden NHS Trust	London, UK	Recruiting Centre PI	
Michael	Bennet			University of Leeds, Institute of Health Sciences	Leeds, UK	TMG Member	
Joanne	Askey			Leeds Teaching Hospitals NHS Trust	Leeds, UK	TMG Member	

Supplemental Online Content: Nonauthor Collaborators

*Indicates required information. Only first name, last name, and suffix will appear in PubMed.

First Name and Middle Initial(s)*	Last Name*	Suffix (eg, Jr, III)*	Academic Degrees	Institution	Location (city, state/province, country)	Role or Contribution, eg, chair, principal investigator	Group (if more than 1 Group listed in the byline) and/or Subgroup (eg, Steering Committee)
Gareth	Griffiths			University of Southampton	Southampton, UK	Independent TSC Chair	
Sally	Clive			Edinburgh Cancer Centre	Edinburgh, UK	Independent TSC member	
Vanessa	Potter			University Hospitals Coventry and Warwickshire NHS Trust	Coventry, UK	Independent TSC member	
Jean	Gall			n/a	n/a	Independent TSC patient member	
Chris	Twelves			Leeds Teaching Hospitals NHS Trust	Leeds, UK	IDMC Chair	
Matt	Sydes			MRC Clinical Trials Unit, University College	London, UK	IDMC member	
Juan	Valle			University of Manchester	Manchester, UK	IDMC member	
Jo	Webster			University of Leeds, Institute of Trials Research	Leeds, UK	Clinical Trials Unit member	
Marc	Jones			University of Leeds, Institute of Trials Research	Leeds, UK	Clinical Trials Unit member	
Fiona	Collinson			University of Leeds, Institute of Trials Research	Leeds, UK	Clinical Trials Unit member	
Julia	Brown			University of Leeds, Institute of Trials Research	Leeds, UK	Clinical Trials Unit member	
Louise	Brook			University of Leeds, Institute of Trials Research	Leeds, UK	Clinical Trials Unit member	
