# Supplementary material for: Efficacy of Reduced-Intensity Chemotherapy With Oxaliplatin and Capecitabine on Quality of Life and Cancer Control Among Older and Frail Patients With Advanced Gastroesophageal Cancer: The GO2 Phase 3 Randomized Clinical Trial
Source: JAMA Oncol. 2021 May 13;7(6):869–77. doi: 10.1001/jamaoncol.2021.0848 (PMC8120440; doi:10.1001/jamaoncol.2021.0848)
Supplement: Supplement 5. — Data Sharing Statement [file jamaoncol-e210848-s005.pdf]

Data Sharing Statement

Hall. Efficacy of Reduced-Intensity Chemotherapy With Oxaliplatin and Capecitabine on Quality of Life and Cancer Control Among Older and Frail Patients With Advanced Gastroesophageal Cancer. *JAMA Oncol*. Published May 13, 2021. doi:10.1001/jamaoncol.2021.0848

Data
Data available: Yes
Data types: Deidentified participant data, Data dictionary
How to access data: https://ctru.leeds.ac.uk/
When available: With publication

Supporting Documents
Document types: None

Additional Information
Who can access the data: Researchers approved by the Trial Management Group
Types of analyses: Research
Mechanisms of data availability: after approval of a proposal
